# Exercise Induced Endothelial Mesenchymal Transition (EndMT) Facilitates Meniscal Fibrocartilage Regeneration

**DOI:** 10.1002/advs.202403788

**Published:** 2024-09-30

**Authors:** Wenqiang Yan, Haoda Wu, Yue Wu, Zeyuan Gao, Zong Li, Fengyuan Zhao, Chenxi Cao, Jianquan Wang, Jin Cheng, Xiaoqing Hu, Yingfang Ao

**Affiliations:** ^1^ Department of Sports Medicine Peking University Third Hospital Institute of Sports Medicine of Peking University Beijing 100191 China; ^2^ Beijing Key Laboratory of Sports Injuries Beijing 100191 China; ^3^ Engineering Research Center of Sports Trauma Treatment Technology and Devices Ministry of Education Beijing 100191 China

**Keywords:** endothelial mesenchymal transition, exercise, handheld bioprinting, meniscal fibrocartilage regeneration, meniscus

## Abstract

The meniscus is a semilunar wedge‐shaped fibrocartilage tissue within the knee joint that is important for withstanding mechanical shock during joint motion. The intrinsic healing capacity of meniscus tissue is very limited, which makes meniscectomy the primary treatment method in the clinic. An effective translational strategy for regenerating the meniscus after total or subtotal meniscectomy, particularly for extensive meniscal lesions or degeneration, is yet to be developed. The present study demonstrates that the endothelial mesenchymal transition (EndMT) contributes to meniscal regeneration. The mechanical stimulus facilitated EndMT by activating TGF‐β2 signaling. A handheld bioprinter system to intraoperatively fabricate a porous meniscus scaffold according to the resected meniscus tissue is developed; this can simplify the scaffold fabrication procedure and period. The transplantation of a porous meniscus scaffold combined with a postoperative regular exercise stimulus facilitated the regeneration of anisotropic meniscal fibrocartilaginous tissue and protected the joint cartilage from degeneration in an ovine subtotal meniscectomy model. Single‐cell RNA sequencing and immunofluorescence co‐staining analyses further confirmed the occurrence of EndMT during meniscal regeneration. EndMT‐transformed cells gave rise to fibrochondrocytes, subsequently contributing to meniscal fibrocartilage regeneration. Thus, an efficient translational strategy to facilitate meniscal regeneration is developed.

## Graphical abstract

A handheld bioprinter system to intraoperatively fabricate porous meniscus scaffold is developed. The transplantation of meniscus scaffold combining postoperative regular exercise facilitated anisotropic meniscal fibrocartilage regeneration in an ovine meniscectomy model. Mechanistically, mechanical stimuli facilitated the transition of endothelial cells within scaffold into mesenchymal cells, called EndMT. EndMT‐transformed cells gave rise to fibrochondrocytes, subsequently contributing to meniscal fibrocartilage regeneration.

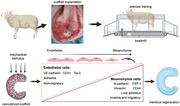

## 1 Introduction

The meniscus is a semilunar wedge‐shaped fibrocartilage tissue that provides structural consistency between the distal femur and proximal tibia and absorbs mechanical impact during knee joint movements.^[^ ^1^ ^]^ Meniscal injuries are one of the most common knee joint related injuries.^[^ ^2^ ^]^ The healing capacity of the meniscus demonstrates regional differences, which are now thought to be heavily dependent on blood supply.^[^ ^3^ ^]^ The formation of blood vessels in meniscus tissue varies at different developmental stages. From the prenatal period to shortly after birth, the entire meniscus is rich in blood vessels. Subsequently, vascularization is concentrated in the peripheral area (10%–30%) at the age of 10 years. In adults, blood supply only exists in the periphery 10%–25% area).^[^ ^4^ ^]^ Subsequently, it is possible to distinguish between the surrounding vascular zone (red‐red zone) and the completely avascular inner zone (white‐white zone). The red‐white zone is located intermediately. The current popular strategy used for the treatment of meniscus tears is to maintain the integrity of the meniscus tissue as much as possible. Meniscal repair using sutures is typically performed for meniscal lesions in the peripheral vascular zone.^[^ ^3a^ ^]^ However, meniscal lesions in the avascular zone inevitably require resection because of their limited healing ability.^[^ ^5^ ^]^ Consequently, the mechanical balance of the knee joint is disrupted due to the disruption of meniscal integrity after meniscectomy. The contact area between the femoral condyle and corresponding tibial plateau decreased. Thus, the contact stress on the cartilage increases sharply, leading to secondary cartilage deterioration or osteoarthritis (OA).^[^ ^6^ ^]^ Therefore, research interest has shifted to promoting the repair or regeneration of meniscal injuries in the avascular zone to maintain the integrity of the meniscal tissue, prevent secondary cartilage deterioration, and improve knee joint function.

Recently, several studies have reported that meniscal tissue regeneration can be achieved using tissue engineering strategies based on 3D printing technology for the fabrication of biomimetic meniscus scaffolds.^[^ ^7^ ^]^ Zhang et al.^[^ ^7a^ ^]^ constructed an anisotropic meniscus tissue in vitro under dual stimuli on mesenchymal stem cells (MSCs) seeded into a 3D printing biomimetic scaffold, including biomechanical stimulus imposed by a customized dynamic tension‐compression loading system and biochemical stimulus using TGF‐β and CTGF growth factors. Lee et al.^[^ ^7b^ ^]^ developed a spatial TGF‐β3 and CTGF releasing system in a 3D‐printed meniscus scaffold. The spatially delivered TGF‐β3 and CTGF enabled the heterogeneous differentiation of endogenous stem/progenitor cells, thus facilitating anisotropic meniscus regeneration in a sheep meniscectomy model. Thus, meniscal tissue engineering based on 3D printing technology is a promising method for regenerating menisci. However, there are still obstacles that restrict their clinical application. First, the current studies usually acquired meniscus imaging data, such as computed tomography (CT) or magnetic resonance imaging (MRI), followed by reconstruction of 3D printing meniscus model. The meniscus scaffold planned for implantation was fabricated in advance. However, in the clinic, the meniscal tissue is resected based on intraoperative observation and damage situation, which would be inconsistent with the preoperative plan. Therefore, the prepared meniscus scaffold would be mismatched with the remaining meniscal tissue and articular cartilage, which could cause adverse effects such as scaffold fracture and cartilage damage. To solve this problem, we fabricated a porous meniscus scaffold intraoperatively according to the resected meniscus tissue morphology using a handheld bioprinter, which not only restored the original meniscal defect morphology but also matched the remaining meniscal tissue and articular cartilage. Second, current meniscal tissue engineering studies usually seed MSCs, fibrochondrocytes or chondrocytes^[^ ^8^ ^]^ into porous scaffolds. However, adverse effects, including the tumorigenicity of MSCs and the immune rejection of allogeneic cells, cannot be neglected.^[^ ^8^, ^9^ ^]^ Seeding cells from autologous tissues to avoid immune rejection has also been investigated. However, extra injury to the donor site during cell harvesting and cell phenotype dedifferentiation during in vitro expansion culture cannot be neglected.^[^ ^8b^ ^]^ Thus, future studies should focus on meniscal regeneration by enhancing the function of endogenous repairable cells. A previous study revealed MSCs participate in the natural maturation and regeneration of the meniscus, demonstrating that MSCs serve as critical intrinsic healing cells during meniscal regeneration.^[^ ^10^ ^]^ Interestingly, after careful observation of the regenerated meniscus tissue in previous studies, we found that the newly formed meniscus‐like tissue contained not only fibrocartilaginous and fibrous tissue but also rich fibrovascular tissue, especially in the early regeneration phase,^[^ ^7^, ^10^, ^11^ ^]^ similar to the early developmental stage of native menisci. The infiltration of blood vessels not only brings oxygen and nutrients, but also facilitates metabolite exchange, which benefits tissue repair or regeneration. However, the potential roles of vascular cellular components such as endothelial cells (ECs) in meniscal regeneration have not been investigated. Interestingly, vascular ECs demonstrate considerable phenotypic plasticity to generate other cell types under certain conditions through a cell differentiation mechanism known as endothelial‐mesenchymal transition (EndMT).^[^ ^12^ ^]^ During EndMT, the expression levels of endothelial markers, including VE‐cadherin, CD31, TIE1, TIE2, and vWF decreased, while those of the mesenchymal markers FSP‐1, α‐SMA, N‐cadherin, and vimentin increased. Distinct changes in cell polarity and morphology were accompanied by EndMT, loss of cell‐cell junctions, and increased motility. Mesenchymal cells (MCs) generated by EndMT exhibit a stem cell phenotype and can differentiate into other cell lineages.^[^ ^12^ ^]^ Suzuki et al.^[^ ^13^ ^]^ concluded that EndMT‐derived pulmonary vascular ECs during the early phase of acute lung injury caused by reactive oxygen species (ROS) activation share progenitor cell‐like characteristics that contribute to repair after pulmonary vascular injury. Moreover, mechanical stimuli can trigger EndMT in ECs.^[^ ^14^ ^]^ The infiltrated fibrovascular tissue within the meniscal scaffold experiences a mechanical stimulus imposed by knee joint compression. Thereby, we hypothesized EndMT process existed during meniscal regeneration and contributed to meniscal regeneration. Interestingly, we confirmed the EndMT process during meniscal regeneration using the *CDH5‐CreER^T2^; Rosa26‐LSL‐ tdTomato* endothelial cell lineage tracing transgenic mouse model. Thus, we hypothesized that generating appropriate mechanical stimuli within the knee joint, such as regular exercise rehabilitation, could be beneficial for meniscal regeneration by enhancing EndMT, which could avoid the transplantation of autologous or allogeneic cells.

Herein, subtotal meniscectomy (90% excision) was performed in an ovine model, which represents extensive resection for meniscal lesions in the clinic. A biomimetic porous meniscus scaffold fabricated intraoperatively according to the resected meniscus tissue morphology using a handheld bioprinter was implanted and fixed anatomically. Regular exercise rehabilitation was performed postoperatively to provide appropriate mechanical stimulus to the knee joint. The degree of meniscal regeneration and chondroprotection was evaluated 4 months postoperatively. The potential molecular mechanisms underlying mechanical stimulus‐facilitated EndMT have been clarified. Single‐cell RNA sequencing (scRNA‐Seq) was used to identify cell populations and gene expression profiles during meniscal regeneration. Interestingly, pseudo‐time trajectory analysis revealed that EndMT gave rise to fibrochondrocytes during meniscal regeneration. Abbreviations used in this study are summarized in Table S1 (Supporting Information).

## 2 Results

### 2.1 Endothelial Mesenchymal Transition (EndMT) in Meniscal Regeneration

**Figure 1:**
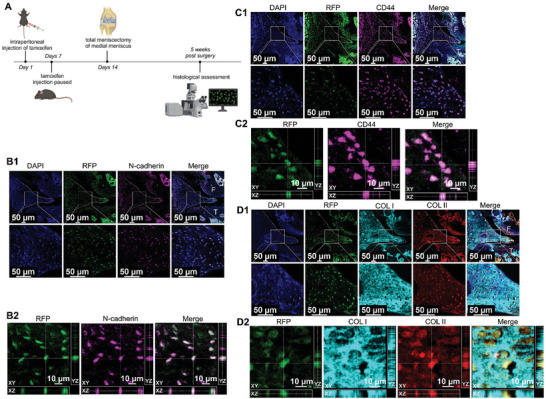
Evaluation of EndMT during meniscal regeneration using the endothelial cell lineage tracing transgenic mouse meniscectomy model. A) Flow chart depicting the operations in *CDH5‐CreER^T2^; Rosa26‐LSL‐tdTomato* endothelial cell lineage tracing transgenic mice. B) Immunofluorescent colocalization of RFP and N‐cadherin within the intrinsic healed tissue in transgenic mice after total meniscectomy (B1, immunofluorescence colocalization in 2D view; B2, orthogonal projection of RFP and N‐cadherin fluorescence in 3D view). C) Immunofluorescent colocalization of RFP and CD44 within the intrinsic healed tissue in transgenic mice after total meniscectomy (C1, immunofluorescence colocalization in 2D view; C2, orthogonal projection of RFP and CD44 fluorescence in 3D view). D) Immunofluorescent colocalization of RFP, COL I and COL II within the intrinsic healed tissue in transgenic mice after total meniscectomy (D1, immunofluorescence colocalization in 2D view; D2, orthogonal projection of RFP, COL I and COL II fluorescence in 3D view). F represents the femur; T represents the tibia.

To investigate the phenomenon of EndMT during meniscal regeneration, small animals, such as mice with a relatively robust intrinsic repair capacity were used. To confirm EndMT during meniscal regeneration, *CDH5‐CreER^T2^; Rosa26‐LSL‐ tdTomato* endothelial cell lineage tracing transgenic mice were used to trace ECs.^[^ ^15^ ^]^ The *CDH5* positive cells expressed tdTomato proteins (a type of red fluorescent protein, RFP) after tamoxifen induction. Tamoxifen induction was initiated 8 weeks after birth for 7 days. A total meniscectomy model of the medial meniscus was prepared 14 d after tamoxifen induction. Knee samples were collected 5 weeks after surgery (**Figure** **1**A). EndMT during meniscal regeneration was evaluated by immunofluorescence colocalization. Native meniscus‐like tissue sprouted between the femur and the tibia. First, robust expression of the mesenchymal marker (N‐cadherin)^[^ ^12^ ^]^ was observed within the regenerated tissue and co‐localized with RFP signals (Figure 1B1). Moreover, the orthogonal projection of RFP and N‐cadherin fluorescence in the 3D view further confirmed their colocalization (Figure 1B2). Second, robust expression of mesenchymal stem cell (MSCs) marker (CD44)^[^ ^12^ ^]^ was observed within the regenerated tissue and co‐localized with RFP signals (Figure 1C1). Moreover, the orthogonal projection of RFP and CD44 fluorescence in the 3D view further confirmed their colocalization (Figure 1C2). Third, robust expression of fibrochondrogenic markers (COL I and COL II) was observed within the newly formed tissue and co‐localized with RFP signals (Figure 1D1). Moreover, the orthogonal projection of RFP, COL I, and COL II fluorescence in a 3D view further confirmed colocalization (Figure 1D2). In conclusion, ECs undergo EndMT during meniscal regeneration and differentiate into fibrochondrocyte‐like cells. Finally, immunofluorescence co‐staining of lineage tracing markers (RFP), endothelial markers (CDH5), and mesenchymal/stem markers (N‐cadherin and CD44) was performed to comprehensively evaluate the participation of EndMT during meniscal regeneration. Immunofluorescence results demonstrated multiple EndMT statuses during meniscal regeneration. The blank EndMT (RFP+, CDH5+, N‐cadherin‐, CD44‐), partial EndMT (RFP+, CDH5+, N‐cadherin+, CD44+) and full EndMT (RFP+, CDH5‐, N‐cadherin+, CD44+) could be observed within the regenerated meniscus tissue (Figures S1 and S2, Supporting Information).

### 2.2 Mechanical Stimulus Exerted by Knee Joint Affects Meniscal Maturation and Phenotype Maintenance

The mechanical stimuli imposed by the knee joint play a critical role in meniscal maturation. In the present study, the maturation process of rabbit menisci was evaluated. Meniscal cell morphology and fiber arrangement during maturation were analyzed. The morphology of meniscal cells was uniform and consistent during the early developmental stages (postnatal days 2 and 9). As the meniscus matured, the cell morphology became anisotropic between the inner and outer zones. Meniscal cells in the inner zone demonstrated an oval‐to‐round chondrocyte‐like morphology with a typical lacunar structure. The meniscal cells in the outer zone demonstrated an elongated, spindle fibroblast‐like morphology with no lacunar structure. With meniscus maturation, the collagen fibers within the meniscus tended to mature and the fiber arrangement became orderly (Figure S3, Supporting Information). We evaluated the deposition of GAG on the menisci. In the early developmental stages, GAG were concentrated at the corner of the inner edge. As the meniscus matured, the deposition of GAG increased significantly and extended to the intermediate zone, but was still absent in the peripheral outer zone. Thus, GAG were mainly distributed in the inner and intermediate zones, where knee joint compression was concentrated (Figure S4, Supporting Information). Similarly, COL II deposition demonstrated a maturation process similar to that of GAG, which was mainly distributed in the inner and intermediate zones (Figure S5, Supporting Information).

To investigate the critical role of mechanical stimuli in meniscal maturation, condylectomy of medial femoral condyle (MFC) was performed in juvenile rabbits (2 weeks after birth) (Figure S6A, Supporting Information). The medial menisci were harvested for histological analysis 6 weeks after surgery. Meniscal maturation is prominently affected in the absence of joint compression. Compared with the contralateral normal meniscus, the inner weight‐bearing area of the meniscus disappeared in the condylectomy knee, and only the peripheral area was maintained (Figure S6B, Supporting Information). The GAG deposition in the meniscus was significantly affected in the absence of a mechanical stimulus (Figure S7, Supporting Information). COL II deposition in the meniscal matrix was also significantly affected (Figure S8, Supporting Information). Thus, the mechanical stimulus exerted by joint compression affects meniscal maturation in terms of morphology, fibrochondrogenic phenotype, and matrix deposition. To further investigate the critical role of mechanical stimuli in meniscal phenotype maintenance, condylectomy of the MFC was performed in adult rabbits (6 months after birth) (Figure S9A, Supporting Information). Although the morphology of the meniscus was not affected, the GAG content decreased significantly in the absence of joint compression (Figure S9B–D, Supporting Information). The content of COL II in the meniscus matrix was not significantly affected after condylectomy during the observation period of 6 weeks (Figure S10, Supporting Information). However, after careful observation, the meniscal cells after condylectomy were negative for COL II immunostaining, demonstrating a loss of the chondrogenic phenotype (Figure S10A, Supporting Information). Therefore, the mechanical stimuli imposed by joint compression affect meniscal phenotype maintenance in adults.

### 2.3 Mechanical Stimulus Facilitates EndMT Through TGF‐β2 Signaling

**Figure 2:**
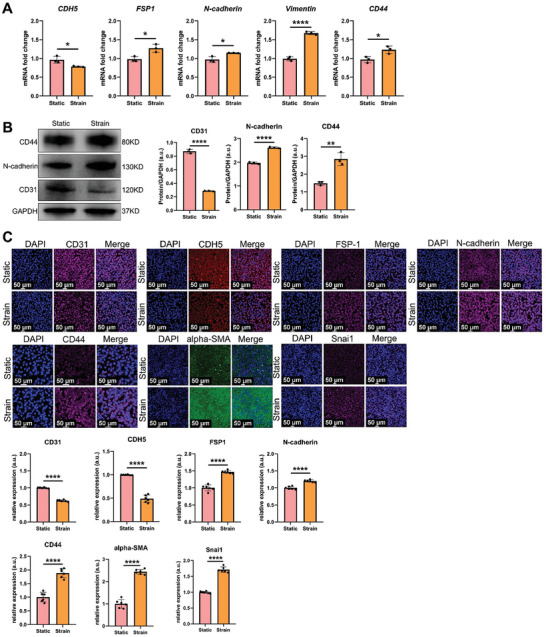
Mechanical stimulus facilitates the occurrence of EndMT in HUVECs. A) The mRNA level of HUVECs after cyclic tensile strain stimulus, n = 3, unpaired *t*‐test. B) Protein levels of HUVECs after cyclic tensile strain stimulus, as evaluated by western blotting (n = 3; unpaired *t*‐test). C) Protein levels of HUVECs after cyclic tensile strain stimulus, as evaluated by immunofluorescence analysis; six ROIs per biological sample were quantified (unpaired *t*‐test). a.u. represents arbitrary unit, ROIs represents region of interests, n represents the sample size, * represents *p*<0.05, ** represents *p*<0.01, and **** represents *p*<0.001.

**Figure 3:**
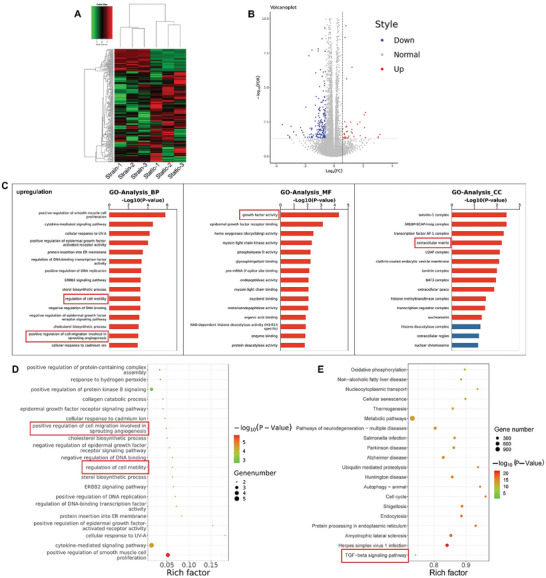
RNAseq analysis of HUVECs after mechanical stimulus. A) Differentially expressed gene clusters among samples of static and strain stimulus group. B) Volcano plot of differentially expressed genes among samples of the static and strain stimulus group. C) Bar plot of GO (Gene Ontology) enrichment analysis, the red box represents regulation of cell motility, positive regulation of cell migration involved in sprouting angiogenesis, growth factor activity and extracellular matrix. D) Bubble plot of GO enrichment analysis: the red box represents the regulation of cell motility; the positive regulation of cell migration involved in sprouting angiogenesis. E) Bubble plot of Kyoto encyclopedia of genes and genomes (KEGG) pathway enrichment analysis: the red box represents TGF‐β signaling pathway.

**Figure 4:**
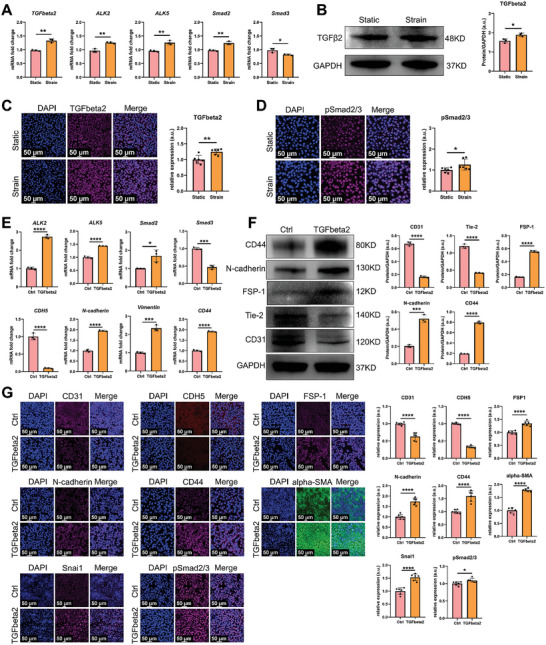
Mechanical stimulus facilitates the occurrence of EndMT in HUVECs via TGF‐β2 signaling. A) The mRNA level of HUVECs after cyclic tensile strain stimulus (n = 3; unpaired *t*‐test). B) The protein level of TGF‐β2 in HUVECs after cyclic tensile strain stimulus, as evaluated by western blotting (n = 3; unpaired *t*‐test). C) Protein level of TGF‐β2 in HUVECs after cyclic tensile strain stimulus, as evaluated by immunofluorescence analysis; six ROIs per biological sample were quantified (unpaired *t*‐test). D) Protein level of pSmad2/3 in HUVECs after cyclic tensile strain stimulus, as evaluated by immunofluorescence; six ROIs per biological sample were quantified (unpaired *t*‐test). E) The mRNA level of HUVECs after treatment with TGF‐β2 (n = 3; unpaired *t*‐test). F) Protein level of HUVECs after treatment with TGF‐β2, as evaluated by western blotting (n = 3; unpaired *t*‐test). G) Protein level of HUVECs after treatment with TGF‐β2, as evaluated by immunofluorescence; six ROIs per biological sample were quantified (unpaired *t*‐test). a.u. represents arbitrary unit, ROIs represents region of interests, n represents the sample size, * represents *p*<0.05, ** represents *p*<0.01, *** represents *p*<0.005, and **** represents *p*<0.001.

EndMT can be observed in the native meniscus and contributes to meniscal development. Moreover, the mechanical stimulus imposed by joint compression played critical role in meniscal maturation and phenotype maintenance.^[^ ^7^, ^10^ ^]^ We hypothesized that mechanical stimuli affect EndMT. Human umbilical vein endothelial cells (HUVECs) were used to investigate the effects of biomechanical stimuli on EndMT and its potential mechanisms. Cyclic tensile strain (CTS) was used in vitro to simulate biomechanical stimuli on HUVECs. A previous study demonstrated that native menisci have a mean strain of 5% under 100% weight‐bearing conditions.^[^ ^16^ ^]^ Thus, a periodic 5% strain was applied to HUVECs for 12 h using an in vitro Flexcell train culture system. After the CTS treatment of HUVECs, the mRNA levels of the EC‐specific gene *CDH5* were significantly downregulated. The mRNA levels of the MC biomarkers (*FSP‐1, N‐cadherin*, and *Vimentin*) were significantly upregulated. The mRNA levels of the MSC‐specific gene *CD44* were significantly upregulated (**Figure** **2**A). Protein levels were evaluated using western blotting. The protein level of the EC‐specific marker CD31 was significantly downregulated. The protein levels of the MC‐specific marker N‐cadherin and MSC‐specific marker CD44 were also significantly upregulated (Figure 2B). Protein levels were evaluated using immunofluorescence. The immunofluorescence results were consistent with those of mRNA and western blotting, demonstrating a decrease in the levels of EC‐specific markers (CD31 and CDH5) and an increase in the levels of the markers of MCs (FSP‐1, N‐cadherin, and alpha‐SMA) and MSCs (CD44) and the EndMT transcription factor Snai1^[^ ^17^ ^]^ (Figure 2C). To further investigate the potential mechanisms of mechanical stimuli in EndMT, we performed RNA‐seq analyses of HUVECs after treatment with CTS or static conditions. These genes were differentially expressed after CTS treatment (**Figure** **3**A,B). The Gene ontology (GO) analyses of biological processes (BPs) demonstrated that the regulation of cell motility and positive regulation of cell migration involved in sprouting angiogenesis were upregulated. Further, MCs are more invasive and demonstrate higher migration abilities than ECs,^[^ ^12^ ^]^ which confirmed EndMT in HUVECs after CTS treatment. The GO analyses of molecular functions (MF) demonstrated that growth factor activity was upregulated. GO analysis of the cellular components (CCs) demonstrated the extracellular matrix was upregulated (Figure 3C,D). The Kyoto encyclopedia of genes and genomes (KEGG) pathway analysis revealed the TGF‐β signaling pathway was involved during EndMT induced by mechanical stimulus (Figure 3E). Previous studies have demonstrated the TGF‐β/BMP family of growth factors could stimulate EndMT, especially the TGF‐β2.^[^ ^17^ ^]^ After CTS treatment, the mRNA levels of *TGF‐β2*, activin‐like kinases (*ALK2* and *ALK5*) and downstream *Smad2* in HUVECs were upregulated significantly. However, the mRNA level of *Smad3* was downregulated significantly (**Figure** **4**A). Next, we evaluated the protein levels of TGF‐β2 signaling in HUVECs after CTS treatment. The protein levels of TGF‐β2 and pSmad2/3 were upregulated significantly (Figure 4B–D). Then, we further evaluated the effects of TGF‐β2 treatment on EndMT. After TGF‐β2 treatment in HUVECs, the mRNA levels of TGF‐β signaling including *ALK2, ALK5, Smad2* were upregulated significantly. However, the mRNA level of *Smad3* was significantly downregulated, which was identical to that observed after CTS treatment. Previous study demonstrated the downregulation of Smad3 expression formed a negative feedback loop of TGF‐β signaling to avoid excessive activation of the signaling.^[^ ^18^ ^]^ The mRNA levels of the EC‐specific marker *CDH5* was significantly downregulated. The mRNA levels of MC markers (*N‐cadherin* and *Vimentin*) were significantly upregulated. The mRNA level of the MSC marker *CD44* was significantly upregulated (Figure 4E). Protein levels were also evaluated by western blotting and immunofluorescence, demonstrating the downregulation of EC‐specific markers and the upregulation of markers specific for MCs, MSCs, and EndMT transcription factors (Figure 4F,G). SB‐431542 is a TGF‐β receptor kinase inhibitor.^[^ ^19^ ^]^ Suppression of EndMT by SB431542 reversed the formation of molecular features induced by the mechanical stimulus in HUVECs, which further confirmed the role of TGF‐β signaling in inducing EndMT (Figure S11, Supporting Information).

### 2.4 EndMT Transformed Cells Transplantation Facilitates Orthotopic and Ectopic Meniscal Regeneration

**Figure 5:**
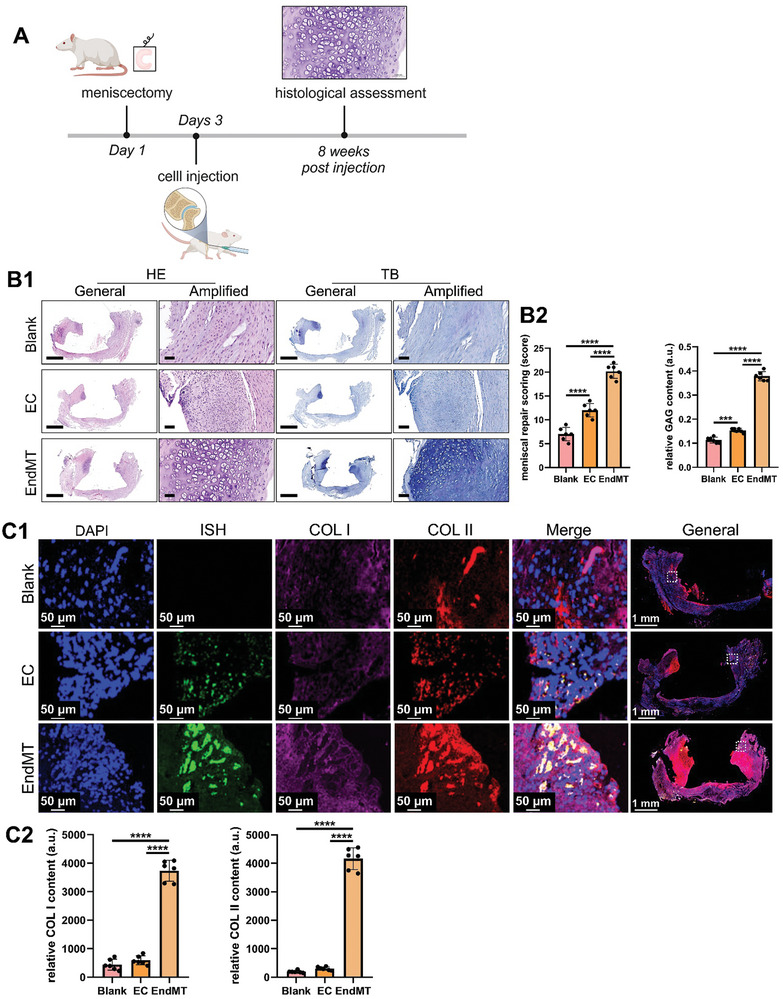
Transplantation of EndMT‐transformed cells facilitates orthotopic meniscal regeneration in a rat model. A) Flowchart of the transplantation of EndMT‐transformed cells in a rat orthotopic meniscectomy model. B) Histological analysis of orthotopic meniscal regeneration (B1, histological staining of the repaired tissue in each group: scale bar of the general images: 1 mm, scale bar of amplified images: 0.05 mm; B2, meniscal repair scoring and semiquantitative analysis of GAG content in each group: one ROI per biological sample was quantified (n = 6, one‐way ANOVA)). C) Co‐staining of in situ hybridization (ISH) for the SRY gene and COL I and COL II in the repaired tissues in each group (C1, immunofluorescence co‐staining; C2, semiquantitative analysis of COL I and COL II contents: one ROI per biological sample was quantified (n = 6, one‐way ANOVA); the SRY gene represents the male‐specific sex‐determining region Y‐linked gene). EC represents endothelial cell, EndMT represents endothelial mesenchymal transition, GAG represents glycosaminoglycans, COL I represents type I collagen, COL II represents type II collagen, a.u. represents arbitrary unit, ROI represents region of interest, n represents the sample size, *** represents *p*<0.005, and **** represents *p*<0.001.

To further investigate the role of EndMT‐transformed cells in meniscal fibrocartilage regeneration, we established an orthotopic meniscal regeneration rat model. The endothelial cells isolated from rabbit aorta demonstrated high purity, which was verified by flow cytometry analysis on CD31 marker (Figure S12A, Supporting Information). The EndMT status was effectively achieved in rabbit ECs after treatment with TGF‐β2 for 4 days (Figure S12B, Supporting Information). A total meniscectomy model of the medial meniscus was prepared, followed by injection of EC‐ or EndMT‐transformed cells. Eight weeks after cell injection, meniscal regeneration was evaluated by histological analysis (**Figure** **5**A). In the Blank or EC groups, the regenerated tissue was mostly composed of fibrous tissue and a small amount of cartilaginous tissue. Only a few small cells with ovoid or circular nuclei are observed in the regenerated tissue. The extracellular matrix (ECM) within the regenerated tissue showed negative or weak staining with toluidine blue, indicating poor deposition of GAG. However, in the EndMT group, the newly formed tissue contained most of the cartilaginous tissue. Large round or ovoid meniscal fibrochondrocyte‐like cells with rich cytoplasm were observed within the regenerated tissue. The newly formed tissue exhibited robust toluidine blue staining, indicating rich GAG deposition. Moreover, the EndMT group had higher meniscal repair scores than the Blank and EC groups (Figure 5B). Next, we evaluated the deposition of fibrocartilaginous collagen matrix, including COL I and COL II. The regenerated tissue in the EndMT group showed the most robust deposition of COL I and COL II. In the present study, ECs from male rabbits were used to trace the fate of transplanted cells. In situ hybridization (ISH) of the male‐specific sex‐determining region Y‐linked (SRY) gene in male rabbits was used to distinguish transplanted cells. No ISH signal was observed in the Blank group, demonstrating the specificity of the SRY probe. In the EC and EndMT groups, apparent ISH signals were observed within the regenerated tissue, indicating that the injected ECs and EndMT‐transformed cells participated in the meniscal regeneration. Moreover, the co‐localization of the ISH signal for the SRY gene and COL I and II confirmed the differentiation of EndMT‐transformed cells into fibrochondrocyte‐like cells (Figure 5C). Thus, EndMT transformed cells transplantation facilitated orthotopic meniscal regeneration.

**Figure 6:**
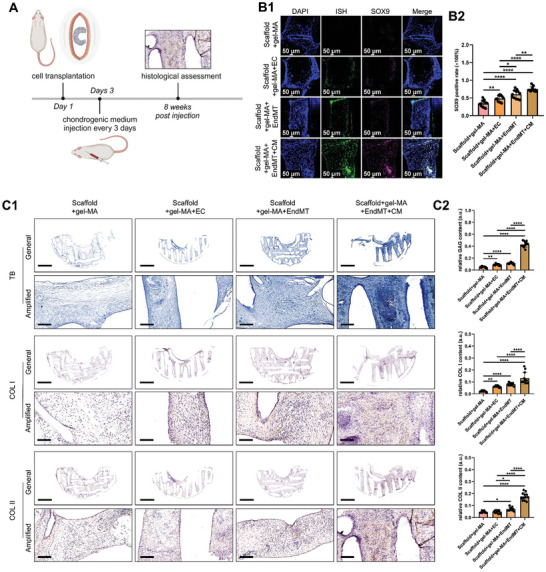
Transplantation of EndMT‐transformed cells facilitates ectopic meniscal regeneration in a rat model. A) Flowchart of the transplantation of EndMT‐transformed cells in a rat ectopic meniscal regeneration model. B) Co‐staining of ISH for the SRY gene and SOX9 in the regenerated tissues in each group (B1, immunofluorescence co‐staining; B2, semiquantitative analysis of SOX9‐positive regions within the regenerated tissues: one ROI per biological sample was quantified (n = 12, one‐way ANOVA)). C) Histological and immunohistochemical assessment of meniscal matrix deposition in each group (C1, TB staining for GAG evaluation and the immunohistochemical staining of COL I and COL II: TB represents toluidine blue, General represents the general figures of staining, and Amplified represents the representative magnified area of the general figures. Scale bar of general images: 2 mm; scale bar of amplified images: 0.2 mm; C2, semiquantitative analysis of GAG, COL I, and COL II within the regenerated tissues in each group: one ROI per biological sample was quantified (n = 12, one‐way ANOVA)). EC represents endothelial cell, EndMT represents endothelial mesenchymal transition, GAG represents glycosaminoglycans, COL I represents type I collagen, COL II represents type II collagen, a.u. represents arbitrary unit, ROI represents the region of interest, n represents the sample size, * represents *p*<0.05, ** represents *p*<0.01, and **** represents *p*<0.001.

Next, an ectopic meniscal regeneration model was established on the back of the rat. A semilunar meniscus‐like porous scaffold was prepared by 3D printing. The ECs or EndMT‐transformed cells were encapsulated with the gel‐MA hydrogel and then filled in the scaffold pores, followed by subcutaneous implantation in the rat back. To induce chondrogenic differentiation of EndMT‐transformed cells, chondrogenic medium (CM) was injected into the scaffold via subcutaneous injection every three days. Eight weeks after the injection, all samples were collected for histological analysis to evaluate fibrocartilage regeneration (**Figure** **6**A). Transplanted cells were harvested from male rabbits. First, we used ISH for SRY gene to trace the transplanted cells. There was no ISH signal in the Scaffold+gel‐MA group, but the signal was apparent in the cell transplantation group. We performed immunofluorescence co‐staining of ISH and SOX9 to confirm the chondrogenic differentiation of EndMT‐transformed cells. Apparent and more robust SOX9 expression was observed in EndMT‐transformed cells after induction with chondrogenic medium (Scaffold+gel‐MA+EndMT+CM group) (Figure 6B). The fibrocartilaginous matrix deposition was then evaluated. The Scaffold+gel‐MA+EndMT+CM group showed the most robust staining with toluidine blue, COL I, and COL II, demonstrating superior fibrocartilage production (Figure 6C). Therefore, the EndMT transformed cell transplantation combining fibrochondrogenic induction facilitated ectopic meniscal regeneration.

### 2.5 Transplantation of the Porous Scaffold Fabricated by a Handheld 3D Bioprinter Combining Regular Exercise Facilitates Meniscal Fibrocartilage Regeneration and Protects Cartilage in an Ovine Subtotal Meniscectomy Model

**Figure 7:**
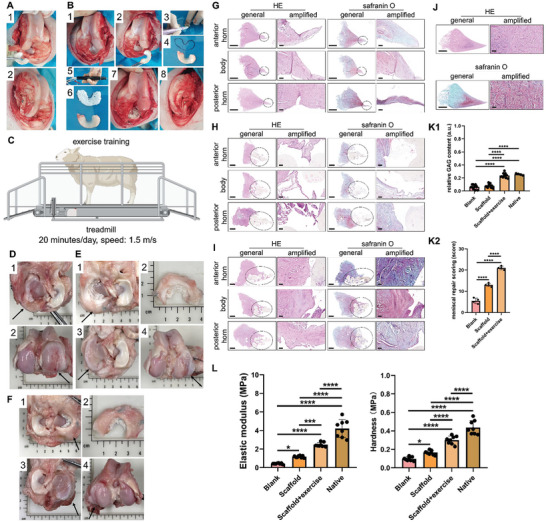
Transplantation of the porous scaffold fabricated by a handheld 3D bioprinter, combined with regular exercise, facilitates meniscal fibrocartilage regeneration and protects cartilage in an ovine subtotal meniscectomy model. A) Preparation of subtotal meniscectomy in an ovine model (1, subtotal meniscectomy of the medial meniscus; 2, fixation of the bone block of the medial collateral ligament superior attachment using nails). B) Intraoperative preparation of porous meniscus scaffold using a handheld bioprinter (1, exposure of the medial meniscus; 2, subtotal meniscectomy of the medial meniscus; 3, outline drawing of the resected meniscus tissue; 4, contour of the resected meniscus tissue; 5, preparation of the handheld bioprinter: the red arrow represents the polyurethane wire used for printing; 6, fabricated porous meniscus scaffold; 7, implantation and fixation of the meniscus scaffold; 8, fixation of the bone block of the medial collateral ligament superior attachment using nails). C) Schematics of exercise training of ovine in the treadmill. D) Macroscopic evaluation of knee joint in the Blank group (1, tibial plateau; 2, femoral condyle, black arrow represents medial side of joint). E) the macroscopic evaluation of knee joint in the Scaffold group (1, tibial plateau with scaffold; 2, scaffold and regenerated tissue; 3, tibial plateau cartilage; 4, femoral condyle cartilage, black arrow represents medial side of joint). F) Macroscopic evaluation of the knee joint in the Scaffold+exercise group (1, tibial plateau with scaffold; 2, scaffold and regenerated tissue; 3, tibial plateau cartilage; 4, femoral condyle cartilage: the black arrow represents the medial side of joint). G) Histological analysis of regenerated tissues in the Blank group: the black circle represents the newly regenerated tissue; scale bar of the general images: 2 mm and scale bar of the amplified images: 0.1 mm. H) Histological analysis of regenerated tissues in the Scaffold group: the black circle represents the newly regenerated tissue; scale bar of the general images: 2 mm and scale bar of the amplified images: 0.1 mm. I) Histological analysis of regenerated tissues in the Scaffold+exercise group: the black circle represents the newly regenerated tissues; scale bar of the general images: 2 mm and scale bar of the amplified images: 0.1 mm. J) Histological analysis of the native meniscus tissues in the Sham group: scale bar of the general images: 2 mm and scale bar of the amplified images: 0.1 mm. K) Semiquantitative analysis of newly regenerated and native tissues (K1, semiquantitative analysis of GAG content, for the Blank, Scaffold, and Scaffold+exercise groups: a total of 12 ROIs in each group were quantified; for the Native group, a total of 4 ROIs were quantified (one‐way ANOVA); K2, meniscal repair scoring (n = 4, one‐way ANOVA)). L) Mechanical properties of newly regenerated tissues and native meniscus tissues; a total of eight points per group were tested (one‐way ANOVA). a.u. represents arbitrary unit, n represents the sample size, GAG represents glycosaminoglycans, * represents *p*<0.05, *** represents *p*<0.005, and **** represents *p*<0.001.

We confirmed that EndMT contributes to native meniscal development and regeneration. Moreover, the mechanics of the knee joint affect meniscal maturation and phenotype maintenance. Furthermore, mechanical stimuli promote the transition of ECs into MCs with a stem cell phenotype. Mesenchymal stem cells can differentiate into fibrochondrocytes under mechanical stimuli.^[^ ^10^ ^]^ Therefore, to further investigate the effect of knee joint mechanics exerted by a regular exercise stimulus on meniscal fibrocartilage regeneration, a subtotal meniscectomy (90% excision) ovine model was prepared. In the Blank group, only a subtotal meniscectomy model was prepared for the medial meniscus (**Figure** **7**A). In the Scaffold group, a biomimetic porous polyurethane (PU) meniscal scaffold was fabricated using a handheld bioprinter, implanted anatomically, and fixed with the peripheral residual meniscal tissue using sutures (Figure 7B). In the Scaffold+exercise group, regular exercise training was performed in the ovine in addition to scaffold implantation (Figure 7C; Movie S1, Supporting Information). Four months after the surgery, all of the samples were collected for evaluation. Only soft fibrous tissues were observed in the Blank group. Synovitis was apparent throughout the knee joint. Severe joint degeneration was observed, demonstrating morphological bone deformation, obvious articular cartilage erosion, and osteophyte formation, particularly in the medial femoral condyle (MFC) and medial tibial plateau (MTP) (Figure 7D). In the Scaffold group, the scaffold adhered to the peripheral meniscal tissue. However, the scaffold skeleton is irregular. Some fibrous tissue and a small amount of white fibrocartilage‐like tissue were observed in the scaffolds. However, more than 80% of the scaffold remained bare, with no repaired tissue infiltration. Synovitis was milder than that in the blank group. However, cartilage erosion and some osteophytes were still observed in the MFC and MTP (Figure 7E). In the Scaffold+exercise group, more fibrocartilage‐like tissue was observed within the scaffold. More than 60% of the scaffold region was covered with the newly formed tissue. The scaffold tightly adhered to the peripheral meniscal tissue. The scaffold skeletons remained intact and regular. Mild synovitis was observed in these joints. Mild cartilage abrasion was observed in the MFC and MTP (Figure 7F).

The samples were divided into the anterior horn, body, and posterior horn for histological analysis. In the Blank group, histomorphological analysis revealed that only a few fibrous and fibrovascular tissues adhered to the remaining peripheral meniscal tissue. There was no or weak Safranin O staining within the newly formed tissue, indicating poor GAG deposition. In the Scaffold group, histomorphological analysis demonstrated some fibrous and fibrovascular tissues and little fibrocartilaginous tissue within the scaffold. Most of the scaffolds were bare, with no regenerated tissue infiltration. The scaffold exhibited poor integration with the remaining peripheral meniscal tissue. Negative or weak intensity of safranin O staining was observed, demonstrating poor GAG deposition. In the Scaffold+exercise group, the histological analysis revealed the formation of more fibrocartilaginous tissue, demonstrating rich collagen and GAG matrix deposition. The newly formed tissue contains more oval‐shaped fibrochondrocyte‐like cells. As demonstrated by safranin O staining, the regenerated tissue was rich in GAG, especially in the inner and middle zones, similar to native menisci. The Scaffold+exercise group had the highest meniscal repair scores compared with the Blank or Scaffold groups (Figure 7G–K). Next, the mechanical properties, including elastic modulus and hardness, were evaluated using nanoindentation. The elastic modulus of the Scaffold+exercise group (2.46 ± 0.23 MPa) was superior to that of Blank group (0.43 ± 0.06 MPa) and Scaffold group (1.17 ± 0.11 MPa), but was still inferior to that of native meniscus (4.21 ± 0.97 MPa) significantly. The hardness of Scaffold+exercise group (0.3 ± 0.04 MPa) was superior to that of Blank group (0.1 ± 0.02 MPa) and Scaffold group (0.17 ± 0.02 MPa), but was still inferior to that of native meniscus (0.44 ± 0.07 MPa) significantly (Figure 7L).

**Figure 8:**
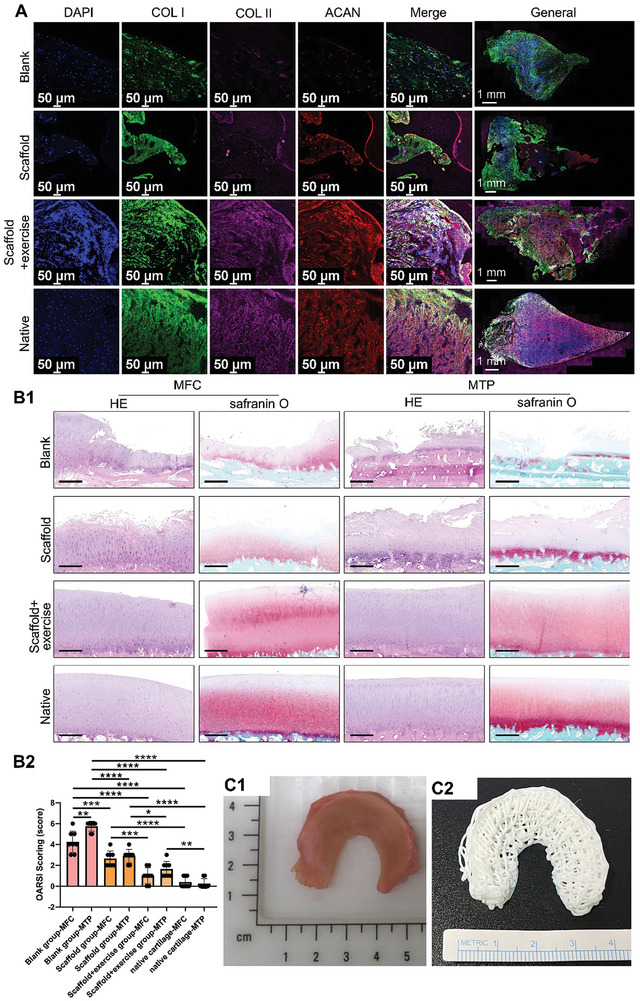
Histological analysis of regenerated menisci and knee joint cartilage degeneration. A) Representative immunofluorescence images of meniscus tissues in each group demonstrating the deposition of COL I, COL II, and ACAN. B) Evaluation of cartilage degeneration in MFC and MTP (B1, histological analysis of cartilage degeneration, scale bar: 0.5 mm; B2, OARSI scoring reflecting cartilage degeneration: a total of eight slices of MFC or MTP per group were evaluated (one‐way ANOVA)). C) Preparation of human meniscus scaffolds using a handheld bioprinter (C1, native human meniscus; C2, fabrication of porous meniscus scaffold using handheld bioprinter). COL I represents type I collagen, COL II represents type II collagen, ACAN represents aggrecan, MFC represents medial femoral condyle, MTP represents medial tibial plateau, * represents *p*<0.05, ** represents *p*<0.01, *** represents *p*<0.005, and **** represents *p*<0.001.

Collagen (COL I and COL II) and aggrecan matrix deposition within the regenerated tissue was evaluated using immunofluorescence. In the Scaffold+exercise group, the newly formed tissue was abundant in COL I, COL II, and aggrecan. The COL I matrix was distributed throughout the regenerated meniscal tissue but was more robust in the peripheral outer region. COL II matrix deposition was more robust in the inner and middle zones than in the peripheral outer zone, demonstrating an anisotropic distribution resembling that of native menisci. Similarly, the aggrecan matrix deposition was abundant and more robust in the inner and middle zones, displaying anisotropy. However, in the Blank or Scaffold groups, less COL I, COL II, and aggrecan matrix deposition was observed. In particular, the intensities of COL II and Aggrecan were lower than those in the remaining outer native meniscal tissue. Semiquantitative analyses showed that the Scaffold+exercise group had the highest contents of COL I, COL II, and aggrecan matrix compared to those of the Blank or Scaffold groups and was closer to native menisci (**Figure** **8**A; Figures S13–S19, Supporting Information). Within the regenerated tissue of the Blank, Scaffold, Scaffold+exercise groups, abundant vascular tissue was observed (Figures S20–S22, Supporting Information). Joint cartilage degeneration in the MFC and MTP was evaluated histologically. The Scaffold+exercise group demonstrated less cartilage erosion, decreased GAG contents, and lower OARSI cartilage degeneration scores than the Blank or Scaffold groups. However, severe cartilage abrasion and decreased GAG contents were observed in the blank group, especially in the MTP group. The Blank group exhibited the highest OARSI score (Figure 8B). Furthermore, a human meniscal porous scaffold was fabricated using a handheld bioprinter based on the native human meniscus, demonstrating its practicality and feasibility in a surgical environment (Figure 8C).

### 2.6 Single Cell RNA Sequencing (scRNA‐Seq) Analyses Reveals that EndMT Gives Rise to Fibrochondrocytes and Contributes to Meniscal Regeneration

**Figure 9:**
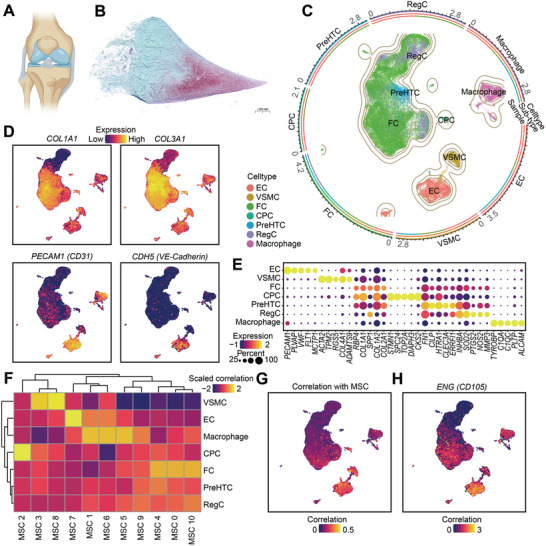
The scRNA‐seq analysis reveals mesenchymal signatures in the regenerated menisci. A) Schematic diagram of the knee joint. B) Histomorphology of a meniscal cross section. C) All cells from meniscus tissues colored based on the cell types in UMAP. The rounding circles showing the percentages of cell types, sub‐types, and samples. D) Expression of marker genes of FC (COL1A1 and COL3A1) and EC (CD31 and CDH5) presented in UMAP. E) Dot plot showing the expression of marker genes (column) of each cell type (row). F) Heatmap of correlation between the meniscal cell types (row) and mesenchymal stem cells (column). G) Maximum of correlation scores between all sub‐types of mesenchymal stem cells and meniscal cells presented as a UMAP plot of meniscal cells. H) Expression of the endothelial to mesenchymal transition marker gene ENG (CD105) in meniscal cells, presented as a UMAP plot. EC represents endothelial cell, VSMC represents vascular smooth muscle cell, FC represents fibrochondrocyte, CPC represents cartilage progenitor cell, PreHTC represents prehypertrophic chondrocyte, and RegC represents regulatory chondrocyte.

Single‐cell RNA sequencing (scRNA‐Seq) is a well‐established and powerful method for investigating transcriptomic cell‐to‐cell variations that can identify cell types and provide meaningful clues during tissue physiological, pathological, and regenerative processes.^[^ ^20^ ^]^ We used scRNA‐Seq to clarify the cell populations, gene expression profiles, and cell differentiation trajectories in regenerated and native menisci (**Figure** **9**A,B). In the present study, the regenerative (Scaffold+exercise group) and native ovine meniscus tissues were collected for scRNA‐Seq. To comprehensively evaluate the EndMT phenomenon in the menisci of different species, the regenerative and native meniscus tissues of beagle canines from our previous study (unpublished data) were also included and analyzed with scRNA‐Seq data of the ovine model. We identified seven major cell populations: fibrochondrocytes (FC, expressing *COL1A1, COL3A1*),^[^ ^21^ ^]^ ii. regulatory chondrocytes (RegCs, expressing *BMP2, FOSL1*)^[^ ^22^ ^]^; iii. Vascular smooth muscle cells (VSMC, expressing *MYH11*, *ACTA2*),^[^ ^23^ ^]^ iv. Endothelial cells (EC, expressing *CDH5, CD31*),^[^ ^24^ ^]^ v. Prehypertrophic chondrocytes (PreHTCs, expressing *CILP* and *CLEC3A*),^[^ ^25^ ^]^ vi. Cartilage progenitor cells (CPC, expressing *CDK1* and *SPC24*),^[^ ^26^ ^]^ vii, and macrophages (expressing *C1QA* and *C1QB*)^[^ ^27^ ^]^ (Figure 9C–E; Figure S23A–D, Supporting Information). The same cell types from different samples exhibited the same clustering in correlation analysis, demonstrating controlled batch effects. Next, to reveal the role of MCs in the meniscus, we performed correlation analyses on the scRNA‐Seq data of the meniscus and published the scRNA‐Seq data of mesenchymal stem cells (GSE182158).^[^ ^28^ ^]^ The published mesenchymal data revealed a total of 12 MSC subpopulations (MSC clusters 0–11) from the bone marrow, adipose tissue, umbilical cord, and dermal tissue. After correlation analysis, the ECs in the meniscus showed a high correlation with MSC cluster 7 and the FCs in the meniscus showed a high correlation with the MSC clusters 0, 4, and 10 (Figure 9F,B), demonstrating the mesenchymal phenotype of ECs and FCs in the meniscus. To further confirm the occurrence of EndMT in the meniscus, we analyzed the expression profiles of MSC‐ and EndMT‐associated genes. We detected gene expression of MSCs markers (*CD90, CD29*, and *CD73*)^[^ ^28^, ^29^ ^]^ and EndMT markers (*VIMENTIN, ACTA2, S100A4, COL1A2, FN1*, and *CD105*),^[^ ^30^ ^]^ which confirmed the phenomenon of EndMT in the meniscus (Figure 9H; Figure S23E, Supporting Information).

**Figure 10:**
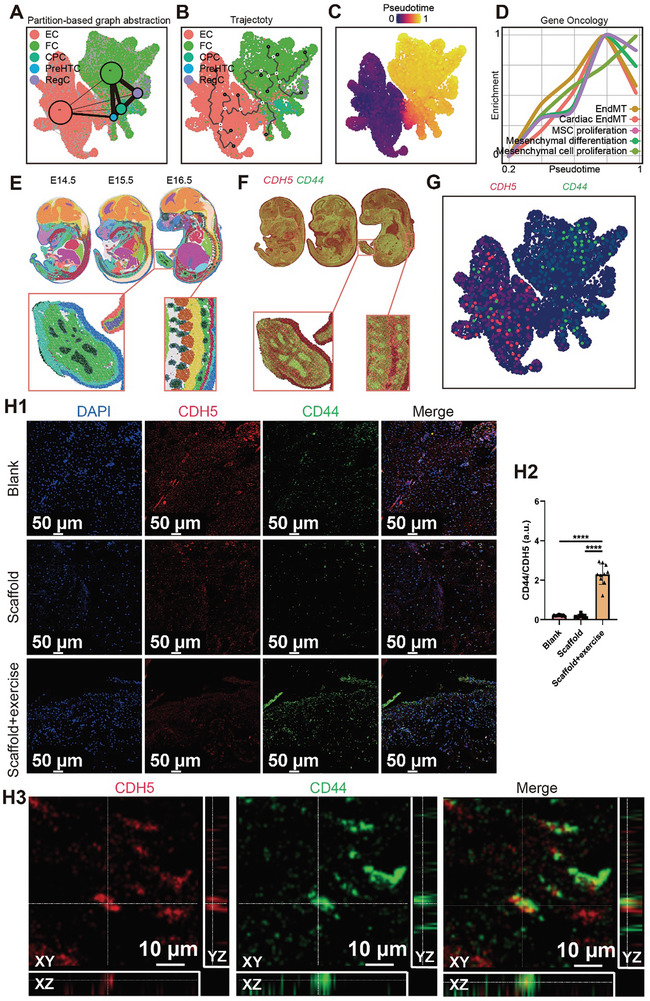
EndMT in the regenerated menisci. A) Partition‐based graph abstraction (PAGA) in the force‐directed graph (PAGA‐initialized single‐cell embeddings) predicting the developmental trajectory from EC to FC. B) The dots of the trajectory from EC to FC predicted by Monocle 3. C) Pseudotime predicted by Scanpy, Monocle3, and Slingshot presenting the gradual transition from EC to FC. D) Enrichment of gene oncology (GO) terms (including EndMT, cardiac EndMT, MSC proliferation, mesenchymal cell differentiation, and mesenchymal cell proliferation) along the pseudotime. E) Spatiotemporal transcriptomic atlas of mouse organogenesis including embryonic day (E) 14.5, 15.5, and 16.6 showing the organs. F) Spatiotemporal expression of *CDH5* (red) and *CD44* (green) during mouse organogenesis; the red box represents the foot and spine. G) Co‐expression of *CDH5* (red) and *CD44* (green) presented in the single‐cell UMAP of meniscal EC and FC. H) Immunofluorescent colocalization of CDH5 and CD44 within newly regenerated ovine tissues (H1, immunofluorescent staining; H2, semiquantitative analysis of the CD44 fluorescence intensity divided by the CDH5 fluorescence intensity in each group: a total of 10 slices per group were evaluated (one‐way ANOVA); H3, orthogonal projection of CDH5 and CD44 within the regenerated tissue of Scaffold+exercise group, which confirmed the colocalization of CDH5 and CD44 in 3D view). a.u. represents arbitrary unit and **** represents *p*<0.001.

To further evaluate the role of EndMT in the meniscus, we constructed a developmental trajectory between ECs and a subset of FCs showing a high correlation with MSCs (**Figure** **10**A–C; Figure S24A, Supporting Information). Pseudo‐trajectory analysis demonstrated that ECs gave rise to FCs. In addition, GO enrichment analysis confirmed the characteristics of developmental trajectories from ECs to FCs. At an early pseudotime, EC‐related GO, such as EC proliferation, EC migration, and EC development, was found to be highly enriched. In the middle pseudotime, EndMT and MC‐related GO terms, including MC proliferation, MSC proliferation, EndMT, and MSC differentiation, were enriched. At a later pseudotime, cartilage‐related GO terms, such as cartilage condensation, chondrocyte differentiation, and chondrocyte development, were enriched. Moreover, the KEGG analysis revealed the TGF‐β signaling pathway was highly enriched during EndMT, which was consistent with the aforementioned molecular mechanisms that mechanical stimulus facilitated EndMT through TGF‐β signaling pathway. Next, we evaluated the gene expression profiles of ECs, EndMTs, MSCs, and fibrochondrocytes during the pseudotime of the developmental trajectory. We found the gene expression profiles also conformed to the characteristics of developmental trajectories from ECs to FCs, demonstrating the downregulation of the EC‐associated gene *CDH5*, upregulation of MSC‐associated genes (*N‐cadherin* and *CD44*), upregulation of fibrochondrocyte‐associated genes (*COL1A1* and *COL2A1*) and upregulation of *TGF‐β2* (Figure 10D; Figure S24B and C, Supporting Information).

A previous study demonstrated the spatiotemporal transcriptomic atlas of mouse organogenesis (MOSTA) during embryogenesis using DNA nanoball‐patterned arrays.^[^ ^31^ ^]^ Using the MOSTA database (https://db.cngb.org/stomics/mosta/spatial/), we acquired the spatial gene expression profiles during organogenesis (Figure 10E). First, from the MOSTA database, *CDH5* was found to be partially co‐localized with *CD44* in cartilaginous and perichondral tissues during mouse metatarsal and vertebral development, which demonstrated the EndMT phenomenon (Figure 10F). In our scRNA‐Seq data of the meniscus, we confirmed the coexpression of *CDH5* and *CD44* in single‐cell UMAP cells (Figure 10G). We performed immunofluorescence co‐staining for CDH5 and CD44 in the regenerated tissue of the ovine model. Immunofluorescence results demonstrated the colocalization of CDH5 and CD44 in the newly formed tissue of the Blank, Scaffold or Scaffold+exercise groups, which further confirmed the phenomenon of EndMT during meniscal regeneration (Figure 10H1). The Scaffold+exercise group had the highest CD44/CDH5 value compared to that of the Blank or Scaffold groups, which reflected a more robust EndMT after the exercise stimulus (Figure 10H2). Moreover, the orthogonal projection of CDH5 and CD44 within the regenerated tissue further confirmed the colocalization of CDH5 and CD44 in 3D (Figure 10H3; Figure S25, Supporting Information). Second, from the MOSTA database, *CDH5* was found to be partially co‐localized with *N‐cadherin* in cartilaginous and perichondral tissues during mouse metatarsal and vertebral development, which demonstrated the EndMT phenomenon (Figure S26A, Supporting Information). Our scRNA‐Seq data of the meniscus confirmed the co‐expression of *CDH5* and *N‐cadherin* in single‐cell UMAP (Figure S26B, Supporting Information). We performed immunofluorescence co‐staining for CDH5 and N‐cadherin in the regenerated tissue of the ovine model. Immunofluorescence results demonstrated the colocalization of CDH5 and N‐cadherin in the newly formed tissue of the Blank, Scaffold or Scaffold+exercise groups, which further confirmed the phenomenon of EndMT during meniscal regeneration (Figure S26C1, Supporting Information). Moreover, the Scaffold+exercise group had the highest N‐cadherin /CDH5 value compared to that of the Blank or Scaffold group, which demonstrated more robust EndMT after the exercise stimulus (Figure S26C2, Supporting Information). Moreover, the orthogonal projection of CDH5 and N‐cadherin within the regenerated tissue further confirmed the co‐localization of CDH5 and N‐cadherin in 3D (Figures S26C3 and S27, Supporting Information). The alpha‐SMA was another mesenchymal marker. We performed immunofluorescence co‐staining for CDH5 and alpha‐SMA in the regenerated tissue of the ovine model. Immunofluorescence results demonstrated the co‐localization of CDH5 and alpha‐SMA in the newly formed tissue of the Blank, Scaffold or Scaffold+exercise groups, which further confirmed the occurrence of EndMT during ovine meniscal regeneration (Figures S28 and S29, Supporting Information). The orthogonal projection of CDH5, COL I, and COL II within the regenerated ovine tissue confirmed the colocalization of CDH5, COL I, and COL II (Figure S30, Supporting Information). In conclusion, ECs were transformed into fibrochondrocyte‐like cells during meniscal regeneration through EndMT. Third, from the MOSTA database, the *CDH5* was found to be partially colocalized expressed with *TGF‐β2* in the cartilaginous and perichondral tissue during mouse metatarsal and vertebral development (Figure S31A, Supporting Information). In our scRNA‐Seq data of meniscus, we confirmed the co‐expression of *CDH5* and *TGF‐β2* in the single‐cell UMAP (Figure S31B, Supporting Information). We further performed the immunofluorescent staining of TGF‐β2 in the regenerated tissue of ovine model. The immunofluorescence results demonstrated the regenerated tissue in the Scaffold+exercise group had the highest content of TGF‐β2 compared to that of Blank or Scaffold group, which demonstrated exercise stimulus activated TGF‐β2 signaling (Figure S31C, Supporting Information).

## 3 Discussion

EndMT is a phenotypic change that is associated with the transformation of ECs to MCs. During EndMT, ECs lose their endothelial phenotype defined by the expression of endothelial markers such as PECAM1 (CD31) or VE cadherin (CDH5) and instead exhibit a mesenchymal phenotype by expressing markers such as N‐cadherin and fibroblast‐specific protein 1 (FSP1).^[^ ^14a^ ^]^ Traditionally, EndMT has been considered a source of fibroblasts and myofibroblasts, and has been widely studied in physiological cardiac development, pathological tumors, and fibrosis.^[^ ^32^ ^]^ EndMT was originally identified as an important mechanism in heart development.^[^ ^33^ ^]^ Vascular ECs around the atriventricular canal and outflow tract undergo EndMT and invade the surrounding tissues to form heart valves and septa.^[^ ^34^ ^]^ EndMT plays an important role in cancer growth, angiogenesis, and metastasis. Cancer‐associated fibroblasts (CAFs) was key components of these processes. A previous study revealed up to 40% of CAFs were produced by EndMT, using *Tie2‐Cre* endothelial lineage reporter mice.^[^ ^35^ ^]^ Although EndMT is an embryonic process, it typically does not occur in adults. This phenomenon can be observed in adults under some conditions. A previous study demonstrated that fibroblasts in fibrotic lesions of cardiac fibrosis arise from EndMT using the *Tie1‐Cre* endothelial lineage reporter mice.^[^ ^36^ ^]^ Similarly, EndMT also produces myofibroblasts during renal fibrosis.^[^ ^37^ ^]^ Recent studies have found that EndMT‐transformed cells possess stem cell phenotypes, such as MSCs, which demonstrate that they can differentiate into chondrocytes, osteoblasts, adipocytes, and other cell types.^[^ ^32^, ^38^ ^]^ This phenomenon provides a new perspective for tissue repair and regeneration. EndMT during meniscus regeneration has not been reported previously. In the present study, we used *CDH5‐CreER^T2^; Rosa26‐LSL‐tdTomato* transgenic mice to trace ECs. The total meniscectomy model confirmed that EndMT occurred during meniscal regeneration, resulting in the formation of fibrochondrocytes.

Mechanical stimuli play a critical role in native meniscus development and maintenance of the meniscal cell phenotype. To investigate the critical role of mechanical stimuli in meniscal maturation and phenotype maintenance, a condylectomy model of the medial femoral condyle was established. Due to the small size of the mouse and rat models, we finally chose rabbits as an appropriate model. The present study demonstrated that the contents of meniscal fibrocartilaginous matrix components, including COL II and GAG, increased significantly with the maturity of the rabbit meniscus. However, without the mechanical stimulus of condyle compression, the native development of the juvenile rabbit meniscus was prominently affected, demonstrating abnormal meniscus morphology and less GAG and COL II deposition. In adult rabbit menisci, the absence of a mechanical stimulus resulted in the loss of GAG content and the meniscal cell COL II phenotype. Mechanical stimuli play a critical role in meniscal regeneration. Yan et al.^[^ ^10^ ^]^ developed an effective strategy to facilitate meniscus regeneration by 3D printing a biomimetic meniscal scaffold combining an autologous synovium transplant, which contained abundant MSCs. They concluded that the biomechanics upregulated Piezo1 expression, facilitating concerted activation of calcineurin and NFATc1, further activated the YAP‐pSmad2/3‐SOX9 axis, and consequently facilitating the fibrochondrogenesis of MSCs and meniscal regeneration. Balachandran et al. concluded that EndMT can be triggered by mechanical stimuli. When ECs were treated with 10% or 20% cyclic strain stimulus, the mesenchymal marker α‐SMA was upregulated, whereas the endothelial markers VE‐cadherin and CD31 were downregulated.^[^ ^39^ ^]^ In the present study, we observed that ECs transformed into MCs with a stem cell phenotype. Endothelial markers (CDH5 and CD31) were significantly downregulated, whereas mesenchymal markers (N‐cadherin, FSP‐1, and alpha‐SMA) and the MSC marker CD44 were significantly upregulated. Moreover, we demonstrated the mechanical stimulus facilitated EndMT via the TGF‐β2 signaling pathway. Therefore, initiating EndMT in migrated vascular ECs under a mechanical stimulus imposed by the knee joint during meniscal regeneration could be an efficient way to facilitate meniscal regeneration.

To further evaluate the role of EndMT in orthotopic and ectopic meniscus regeneration, a rat model was used. We first established a total meniscectomy model in rats and then injected EndMT‐transformed cells into the knee joint cavity. We found that transplanted EndMT‐transformed cells facilitated meniscal regeneration in rats. EndMT‐transformed cells showed robust expression of COL I and COL II, which are abundant in native meniscal fibrochondrocytes. The positive effects of MSCs injections on meniscal regeneration have been demonstrated in many previous studies.^[^ ^40^ ^]^ The injected MSCs directly differentiated into meniscal cells.^[^ ^40c^ ^]^ Thus, the transplanted EndMT‐transformed cells functioned like MSCs and differentiated into fibrochondrocyte‐like cells within the knee joint microenvironment. Second, an ectopic meniscal regeneration model was used. The EndMT‐transformed cells were seeded into the meniscus‐shaped porous scaffold and then subcutaneously implanted into the back of the rat. Standard chondrogenic medium was regularly injected into the scaffold to create a chondrogenic microenvironment. Interestingly, meniscal fibrocartilaginous tissue was acquired, demonstrating robust deposition of GAG, COL I, and COL II. EndMT‐transformed cells demonstrate robust expression of the chondrogenic transcriptional factor‐SOX9. Therefore, EndMT has a positive effect on meniscal regeneration and may be an innovative method to facilitate meniscal fibrocartilage regeneration.

Mechanical stimuli play a critical role in meniscal regeneration. We further clarified that regular exercise facilitated meniscal regeneration prominently after transplanting the biomimetic porous meniscus scaffold fabricated intraoperatively using a handheld bioprinter in an ovine subtotal meniscectomy model. The knee joint of the ovine model was closer in size to the human knee joint, which was more clinically relevant. Moreover, the use of ovine models has been increasing in meniscus repair and regeneration studies in recent years.^[^ ^41^ ^]^ The newly formed tissue contained a robust deposition of meniscal fibrocartilaginous tissues, such as GAG, COL I, COL II, and aggrecan. Moreover, the deposition of the fibrocartilage matrix was anisotropic, demonstrating COL I mainly in the peripheral outer zone and GAG, COL II, and aggrecan mainly in the inner zone, similar to the native meniscus. It has been demonstrated that GAG, COL II, and the aggrecan matrix can resist compression, whereas the COL I matrix mainly resisted tension.^[^ ^10^ ^]^ Thus, the anisotropy of the matrix distribution within the regenerated tissue allowed it to disperse mechanics during knee joint motion similar to native menisci, which was beneficial for maintaining the mechanical balance of the knee joint. However, the integration of the implanted scaffold with the surrounding native tissue and the subsequent vascularization process are crucial for successful meniscal regeneration. Polyurethane (PU) is a versatile and highly tunable class of materials with high tensile strength, abrasion and fatigue resistance, and flexibility. In addition, polyurethanes with excellent biocompatibility and hemocompatibility can be synthesized, enabling their use as biomaterials in the medical field.^[^ ^42^ ^]^ Moreover, the polyurethane material has been used to fabricate meniscal tissue engineering scaffolds and substitutes.^[^ ^43^ ^]^ PCL has been previously used in meniscal tissue engineering.^[^ ^7b^ ^]^ However, PCL is hard and brittle.^[^ ^43a^ ^]^ The hardness of PCL can cause secondary cartilage injuries. The meniscus is a weight‐bearing tissue that withstands joint compression throughout life. PCL is brittle and cannot endure intensive compression and deformation. Thus, in the present study, a biocompatible polyurethane material was used to fabricate a meniscus scaffold. Previous studies demonstrated that synovial tissue could migrate to the damaged meniscus.^[^ ^40^, ^44^ ^]^ Blood vessels are abundant in the synovial tissues. After meniscal scaffold transplantation, the synovial tissue within the knee joint can migrate and enwrap the scaffold, facilitating tissue ingrowth and vascularization. Moreover, the peripheral meniscal tissue is more vascularized than the inner avascular zone.^[^ ^4^ ^]^ Thus, ECs from the peripheral meniscal tissue can migrate into the scaffold, facilitating vascularization in the scaffold and tissue integration. Exercise is a strong driver of physiological angiogenesis.^[^ ^45^ ^]^ Thus, exercise‐induced angiogenesis within the meniscal scaffold is beneficial for effective nutrient exchange and subsequent tissue regeneration. In addition, joint fluid is rich in nutrients. Joint compression imposed by exercise can facilitate the penetration of joint fluid into the meniscus and cartilage tissue. This is another method that is used to accelerate nutrient exchange and tissue repair.

Interestingly, scRNA‐seq analysis demonstrated that ECs within the meniscus were highly correlated with MSCs, revealing the occurrence of EndMT. Immunofluorescence analysis of regenerated meniscal tissue demonstrated that the endothelial marker (CDH5) was colocalized with the mesenchymal markers N‐cadherin and alpha‐SMA and the MSC marker CD44 within the regenerative cells, which confirmed the EndMT phenomenon. Moreover, the newly formed tissue of the Scaffold+exercise group demonstrated more robust expression of N‐cadherin and CD44 and lower expression of CDH5 than the Blank and Scaffold groups, further verifying that the mechanical stimulus induced by exercise training facilitated EndMT during meniscal regeneration in vivo. Interestingly, from the spatiotemporal transcriptomic atlas of mouse organogenesis (MOSTA) database,^[^ ^31^ ^]^ *CDH5* was found to be partially co‐localized with *CD44* and *N‐cadherin* in cartilaginous and perichondral tissues during mouse metatarsal and vertebral development, which demonstrated the emergence of EndMT. Subsequently, developmental trajectory analysis demonstrated that the ECs gave rise to fibrochondrocytes in the menisci. The results of the GO enrichment analysis was consistent with the characteristics of the developmental trajectories from ECs to FCs. At an early pseudotime, EC‐related GO, such as EC proliferation, EC migration, and EC development, was highly enriched. In the middle pseudotime, EndMT and MC‐related GO terms, including MC proliferation, MSC proliferation, EndMT, and MSC differentiation, were enriched. At a later pseudotime, cartilage‐related GO was enriched, such as cartilage condensation, chondrocyte differentiation, and chondrocyte development. The gene expression profiles also conformed to the characteristics of the developmental trajectories from ECs to FCs, demonstrating the downregulation of the EC‐specific gene *CDH5*, upregulation of MSC‐specific genes (*N‐cadherin* and *CD44*), and upregulation of fibrochondrocyte‐specific genes (*COL1A1* and *COL2A1*). Moreover, we performed immunostaining to verify the colocalization of CDH5, COL I, and COL II within the regenerated tissue of the ovine model, which further confirmed that FCs originated from ECs. Endothelial lineage‐tracing mice further confirmed the EndMT phenomenon and gave rise to FCs during meniscal regeneration. End‐MT is stimulated by several factors. The TGF‐β/BMP family growth factors are the most common, especially, the TGF‐β2 isoform. Embryonic EndMT has been reported to be suppressed in TGF‐β2 gene knockout mice, while EndMT was not shown to be affected in TGF‐β1 or TGF‐β3 gene knockout mice during development.^[^ ^12^ ^]^ We revealed the mechanical stimulus facilitated EndMT through activating TGF‐β2 signaling in vitro. The KEGG analysis of the meniscus scRNA‐seq revealed the TGF‐β signaling pathway was highly enriched during EndMT. Moreover, the gene expression of *TGF‐β2* was upregulated continuously during the developmental trajectory from ECs to FCs in the meniscus. We further confirmed the expression of TGF‐β2 was more robust in the regenerated tissues from the Scaffold+exercise group than in the tissues from the Blank or Scaffold groups; this demonstrates the positive effects of mechanical stimulus on activating TGF‐β2 signaling during meniscal regeneration. Thereby, the exercise stimulus enhanced EndMT via TGF‐β2 signaling in vivo, followed by transformation into fibrochondrocytes.

This study has several advantages over traditional meniscus tissue‐engineering studies. First, the porous meniscus scaffold can provide support for the knee joint and adhesion of regenerative cells and tissue. Recently, meniscal tissue engineering based on 3D bioprinting technology for biomimetic meniscus scaffold fabrication has been used to regenerate native meniscus like tissue^[^ ^7^ ^]^ and became a promising method for regenerating menisci in the future. Recent studies usually acquired meniscus imaging data, such as CT or MRI, followed by the reconstruction of 3D printing model. A meniscus scaffold for implantation was fabricated preoperatively. However, the meniscus tissue was resected and trimmed based on intraoperative observation and clinical damage, which was inconsistent with the preoperative plan. Therefore, the prepared meniscus scaffold would not fit and may cause adverse effects such as scaffold fracture and cartilage damage. In the present study, we fabricated a porous meniscus scaffold of PU biomaterial based on the resected meniscus tissue using a handheld bioprinter intraoperatively and then implanted it into the meniscus defect anatomically after subtotal meniscectomy in an ovine model, which could match the original meniscus defect and articular cartilage, thus avoiding adverse effects to the maximum extent. Therefore, we developed a more efficient, simplified, and personalized method for the intraoperative fabrication of biomimetic porous meniscus scaffolds for meniscal regeneration. Second, previous studies transplanted autologous or allogeneic cells, such as MSCs, fibrochondrocytes, or chondrocytes to facilitate meniscal regeneration.^[^ ^8a^ ^]^ However, the following adverse events cannot be neglected: the tumorigenicity of MSCs and the immunological rejection of allogeneic cells.^[^ ^8b^ ^]^ In the present study, migrated vascular tissue within the scaffold was extremely abundant and was regarded as the cell source for meniscal regeneration. Thus, transplantation of exogenous cells was omitted. Third, biochemical factors such as growth factors^[^ ^7^ ^]^ and small‐molecule drugs^[^ ^46^ ^]^ have been used in previous studies to promote fibrochondrogenesis and matrix deposition. However, the metabolism, release model, dose, and immunogenicity could not be identified in vivo. The present study used exercise rehabilitation to provide physical stimuli. The mechanical stimulus facilitates EndMT of the infiltrated vascular tissue during meniscal regeneration, avoiding the use of exogenous biochemical factors. Given these aforementioned advantages, the meniscal regeneration strategy used in this study is more translational and practical in clinical settings. After careful evaluation of the clinical meniscus injury cases, the porous meniscus scaffold was fabricated intraoperatively according to the resected meniscus tissue using a handheld bioprinter and then fixed into the meniscus defect anatomically. Weight‐bearing exercises were initiated after regular postoperative rehabilitation in the early phase. Moreover, a second‐look arthroscopic examination could be performed to trace meniscus regeneration and cartilage status. Optimal parameters for rehabilitation and exercise should be investigated in future clinical practice. Therefore, we developed an effective, simple, and translational strategy to facilitate meniscal regeneration. However, in certain clinical scenarios, the applicability of handheld bioprinters may be limited. For complex meniscal tears, the resected meniscal tissue cannot maintain integral morphology, which affects the subsequent printing path and shape design. This may cause a mismatch between the scaffold and condyle cartilage. Moreover, patients with extensive meniscus and cartilage degeneration are unsuitable. The limited intrinsic healing capacity of these patients affected meniscus regeneration.

The present study has some limitations. EndMT induction during meniscal regeneration contains potential variability, including EndMT degree and cell fate. Thus, further research is warranted to develop a precise EndMT induction drug system restricted to the meniscus tissue. Owing to the limited sample size of the ovine model, single‐cell RNA sequencing covered limited regenerative and native meniscus tissues. A loss‐of‐function approach to prove the functional involvement of EndMT program activation was missing in the ovine model. This study only evaluated meniscal regeneration at four months postoperatively, and a future long‐term follow‐up study is necessary to assess the durability and functional outcomes of the regenerated tissue over time.

In conclusion, the present study demonstrates that EndMT contributes to meniscal regeneration. We developed a handheld bioprinter system to intraoperatively fabricate a porous meniscus scaffold according to the resected meniscus tissue, which could match the original meniscus defect and articular cartilage morphology, thereby simplifying the fabrication procedures and period. Moreover, transplantation of a porous meniscus scaffold combined with a postoperative regular exercise stimulus facilitated the regeneration of anisotropic fibrocartilaginous tissue and protected the joint cartilage from degeneration in an ovine subtotal meniscectomy model. Mechanistically, the mechanical stimulus facilitated endothelial mesenchymal transition through activating TGF‐β2 signaling. EndMT‐transformed cells give rise to fibrochondrocytes and subsequently contribute to meniscal fibrocartilage regeneration. Therefore, we developed an effective translational strategy for facilitating meniscal regeneration. A schematic diagram of the mechanical stimulus facilitating EndMT, and the consequent meniscal regeneration is illustrated in Figure S32 (Supporting Information).

## 4 Experimental Section

### Ethics Statement

All surgical procedures and postoperative care of animals were performed according to the guidelines of the Institutional Animal Care and Use Committee. This animal study was approved by the Animal Ethics Committee of Peking University (LA2021007).

### Mice Model

To confirm EndMT during meniscal regeneration, *CDH5‐CreER^T2^; Rosa26‐LSL‐ tdTomato* endothelial lineage‐tracing transgenic mice were used to trace ECs.^[^ ^15^ ^]^ *CDH5‐CreER^T2^; Rosa26‐LSL‐tdTomato* transgenic mice were generated using C57BL/6Smoc‐*Cdh5^em1(2A‐CreERT2‐WPRE‐polyA)Smoc^* and C57BL/6JSmoc‐Gt(ROSA)26Sor*^em(CAG‐LSL‐tdTomato)1Smoc^* mice. Cre‐ERT2 mice express a fusion protein of a ligand‐binding region mutant (ERT) containing the estrogen receptor (ER) and Cre recombinase. Cre‐ERT2 remains inactive in the cytoplasm without tamoxifen induction. After Tamoxifen induction, the tamoxifen metabolite, 4‐OHT (an estrogen analog), binds to ERT, allowing Cre‐ERT2 to enter the nucleus and exert Cre recombinase activity. The tdTomato protein was expressed under active Cre recombinase conditions. In the present study, *CDH5* positive cells expressed tdTomato protein (a red fluorescent protein, RFP) after tamoxifen induction. Four transgenic male mice (8 weeks) were used in this study. The tamoxifen was dissolved in corn oil to achieve a concentration of 20 mg/ml by shaking at 37 °C. A standard dose of 100 ul tamoxifen/corn oil solution was administered via intraperitoneal injection once a day for a total of 7 consecutive days. A total meniscectomy model of the medial menisci in bilateral knees was established 14 days after tamoxifen induction. The meniscectomy process was performed using a stereomicroscope. The knee joints were collected 5 weeks after surgery for histological analysis.

### Rabbit Model

To study the maturity of the rabbit meniscus, meniscal tissue was collected at different developmental stages (male, postnatal 2 days, 9 days, 2 months, and 6 months). Hematoxylin and eosin (HE) staining was performed for histomorphological evaluation. Safranin O (SO) staining was used for GAG evaluation. Immunostaining was used for COL II.

To investigate the critical role of mechanical stimuli in meniscal maturation and phenotype maintenance, the condylectomy model of the MFC was established in four juvenile rabbits (male, 2 weeks after birth) and four adult rabbits (male, 6 months after birth). The condylectomy model was created in the left knee, while the right knees served as controls. Medial meniscal samples were collected 6 weeks after surgery, including macroscopic evaluation and histological analyses, such as HE staining for histomorphological evaluation, SO staining, toluidine blue staining for GAG evaluation, and immunostaining for COL II.

### Rat Model

First, the role of EndMT‐transformed cell transplantation in meniscal fibrocartilage regeneration using an orthotopic meniscus defect rat model was investigated. A total of 9 rats (female, 2 months old) were used in this study. A total meniscectomy model of the medial menisci was created in the bilateral knees of nine rats. The meniscectomy process was performed using a stereomicroscope. Blank, EC, and EndMT groups were included. Each group included three rats (six knees). In the present study, ECs from male rabbits were used to trace the fate of transplanted cells. In situ hybridization of male‐specific sex‐determining region Y‐linked genes in male rabbits was used to distinguish the transplanted cells. The ECs were treated with 10 ng ml^−1^ TGF‐β2 for 4 days to induce EndMT transformation. In the Blank group, only the medial meniscus was resected. In the EC group, ECs were injected into the knee joint 3 days after surgery. In the EndMT group, EndMT‐transformed cells were injected into the knee joint 3 days after surgery. Eight weeks after cell injection, meniscal regeneration was evaluated by histological analysis. The meniscal repair scoring system described by Zellner et al.^[^ ^47^ ^]^ was used for semiquantitative evaluation of meniscal healing. The meniscal repair scoring system was summarized in Table S2 (Supporting Information).

Subsequently, an ectopic meniscal regeneration model was established in the back of the rat. A semilunar meniscus‐like porous scaffold of the PU biomaterial was prepared using 3D printing. A total of 24 rats were included. The Scaffold+gel‐MA, Scaffold+gel‐MA+EC, Scaffold+gel‐MA+EndMT, Scaffold+gel‐MA+EndMT+Chondrogenic Medium (CM) groups were included. Two meniscal scaffolds were implanted subcutaneously in the back of each rat. Each group consisted of 6 rats (12 samples). ECs were harvested from the male rabbits. The ECs were treated with 10 ng ml^−1^ TGF‐β2 for 4 days to induce EndMT transformation. In the Scaffold+gel‐MA group, ≈200 µl of gel‐MA solution was injected into the scaffold and then cured with blue light. The prepared scaffolds were implanted subcutaneously into the backs of rats. In the Scaffold+gel‐MA+EC group, ≈200 µl of gel‐MA solution containing 1×10^6^ ECs were injected into the scaffold followed by crosslink with blue light and then implanted to the rat back subcutaneously. In the Scaffold+gel‐MA+EndMT group, ≈200 µl of gel‐MA solution containing 1×10^6^ EndMT transformed cells were injected into the scaffold followed by crosslink with blue light and then implanted to the rat back subcutaneously. In the Scaffold+gel‐MA+EndMT+CM group, ≈200 µl of gel‐MA solution containing 1×10^6^ EndMT‐transformed cells were injected into the scaffold followed by crosslink with blue light and then implanted to the rat back subcutaneously. To induce the chondrogenic differentiation of EndMT‐transformed cells, a chondrogenic medium was used. At 3 days after surgery, 100 µl CM was injected to the scaffold through subcutaneous injection every 3 days. To ensure even distribution of the CM, the contour of the scaffold was first identified by touching, and then the medium was injected throughout the entire scaffold. The CM contained basic growth medium (α‐MEM) supplemented with 10 ng ml^−1^ TGF‐β1 (100‐21, Peprotech, USA), 10 nM dexamethasone (Sigma–Aldrich, USA), 50 ug ml^−1^ ascorbate‐2‐phosphate (Sigma‐Aldrich, USA), 6.25 ug ml^−1^ insulin‐transferrin‐selenium (ITS, Gibco, USA). The control group was injected with α‐MEM only. Eight weeks after the injection, all samples were collected for histological analysis to evaluate fibrocartilage regeneration. Toluidine blue staining was used to evaluate GAG content. The deposition of COL I and COL II was evaluated using immunostaining. ISH co‐staining for the SRY gene and SOX9 immunofluorescence was used in order to evaluate the chondrogenic differentiation of transplanted cells.

### Endothelial Cell Isolation and Expansion

First, ECs were harvested from male rabbits. The thoracic and abdominal aortas were harvested. The aorta was rinsed with cold sterile PBS to remove blood clots. The peripheral soft tissue was removed using scissors. The intimal layer was separated using a scalpel. Then, the intima layer was cut into ≈0.1‐mm pieces and then digested with 0.2% type I collagenase (Gibco, USA) for 2 h at 37 °C. The resultant mixture was filtered using a 70‐µm nylon filter to remove undigested debris. Cells were washed with PBS and centrifuged twice and then resuspended with α‐MEM containing 10% fetal bovine serum (FBS), 100 U mL^−1^ penicillin, and 100 mg/mL streptomycin (Invitrogen, USA). The resuspended cells were cultured in Ф 10 cm culture dishes. The cells were allowed to attach for 2 days, and non‐adherent cells were removed by changing the medium every 2 days. After 14 days, the cells were digested with trypsin–EDTA (0.25% trypsin, 1 mM EDTA; HyClone, Logan, UT, USA) and regarded as passage 0 (P0). Then, P0 ECs were passaged at a ratio of 1:3. Subsequent passages were performed when the cells reached 80%–90%. Passage two (P2) ECs were used in subsequent experiments.

### Cell Treatment

Human umbilical vein endothelial cells (HUVECs) were used to investigate the effects of biomechanical stimuli on EndMT and its potential mechanisms. Cyclic tensile strain (CTS) was used in vitro to simulate biomechanical stimuli on HUVECs. A previous study demonstrated that native menisci have a mean strain of 5% under 100% weight‐bearing conditions.^[^ ^16^ ^]^ Thus, a periodic 5% strain was applied to the HUVECs for 12 h using an in vitro Flexcell train culture system.^[^ ^10^ ^]^ The mRNA and protein levels of endothelial and MCs were evaluated. RNA‐Seq analyses were performed on HUVECs after CTS or static treatment. Further, to evaluate the effect of TGF‐β2 on EndMT, the HUVECs were treated with 10 ng ml^−1^ TGF‐β2 for 4 days. The mRNA and protein levels in endothelial and MCs were evaluated. Further, to induce the EndMT transformation of rabbit ECs, the ECs were treated with 10 ng ml^−1^ TGF‐β2 for 4 days.

### Ovine Model

Sixteen adult sheep (male, 1 year, 40±5 kg) were included. Details of each sample are presented in Table S3 (Supporting Information). Meniscectomy (Blank), PU scaffold (scaffold), PU scaffold combined with regular exercise stimulus (Scaffold+exercise), and sham (native) groups were included. Standard anesthesia, skin preparation, disinfection, and surgical approaches were performed as described in previous studies.^[^ ^48^ ^]^ A standard medial parapatellar approach was used to expose the joint cavity and the patella was laterally dislocated. To expose the medial meniscus fully, the rectangular bone block of the superior attachment of the medial collateral ligament (MCL) in the MFC was removed using an oscillating saw. The tibia was externally rotated to expose the medial meniscus. In the control group, a total of 90% the meniscal tissue in the inner zone was resected using a scalpel. The MCL bone block was anatomically fixed using nails. In the Scaffold group, after performing subtotal meniscectomy, the contour of the resected meniscal tissue was drawn using a marker pen. The morphology of the scaffold is referred to as a contour. A polyurethane (PU) wire (1.75 mm of diameter, 85A; shore hardness) was inserted into the feed opening. The heat temperature was set as 200 °C. The wire can be driven forward by gears. Then, the PU lines of 700 µm could be extruded. The PU line was deposited layer‐by‐layer within the contour. The PU line was arranged biomimetically to simulate collagen fiber arrangement in the native meniscus, demonstrating circumferential and radial orientations. The interval between the PU lines was ≈700 m. The detailed process of scaffold fabrication is presented in Movie S2 (Supporting Information). The fabrication time for one ovine scaffold was ≈50 s in the present study. If the meniscus resection process and printing material preparation were included, the entire operation could be completed within 5 min. PU was used as the polymer. PU exhibits excellent mechanical properties and resistance to wear, oil, corrosion, oxidation, and UV radiation. It was a well‐known biomaterial used to fabricate meniscus scaffolds, implants, and many other medical devices.^[^ ^49^ ^]^ The thermoplastic PU used in this study was not biodegradable. Thus, the PU meniscus scaffold could provide persistent support for knee joint and tissue regeneration. In addition, a previous study demonstrated that small fragments and molecules from degradable materials can cause inflammation and other side effects.^[^ ^50^ ^]^ However, PU does not cause adverse effects owing to its non‐degradable properties. The scaffold was cooled naturally to room temperature for several minutes. The porous scaffold was then implanted anatomically and fixed with the peripheral meniscal tissue using sutures (2‐0 Ethibond). The MCL bone block was anatomically fixed using nails. In the Scaffold+exercise group, the preparation for subtotal meniscectomy and scaffold implantation were identical to those mentioned above. Regular exercise training (20 min per day, speed of 1.5 m ^−1^s) on a treadmill was started 2 weeks after surgery. In the native group, meniscectomy was not performed, and the remaining procedures were identical to those in the blank group. Nonsteroidal anti‐inflammatory drugs and penicillin were injected intramuscularly for two weeks to alleviate pain and infection.

All of the samples were collected four months postoperatively. The chemical components of the regenerated tissues were evaluated by histology and immunostaining. The mechanical properties of the regenerated tissues were evaluated using nanoindentation tests. A scRNA‐Seq analysis was performed to investigate the repair processes. The osteoarthritis cartilage histopathology assessment system (OARSI system) scoring^[^ ^51^ ^]^ was used to evaluate cartilage degeneration.

### Western Blotting

Proteins were extracted from HUVECs using RIPA lysis buffer (C1053, Applygen, China), which contained protease inhibitor (P1265‐1, Applygen, China) and phosphatase inhibitors (P1260‐1, Applygen, China). The protein concentration was measured using a NanoDrop spectrophotometer (Thermo Fisher Scientific, USA) at 280 nm ultraviolet. The proteins were separated using 4%–20% Bis‐Tris polyacrylamide gel (M00930, Genscript, China) electrophoresis and then transferred onto polyvinylidene fluoride membranes (PVDF) (ISEQ00010, Millipore, USA) according to standard procedures. The PVDF membranes were blocked with 5% (w/v) bovine serum albumin (BSA) (P1621, Applygen, China) for 1 h at room temperature. The membranes were incubated with primary antibodies overnight at 4 °C. After washing with TBST, membranes were incubated with secondary antibodies for 1 h at room temperature. After thorough washing with TBST, an ECL ultrawestern HRP (horseradish peroxidase) substrate was used to develop the band. Finally, the signals were captured using a ChemiDocXRS+Imaging System (Tanon, Shanghai, China). The relative intensity of the bands was analyzed using ImageJ software (National Institutes of Health, USA). The intensity of a specific protein divided by that of GAPDH represented its relative expression level.

### Quantitative Real‐Time Polymerase Chain Reaction (qPCR)

Total RNA was extracted using TRIzol reagent (15 596 018, Invitrogen, USA), according to the manufacturer's instructions. The RNA concentration was measured using a NanoDrop spectrophotometer (Thermo Fisher Scientific, USA). Reverse transcription was performed using a commercial kit (R323‐01, Vazyme, Nanjing, China). The qPCR was performed by magnifying the diluted complementary DNA of 20 µL with SYBR Green Q‐PCR Kit (Q141‐03, Vazyme, Nanjing, China) using the Applied Biosystems StepOnePlus Real‐Time PCR System (Foster City, CA, USA). The gene expression was calculated using the 2^−ΔΔCT^. The primer sequences for HUVECs are summarized in Table S4 (Supporting Information).

### Tissue Immunofluorescence, Immunohistochemistry, Semiquantitative Analysis

For tissue immunofluorescence, first, 3 µm‐thick paraffin sections were prepared using microtome (Leica, Germany). Sections were immersed in xylene and graded in ethanol to deparaffinize and regain water. Heat‐induced antigen retrieval was performed using citric acid (pH 6.0) for 20 min. Non‐specific protein binding was blocked using goat serum (Boster, AR0009, China) for 1 h at room temperature. The sections were then incubated with the corresponding primary antibodies for 2 h at room temperature. After thoroughly washing with PBST, sections were incubated with the corresponding secondary antibodies for 1 h at room temperature, followed by incubation with DAPI. Finally, the slices were sealed with an antifluorescence quenching agent. A confocal microscope (Leica, Germany) was used to capture images. For semi‐quantitative analysis, the integrated intensity of the corresponding target in the region of interest (ROI) was evaluated using the ImageJ software (National Institutes of Health, USA). The tissue slice of *CDH5‐CreER^T2^; Rosa26‐LSL‐tdTomato* endothelial lineage tracing transgenic mice knee incubated with only secondary antibodies (Donkey anti‐rat IgG H&L‐Alexa Fluor488, Donkey anti‐mouse IgG H&L‐Alexa Fluor594, Donkey anti‐goat IgG H&L‐Alexa Fluor647) was used as negative control (Figure S33, Supporting Information).

For tissue immunohistochemistry, the procedures prior to secondary antibody incubation were identical to those used for tissue immunofluorescence. Horseradish peroxidase‐conjugated secondary antibodies were then used. After thoroughly washing with PBST, the color was developed using a diaminobenzidine (DAB) substrate kit. Finally, slices were scanned using a digital scanner (NanoZoomer, Hamamatsu, Japan). For semi‐quantitative analysis, the integrated intensity of the corresponding target in the ROI was evaluated using the ImageJ software (US National Institutes of Health, USA).

### Co‐Staining of SOX9 Immunofluorescence and Biotin‐Labeled In Situ Hybridization (ISH) for SRY Gene

The SRY probe was designed according to a previous study.^[^ ^11^ ^]^ The probe sequence was as follows: SRY probe (5′ and 3′ biotin) TGCAAGCAGCAAACTGTCGCT (Qiagen, Germany). All solutions used for ISH were free of ribonucleases by high‐temperature sterilization. The paraffin‐embedded sections were immersed in xylene and ethanol to deparaffinize and regain water. The sections were permeabilized by 20 ug ml^−1^ proteinase K solution at 37 °C for 15 min and then washed with PBS. Heat‐induced antigen retrieval was performed using citric acid (pH 6.0) for 20 min. Then, the slices were treated with prehybridization buffer for 1 h at 37 °C. The tissue section was incubated with hybridization mix (hybridization buffer with SRY probe, 80 nM) for 1 h at 54 °C. After hybridization, a rigorous wash was performed using graded sodium citrate buffer (SSC) and PBS. Nonspecific protein binding was blocked with 1% bovine serum albumin (BSA) at room temperature for 15 min. The slices were then incubated with fluorescein isothiocyanate‐labeled anti‐biotin and SOX9 antibody for 1 h at room temperature. After washing with PBST, the sections were incubated with the secondary antibody and DAPI for 30 min. After thoroughly washing with PBST, the slices were sealed with an anti‐fluorescence quenching agent. A confocal microscope (TCS‐SP8; Leica) was used to capture images. The key reagents, software, and transgenic mouse models were summarized in Table S5 (Supporting Information).

### Nanoindentation Test

Nanoindentation was performed as described previously.^[^ ^48a^ ^]^ Briefly, the cross section of meniscus sample (500 µm in thickness) was prepared and then glued to a glass slide with surface vertical to the indentation direction. A rubber ring was glued to a glass slide to enclose the sample. PBS solution was added to keep the samples fully hydrated. Attention should be paid to avoid sample drying or dehydration during the testing process. A Tribo‐Indenter (Hysitron) with a 400 µm radius curvature, conospherical, diamond probe tip was used. A trapezoidal load function was applied by loading (10 s), holding (2 s), and unloading (10 s). The elastic modulus and hardness were obtained from the strain–stress curve.

### RNA Sequencing (RNA‐seq) of HUVECs

RNA‐seq analyses of HUVECs after CTS treatment were performed using the Dr. TOM platform (https://biosys.bgi.com). Total RNA was extracted using the TRIzol reagent. cDNA libraries were constructed for each pooled RNA sample using the MGIEasy RNA Directional Kit for total RNA‐Seq. Bowtie2(v2.2.5) was used to align clean reads to the gene set. Gene expression levels were calculated using RSEM (v1.3.1). Gene expression was determined by the TPM method. The differentially expressed genes were identified using the DESeq2(v1.4.5) algorithm. Significance analysis was performed using *P*‐value and false discovery rate (FDR) analyses. Genes with a fold change >2 or <0.5, FDR < 0.05, were considered to be differentially expressed. To gain insight into the phenotypic changes, GO (http://www.geneontology.org/) and KEGG (https://www.kegg.jp/) enrichment analyses of annotated expression gene were performed using Phyper (https://en.wikipedia.org/wiki/Hypergeometric_distribution) based on the hypergeometric test. Significantly affected GO categories and pathways were identified using Fisher's exact test. The *P* value was used to define the threshold of significance.

### Single Cell RNA‐Sequencing (scRNA‐Seq) of Meniscus Tissue

In the present study, the regenerative (Scaffold+exercise group) and native ovine meniscus tissues were collected for scRNA‐Seq. To comprehensively evaluate the EndMT phenomenon in the menisci of different species, the regenerative and native meniscus tissues of beagle canines from our previous study (unpublished data) were also included and analyzed with scRNA‐Seq data of the ovine model. Sequencing data were processed using CellRanger v7.1.0 with the reference genome *Ovis aries* (sheep) v3.1, to generate filtered expression matrices, which were analyzed using Seurat v 4.3.0.1.^[^ ^52^ ^]^

After normalizing the data, 5000 highly variable features for downstream analysis was used. The UMAP analysis was performed using the top 50 significant principal components. The cell type identities were migrated from scRNA‐Seq data of beagle canine via canonical correlation analysis by “TransferData” function of Seurat package. To validate the cell type identities, both classical markers and the “signature enrichment” analysis were adopted.^[^ ^53^ ^]^ The correlation between meniscal cells and MSCs was analyzed. The correlation of all meniscal cells grouped based on both the samples and cell types was calculated utilizing “cor” function of stats package in R v 4.3.1, as same as the correlation between our meniscal cell types and published scRNA‐seq data of MSCs.^[^ ^28^ ^]^ For the convenience of visualization, we maintained the maximum correlation between the MSC subtypes and meniscal cells. The ECs and FCs with maximum correlation values (specific for correlation with MSCs) higher than 0.11 were extracted for developmental trajectory construction. First, we built the single‐cell embeddings initialized by the partition‐based graph abstraction (PAGA) and the pseudotime of Scanpy was speculated.^[^ ^54^ ^]^ Second, Monocle was applied to the top 30 significant principal components to predict the pseudotime.^[^ ^55^ ^]^ Another pseudo‐time was proposed by Slingshot.^[^ ^56^ ^]^ These three calculated pseudotimes were averaged as the final pseudotimes from ECs to FCs. The average expression of all related genes from the GO terms, including EndMT, cardiac EndMT, MSC proliferation, MC differentiation, and MC proliferation, and from the KEGG term TGF‐β signaling pathway were calculated along the pseudotime via “AddModuleScore” function of Seurat package.^[^ ^53^, ^57^ ^]^

### Statistical Analysis

All data are presented as the means ± standard deviations (SDs). Data distribution was evaluated using the Shapiro‐Wilk test. Data were checked for equal variance before analysis. For the comparison of data from two groups, the two‐tailed Student's *t*‐test was performed. For the comparison of data from multiple groups, ordinary one‐way ANOVA or two‐way ANOVA with the Bonferroni multiple comparison test was applied. Differences with *P* values<0.05 were considered statistically significant. All statistical analyses were performed using GraphPad Prism software, version 8.0.1 (GraphPad Software, USA).

## Conflict of Interest

The authors declare no conflict of interest.

## Author's Contributions

W.Y. and H.W. contributed equally to this work. W.Q.Y. conducted the majority of the experiments, data collection, data analyses and manuscript preparation. H.D.W. conducted the data collection and analyses of single cell RNA sequencing and manuscript preparation. Y.W., Z.Y.G. conducted the preparation of animal models, data collection and manuscript preparation. Z.L., F.Y.Z., C.X.C. conducted some of the surgery, data collection and analysis, and manuscript preparation. Z.L., F.Y.Z. conducted some of cells related experiments, histological analysis. Y.F.A., X.Q.H., J.C. and J.Q.W. designed and supervised the whole project and reviewed the manuscript.

## Supporting information



Supporting Information

Supplemental Movie 1

Supplemental Movie 2

Supporting Tables

## Data Availability

The data that support the findings of this study are available from the corresponding author upon reasonable request.
