# Supplementary material for: Exercise Induced Endothelial Mesenchymal Transition (EndMT) Facilitates Meniscal Fibrocartilage Regeneration
Source: Adv Sci (Weinh). 2024 Sep 30;11(44):2403788. doi: 10.1002/advs.202403788 (PMC11600215; doi:10.1002/advs.202403788)
Supplement: Supplementary file 1 — Supporting Information [file ADVS-11-2403788-s004.pdf]

ADVANCED SCIENCE
Open Access

Supporting Information

for *Adv. Sci.*, DOI 10.1002/advs.202403788

Exercise Induced Endothelial Mesenchymal Transition (EndMT) Facilitates Meniscal Fibrocartilage Regeneration

Wenqiang Yan, Haoda Wu, Yue Wu, Zeyuan Gao, Zong Li, Fengyuan Zhao, Chenxi Cao, Jianquan Wang, Jin Cheng*, Xiaoqing Hu* and Yingfang Ao**

Supplemental information

Exercise Induced Endothelial Mesenchymal Transition (EndMT) Facilitates Meniscal Fibrocartilage Regeneration

Wenqiang Yan [1,2,3,#], Haoda Wu [1,2,3,#], Yue Wu [1,2,3], Zeyuan Gao [1,2,3], Zong Li [1,2,3], Fengyuan Zhao [1,2,3], Chenxi Cao [1,2,3], Jianquan Wang [1,2,3*], Jin Cheng [1,2,3*], Xiaoqing Hu [1,2,3,*], Yingfang Ao [1,2,3,*]

[1] Department of Sports Medicine, Peking University Third Hospital, Institute of Sports Medicine of Peking University, Beijing, China

[2] Beijing Key Laboratory of Sports Injuries, Beijing, China

[3] Engineering Research Center of Sports Trauma Treatment Technology and Devices, Ministry of Education, Beijing, China

Wenqiang Yan[#], Haoda Wu[#] contributed equally.

[*]corresponding author: Yingfang Ao, Xiaoqing Hu, Jin Cheng, Jianquan Wang

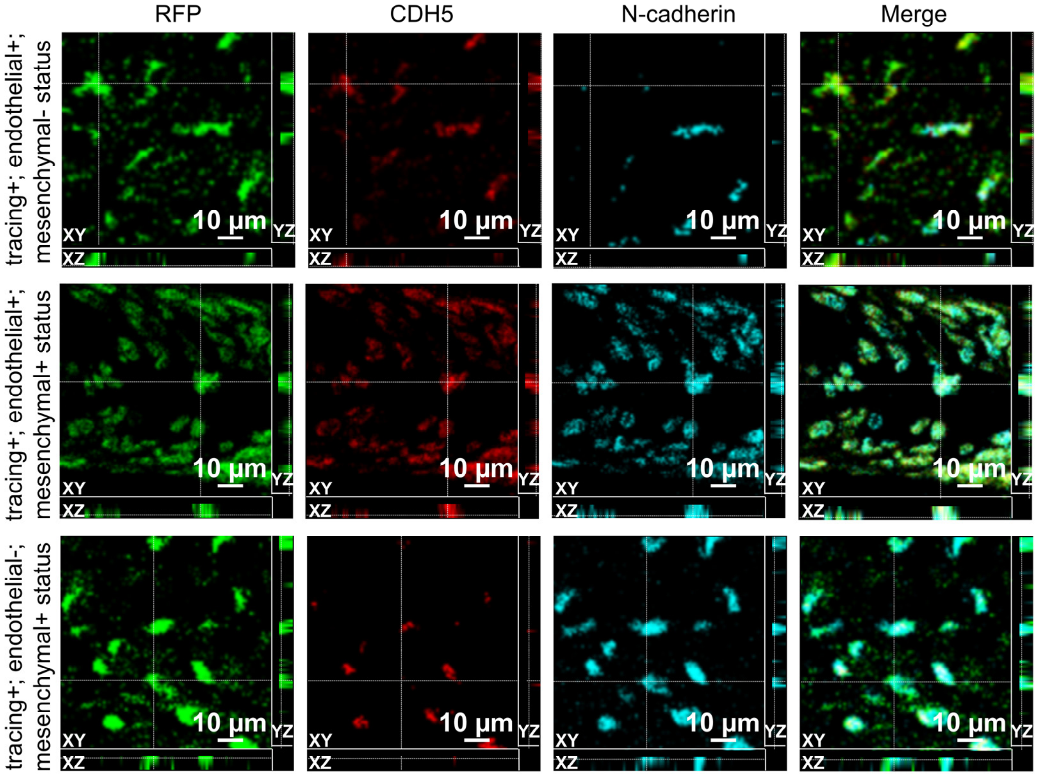

Supplemental Figure 1. The co-staining of RFP, CDH5, ad N-cadherin within the regenerated tissue of *CDH5-CreER^{T2}; Rosa26-LSL-Tdtomato* endothelial lineage tracing transgenic mice. RFP represents the lineage tracing status. CDH5 represents the endothelial status. N-cadherin represents the mesenchymal status. The tracing+; endothelial+; mesenchymal- status represents there is no EndMT within these cells. The tracing+; endothelial+; mesenchymal+ status represents there is partial EndMT within these cells. The tracing+; endothelial-; mesenchymal+ status represents there is full EndMT within these cells.

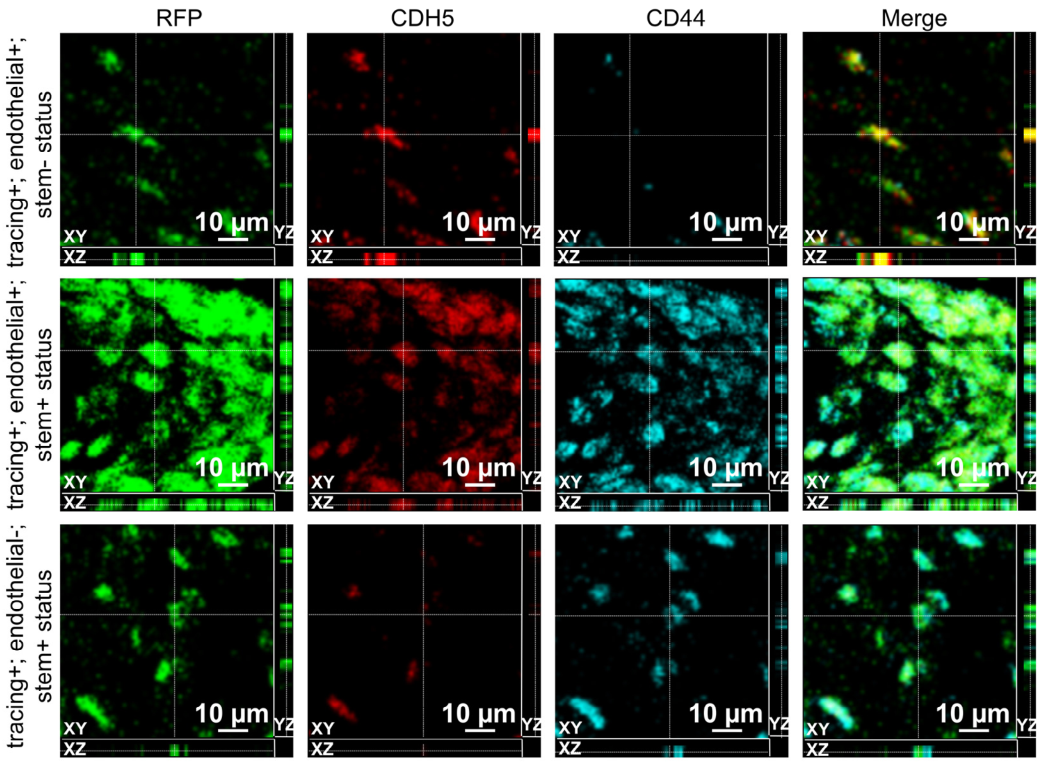

Supplemental Figure 2. The co-staining of RFP, CDH5, ad CD44 within the regenerated tissue of *CDH5-CreER^{T2}; Rosa26-LSL-Tdtomato* endothelial lineage tracing transgenic mice. RFP represents the lineage tracing status. CDH5 represents the endothelial status. CD44 represents the mesenchymal stem cell status. The tracing+; endothelial+; stem- status represents there is no EndMT. The tracing+; endothelial+; stem+ status represents there is partial EndMT. The tracing+; endothelial-; stem+ status represents there is full EndMT.

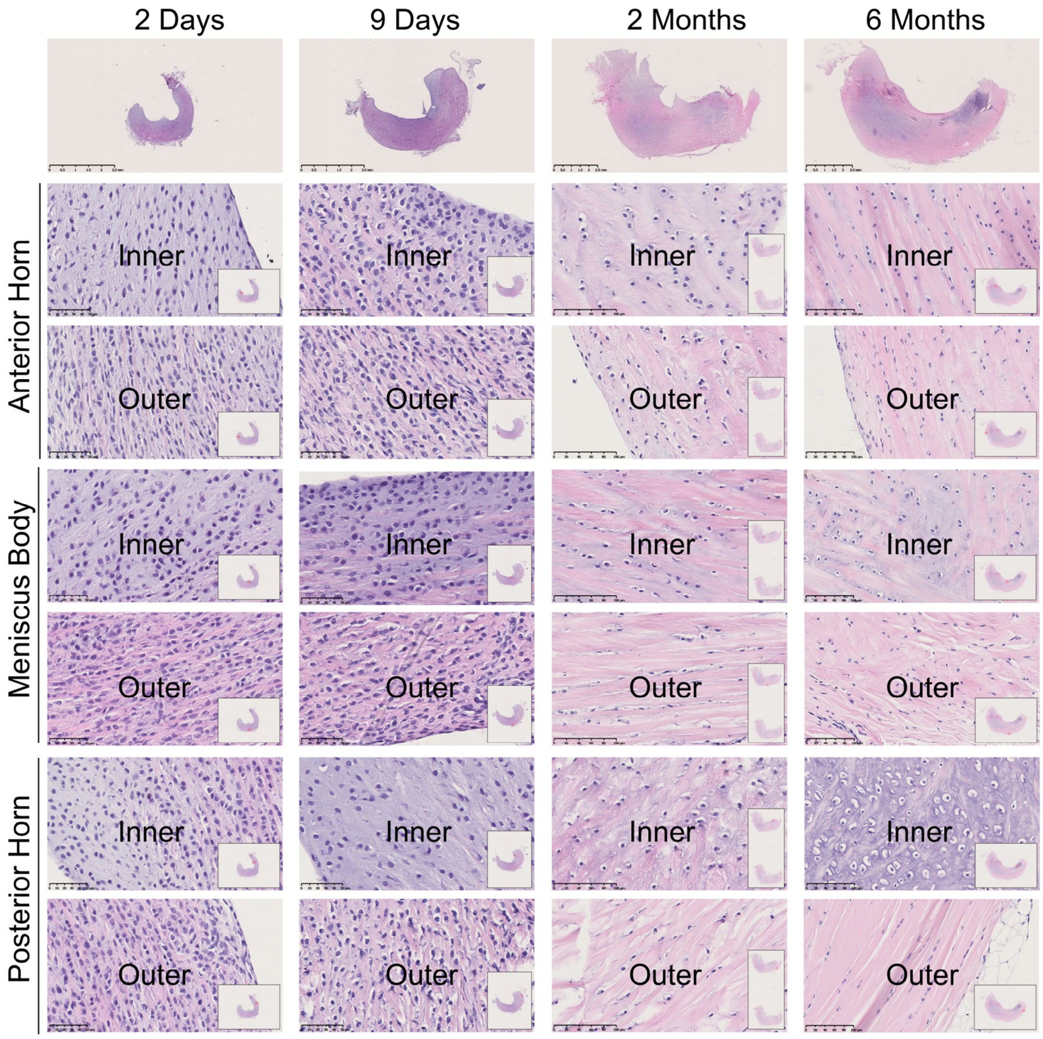

Supplemental Figure 3. The hematoxylin-eosin (HE) staining evaluation of meniscal cell morphology and fiber arrangement during maturation of native rabbit meniscus.

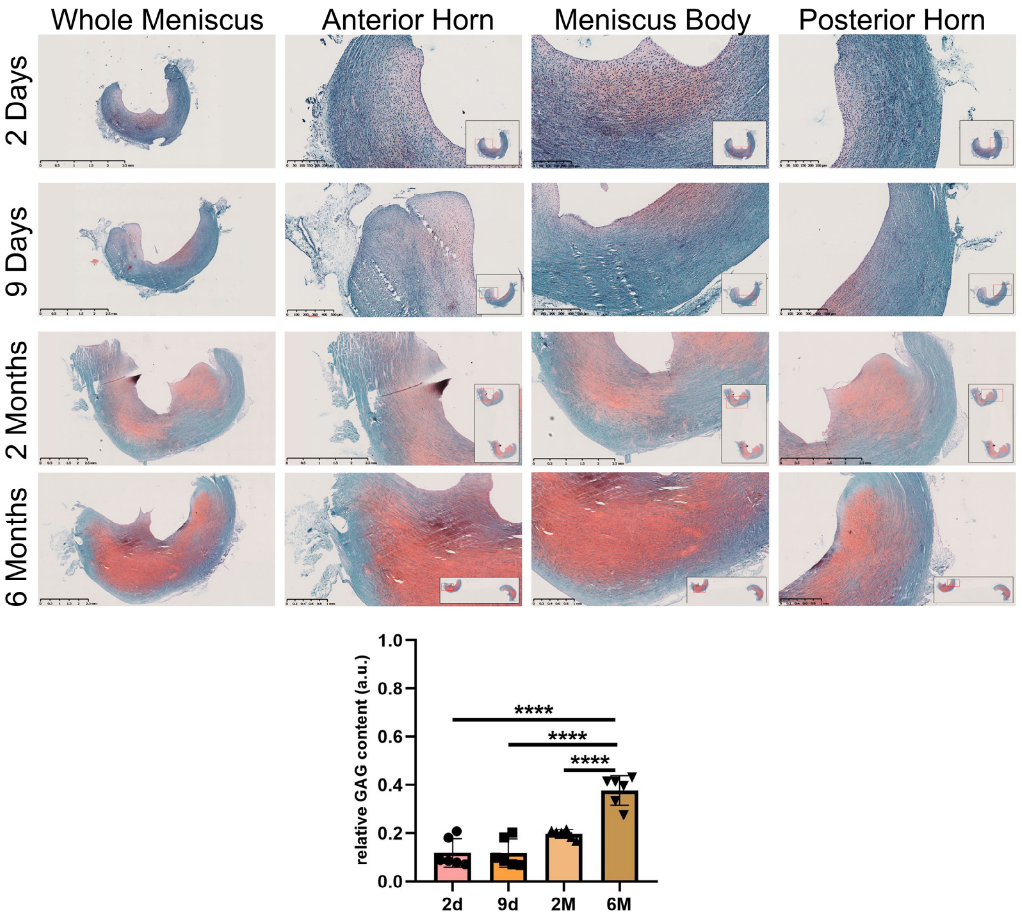

Supplemental Figure 4. The evaluation of GAG deposition by safranin O staining during maturation of native rabbit meniscus. A total of six ROIs were evaluated, one-way ANOVA, a.u. represents arbitrary unit, **** represents p<0.001.

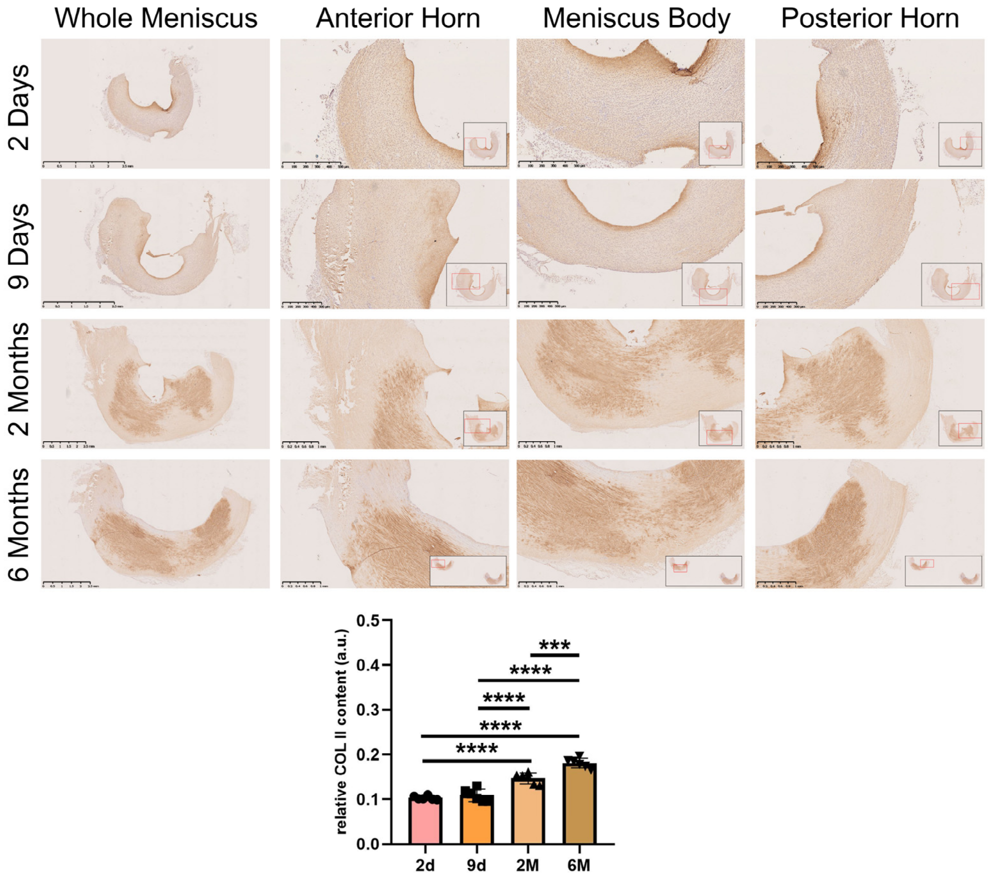

Supplemental Figure 5. The evaluation of COL II deposition by immunohistochemistry as the maturation of native rabbit meniscus. A total of six ROIs were evaluated, one-way ANOVA, a.u. represents arbitrary unit, *** represents p<0.001, **** represents p<0.001.

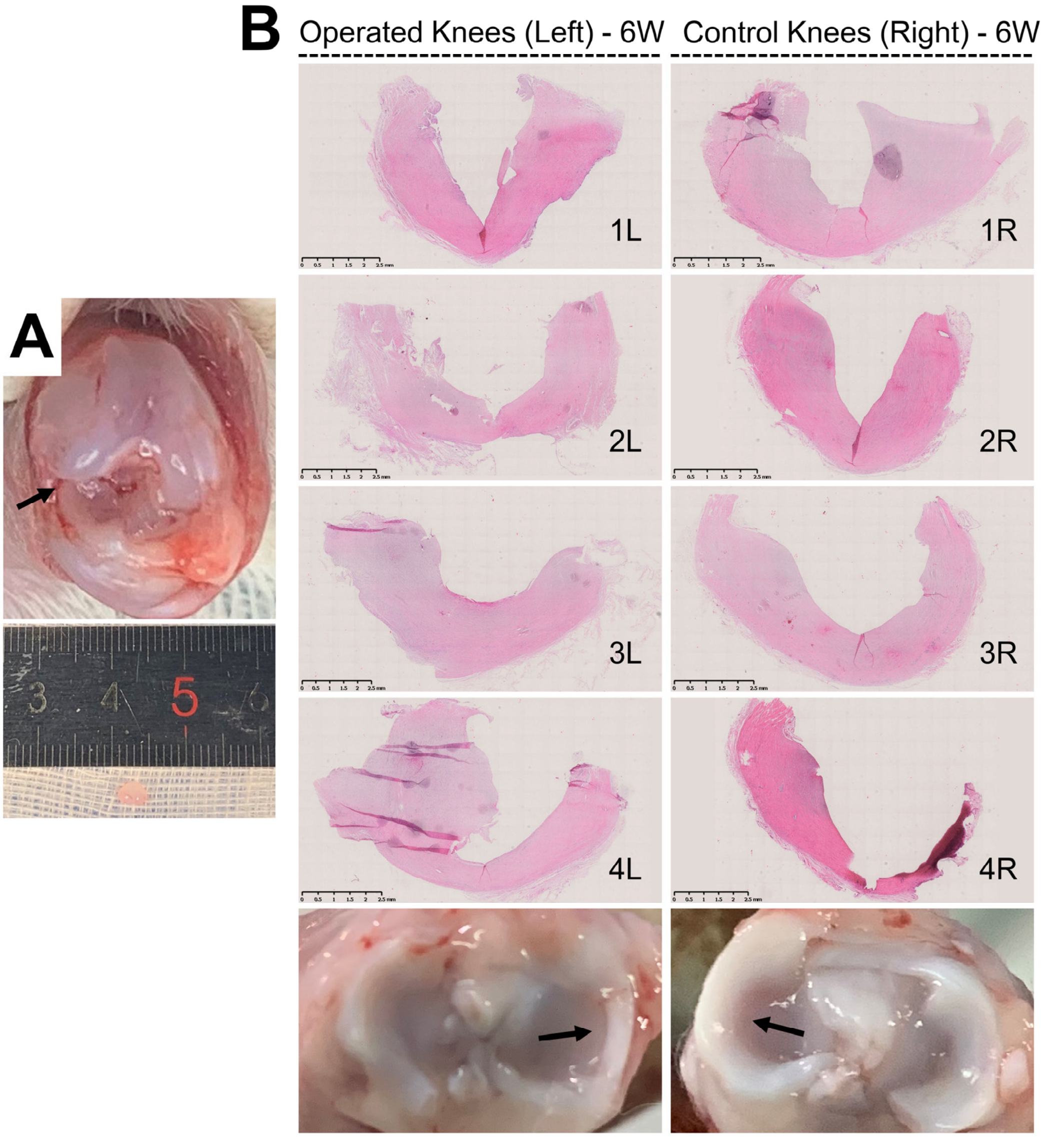

Supplemental Figure 6. The absence of mechanics affects meniscal morphology maturation of rabbit. (A) the preparation of condylectomy model in media femoral condyle of juvenile rabbit. (B) the histomorphology and macroscopic analysis of medial meniscus in the condylectomy knee and contralateral normal knee of juvenile rabbit, the black arrow represents medial meniscus, L represents left knee, R represents right knee.

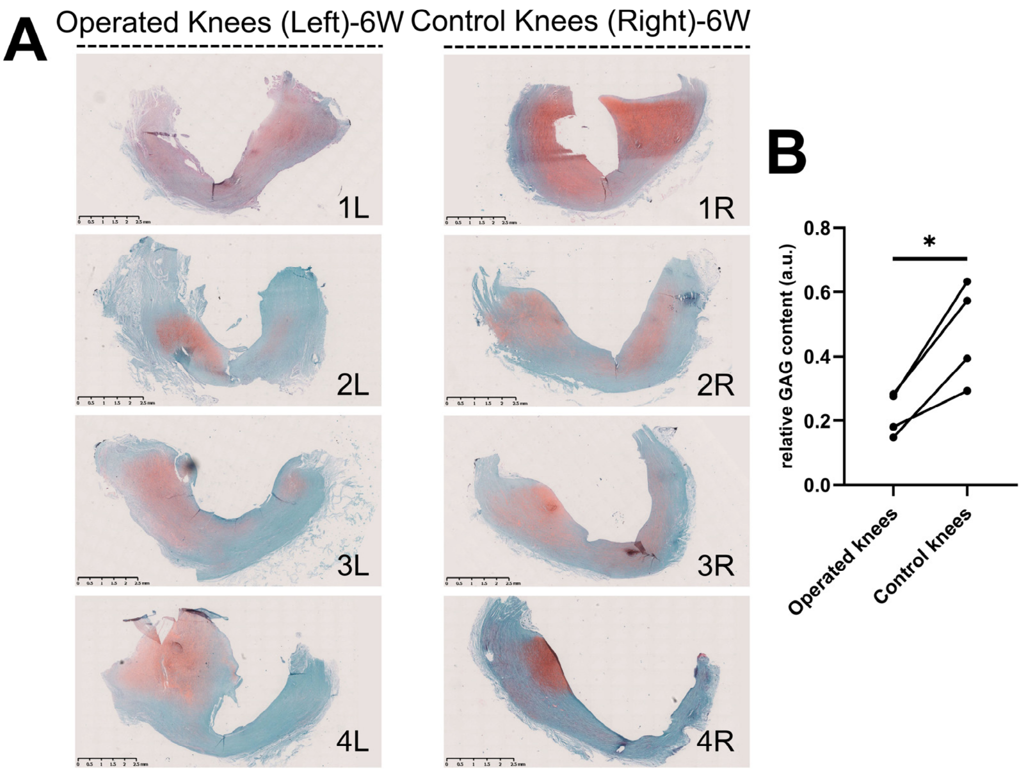

Supplemental Figure 7. The absence of mechanics affects GAG deposition during maturation of juvenile rabbit meniscus. (A) the safranin O staining evaluating GAG deposition in medial meniscus. (B) the before-after plot of GAG content. n=4, paired *t*-test, a.u. represents arbitrary unit, * represents $p<0.05$.

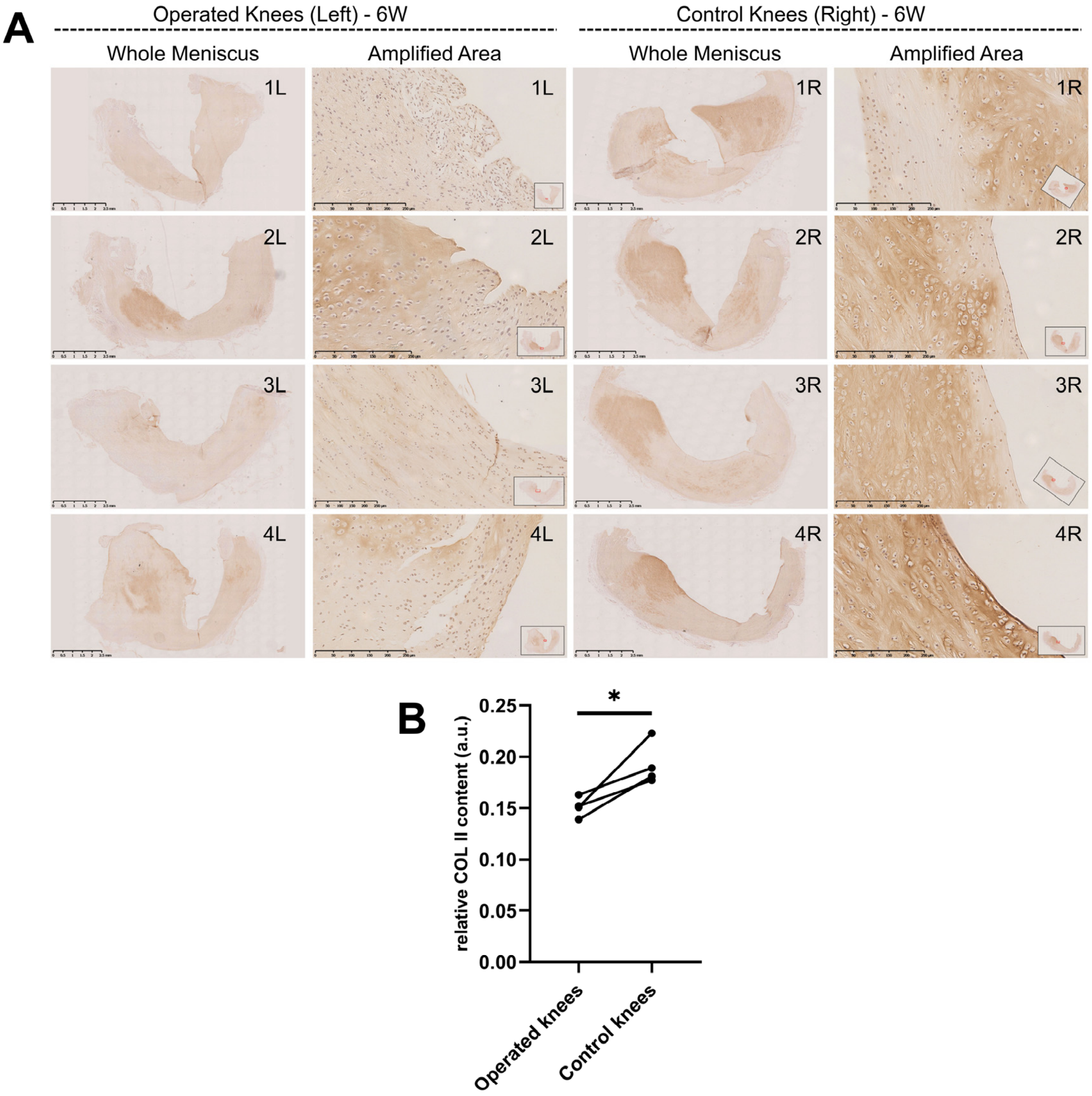

Supplemental Figure 8. The absence of mechanics affects COL II deposition during maturation of juvenile rabbit meniscus. (A) the COL II immunohistochemistry of medial meniscus. (B) the before-after plot of COL II content. n=4, paired *t*-test, a.u. represents arbitrary unit, * represents $p<0.05$.

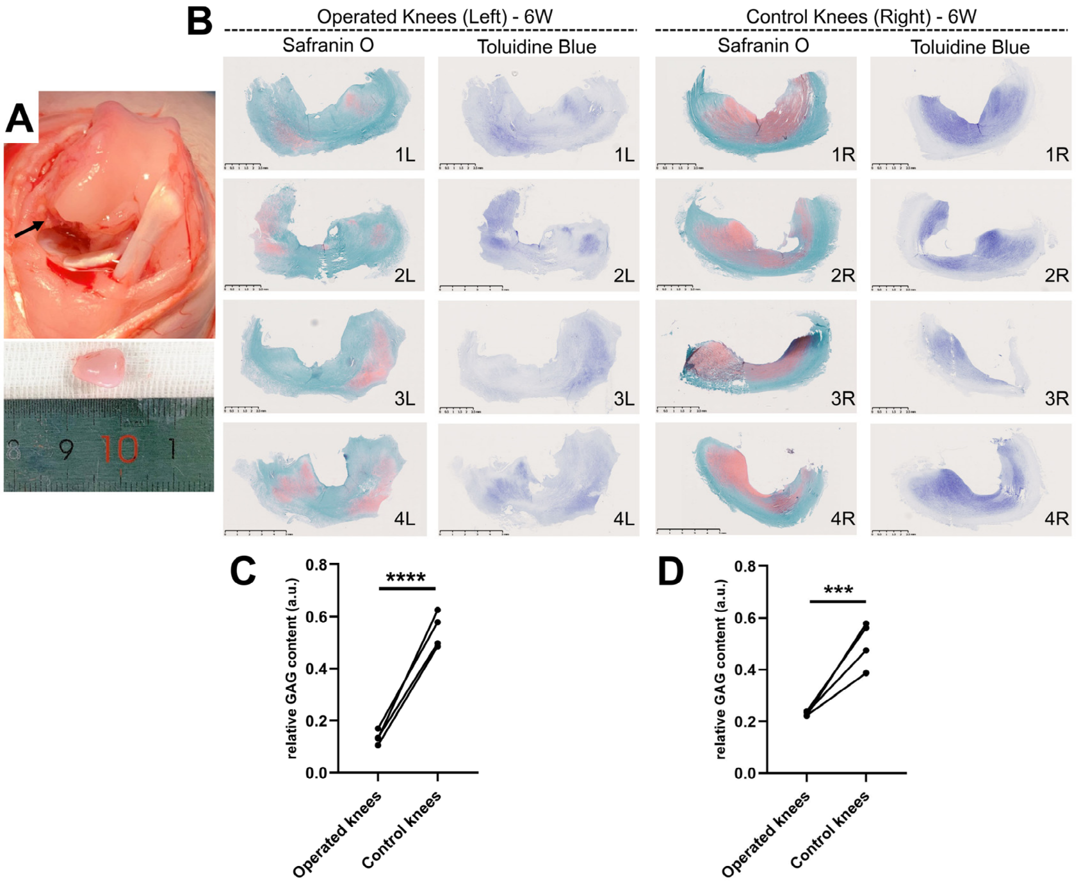

Supplemental Figure 9. The absence of mechanics causes GAG depletion in adult rabbit meniscus. (A) the preparation of condylectomy model in media femoral condyle of juvenile rabbit. (B) the safranin O and toluidine blue staining evaluating GAG deposition in medial meniscus of adult rabbit. (C) the before-after plot of GAG content reflected by safranin O staining. (D) the before-after plot of GAG content reflected by toluidine blue staining. n=4, paired *t*-test, a.u. represents arbitrary unit, *** represents p<0.001, **** represents p<0.001.

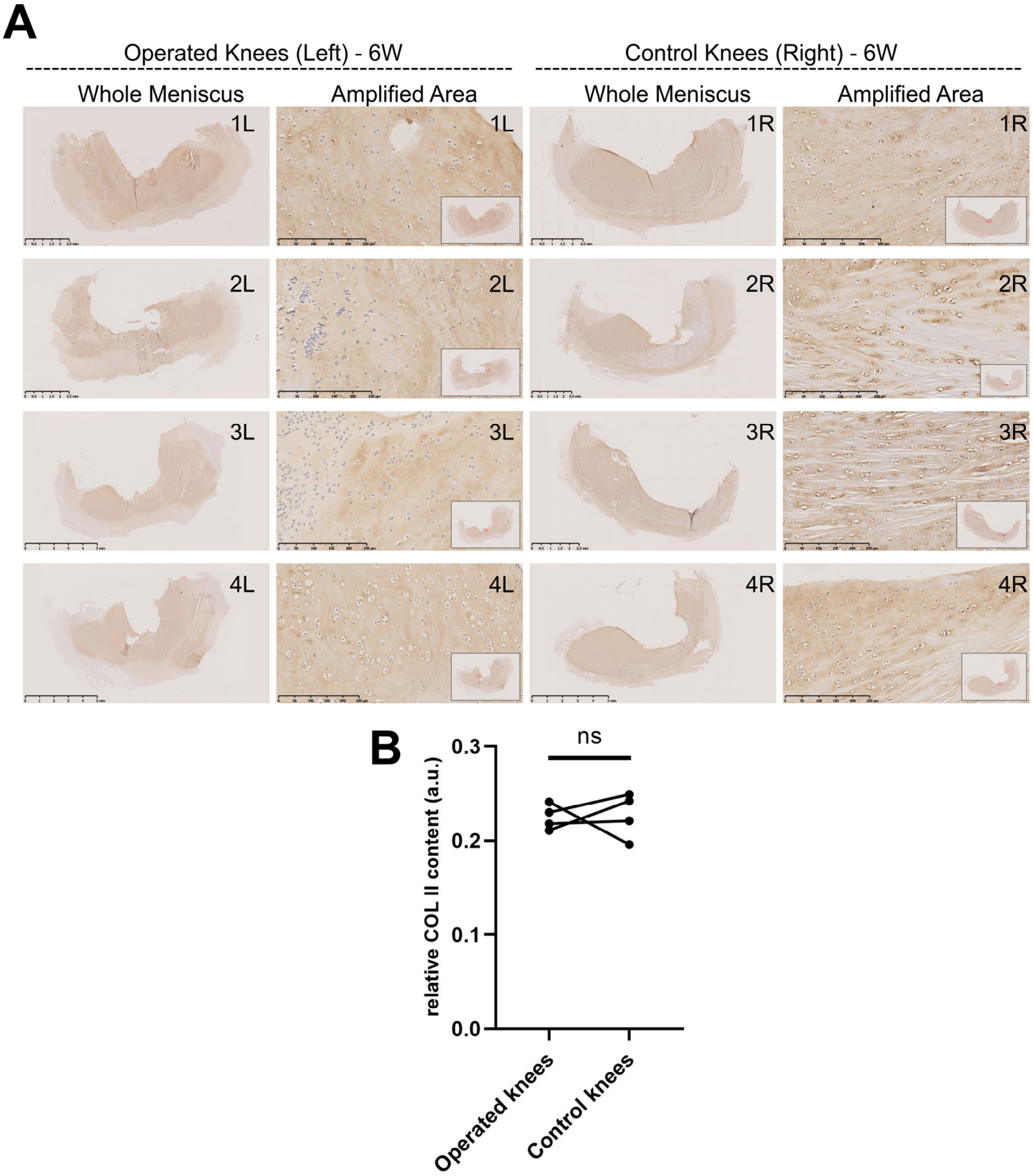

Supplemental Figure 10. The absence of mechanics affects COL II phenotype of adult rabbit meniscal cells. (A) the COL II immunohistochemistry of medial meniscus. (B) the before-after plot of COL II content. n=4, paired *t*-test, ns represents no significant difference.

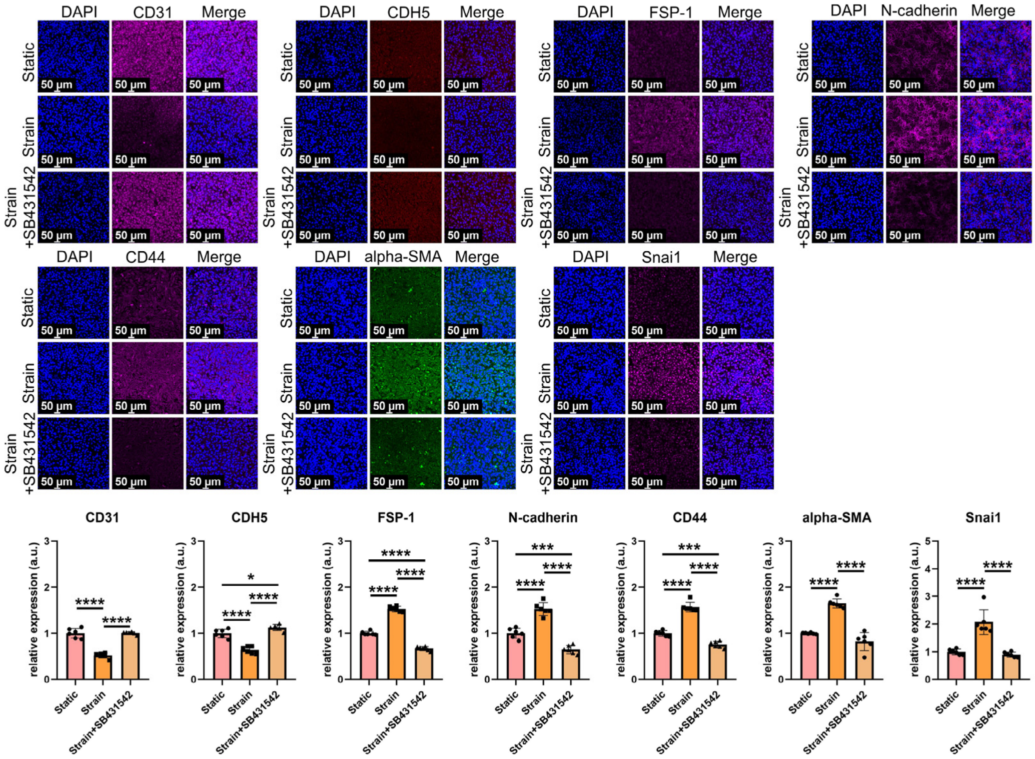

Supplemental Figure 11. EndMT inhibition by SB431542 reverted the molecular features induced by mechanical stimulus in HUVECs. SB-431542 is a TGFβ receptor kinase inhibitor. Six ROIs per biological sample were quantified, one-way ANOVA. a.u. represents arbitrary unit, ROIs represents region of interests, * represents *p*<0.05, *** represents *p*<0.001, **** represents *p*<0.001.

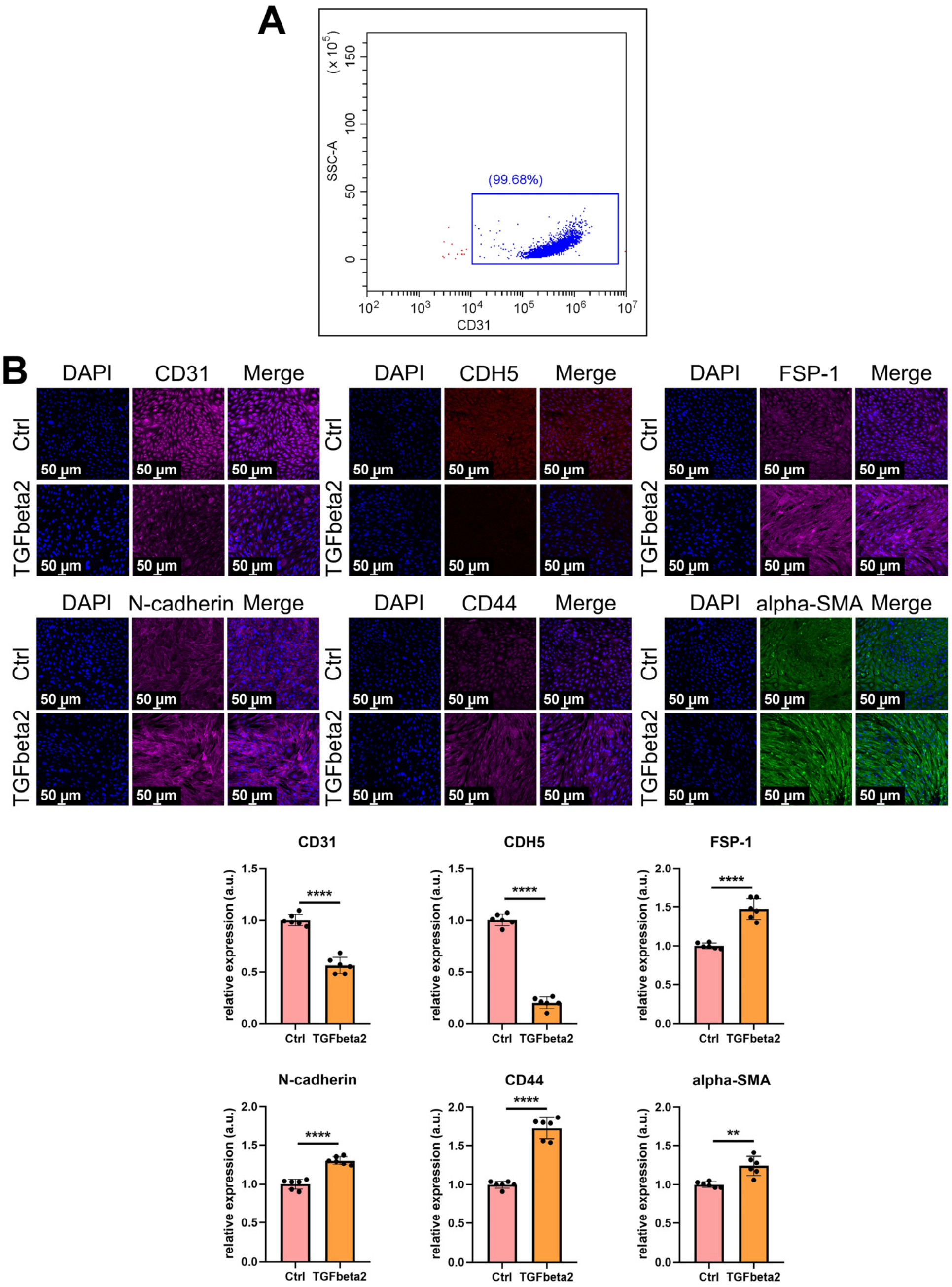

Supplemental Figure 12. The induction of EndMT status in rabbit endothelial cells. (A) the flow cytometry analysis of endothelial cells isolated from rabbit aorta. (B) the EndMT status was effectively achieved in rabbit endothelial cells after treated with TGFβ2 for 4 days. Six ROIs per biological sample were quantified, unpaired *t*-test. a.u. represents arbitrary unit, ROIs represents region of interests, ** represents $p<0.01$, **** represents $p<0.001$.

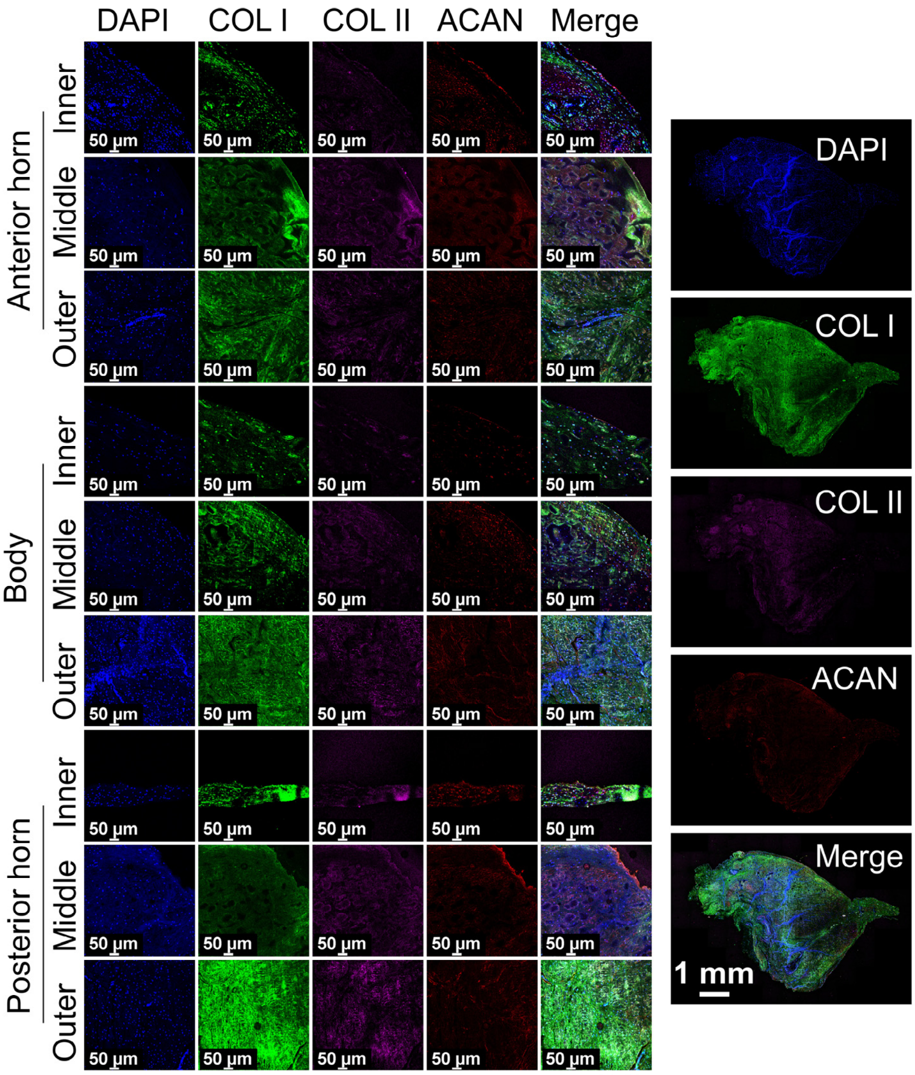

Supplemental Figure 13. The comprehensive immunofluorescent co-staining of COL I, COL II and ACAN within the regenerated tissue of Blank group of ovine. Left: the amplified images of specific zones, Right: the general images of whole specimen sections.

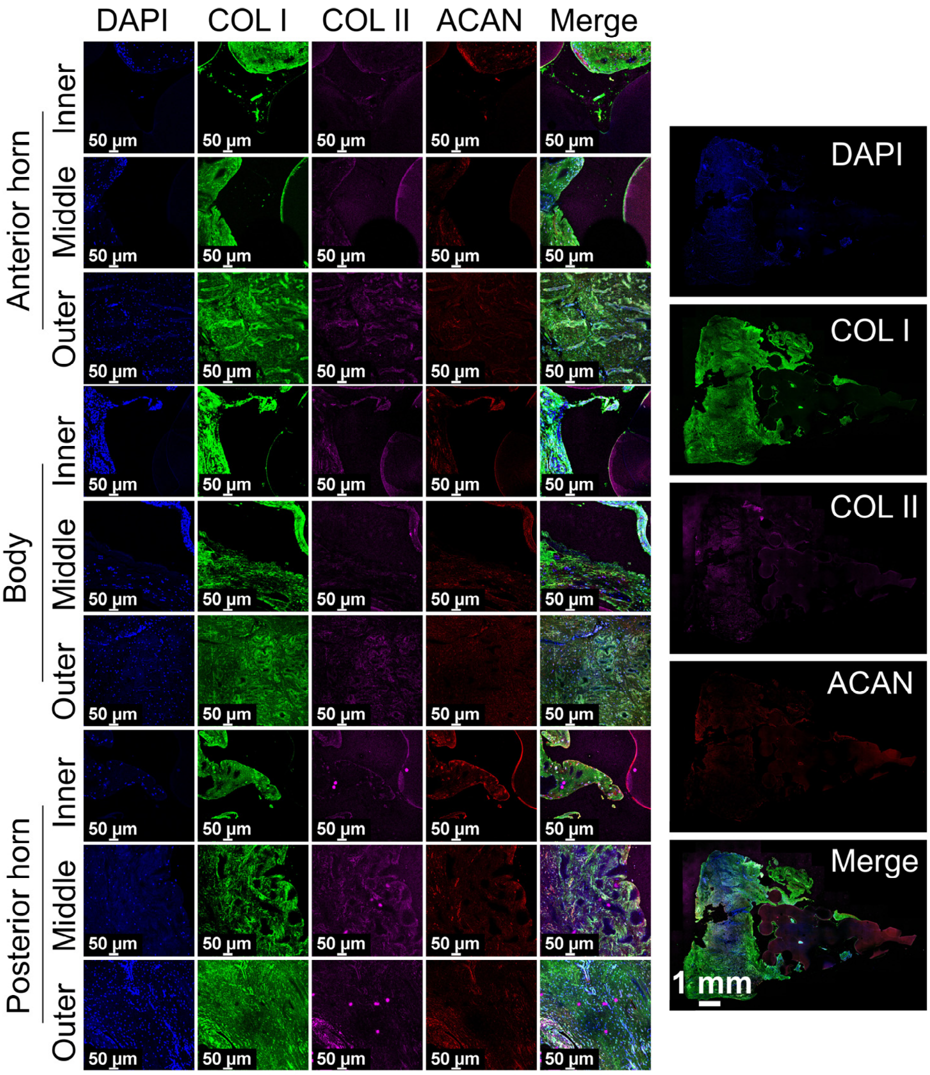

Supplemental Figure 14. The comprehensive immunofluorescent co-staining of COL I, COL II and ACAN within the regenerated tissue of Scaffold group of ovine. Left: the amplified images of specific zones, Right: the general images of whole specimen sections.

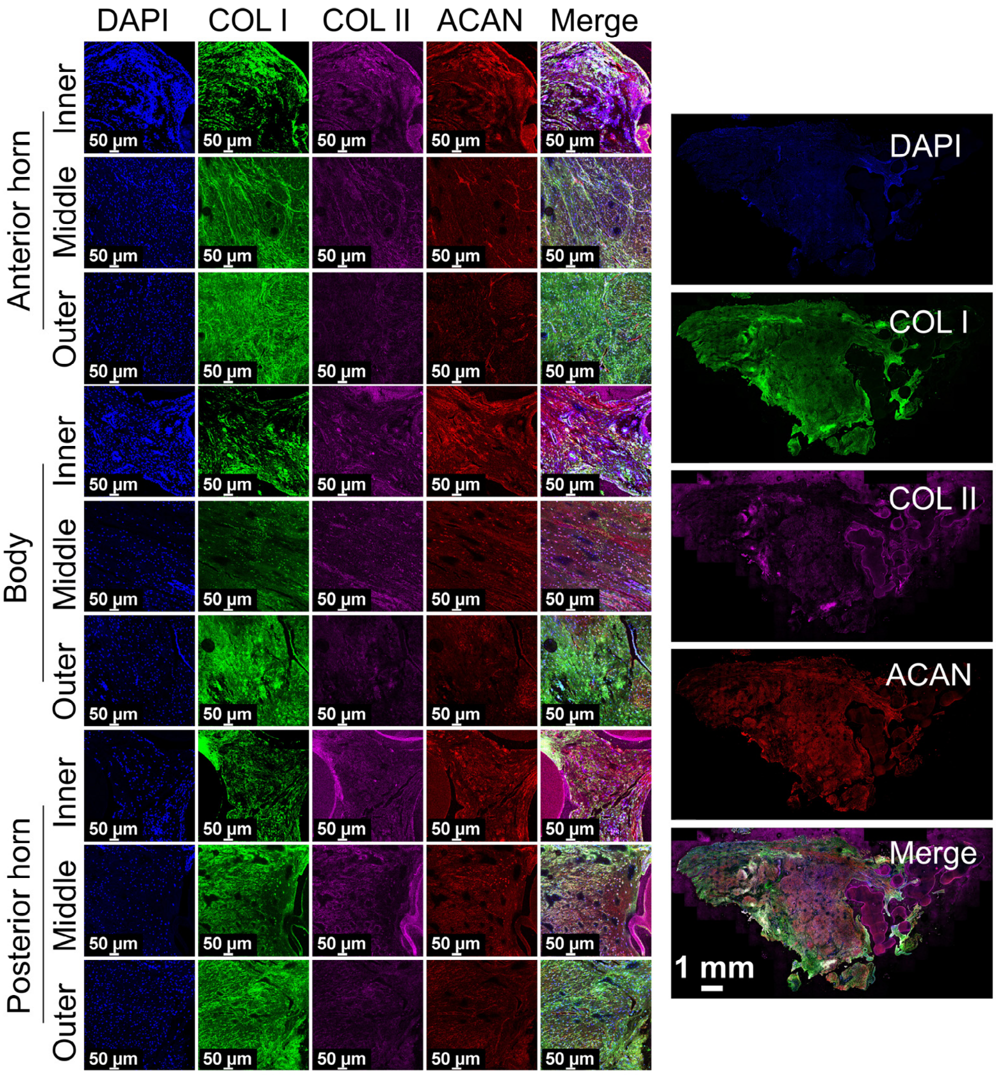

Supplemental Figure 15. The comprehensive immunofluorescent co-staining of COL I, COL II and ACAN within the regenerated tissue of Scaffold+exercise group of ovine. Left: the amplified images of specific zones, Right: the general images of whole specimen sections.

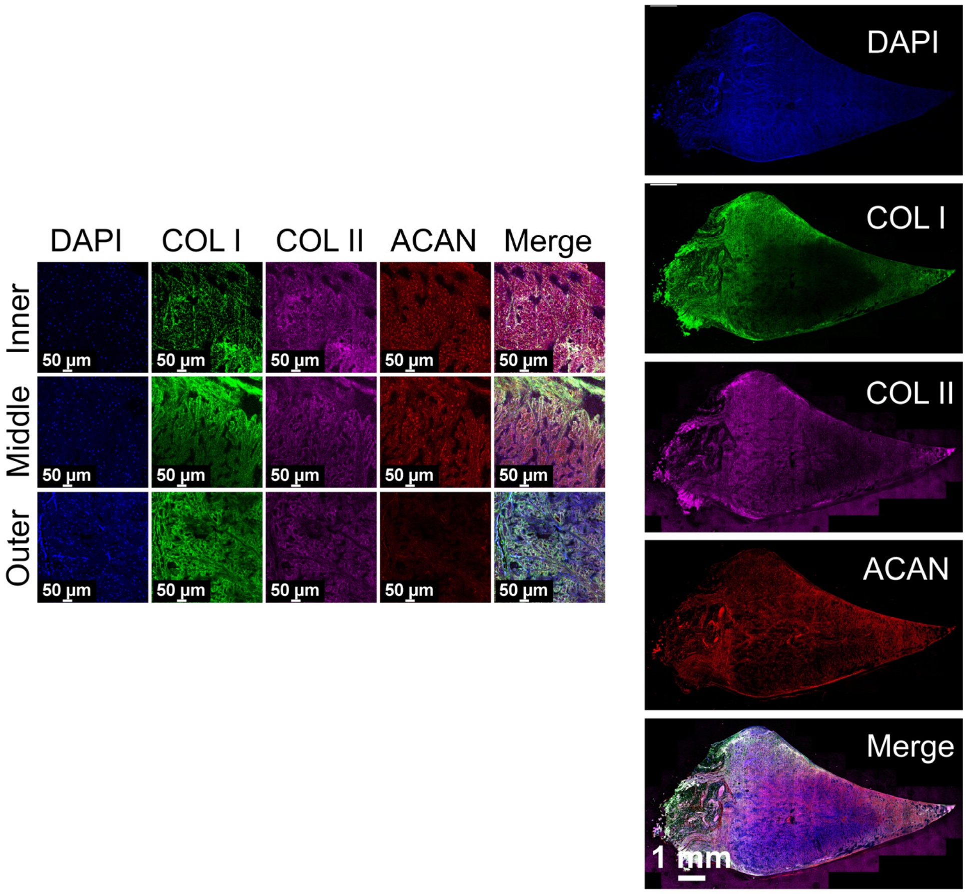

Supplemental Figure 16. The comprehensive immunofluorescent co-staining of COL I, COL II and ACAN within the native meniscus tissue of ovine. Left: the amplified images of specific zones, Right: the general images of whole specimen sections.

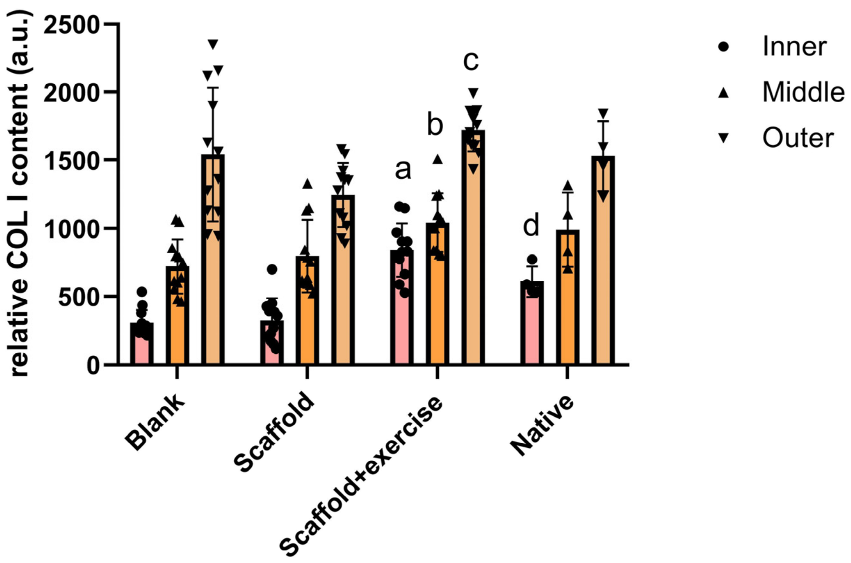

Supplemental Figure 17. The comprehensive semiquantitative analysis of COL I content of each group in ovine model. For Blank, Scaffold and Scaffold+exercise group, a total of twelve ROIs in the specified region of each group were quantified, for Native group, a total of four ROIs in the specified region were quantified, two-way ANOVA, a: p<0.05 compared to inner of Blank, Scaffold group; and outer of Scaffold+exercise group, b: p<0.05 compared to outer of Scaffold+exercise group, c: p<0.05 compared to outer of Scaffold group, d: p<0.05 compared to outer of native group. ROIs represents region of interests.

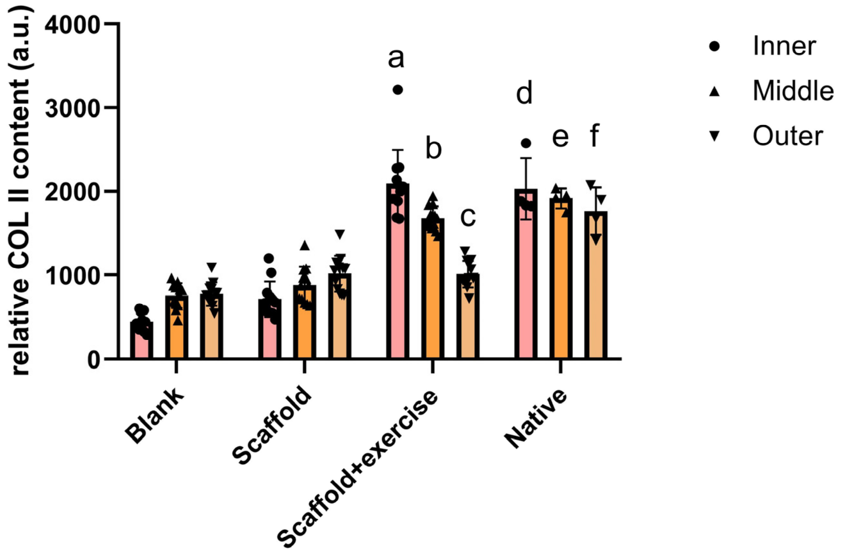

Supplemental Figure 18. The comprehensive semiquantitative analysis of COL II content of each group in ovine model. For Blank, Scaffold and Scaffold+exercise group, a total of twelve ROIs in the specified region of each group were quantified, for Native group, a total of four ROIs in the specified region were quantified, two-way ANOVA, a: p<0.05 compared to inner of Blank, Scaffold group; and middle, outer of Scaffold+exercise group, b: p<0.05 compared to middle of Blank, Scaffold group; and outer of Scaffold+exercise group, c: p<0.05 compared to outer of native group, d: p<0.05 compared to inner of Blank, Scaffold group, e: p<0.05 compared to middle of Blank, Scaffold group, f: p<0.05 compared to outer of Blank, Scaffold group. ROIs represents region of interests.

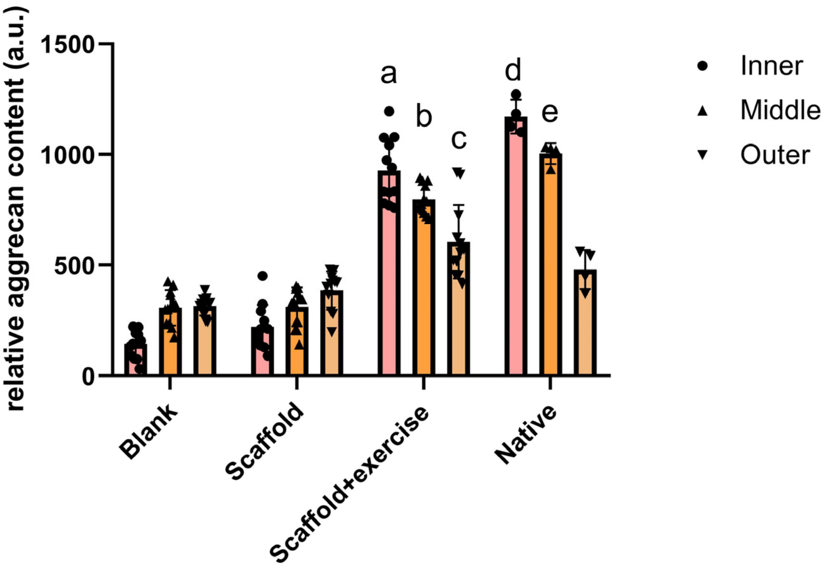

Supplemental Figure 19. The comprehensive semiquantitative analysis of aggrecan content of each group in ovine model. For Blank, Scaffold and Scaffold+exercise group, a total of twelve ROIs in the specified region of each group were quantified, for Native group, a total of four ROIs in the specified region were quantified, two-way ANOVA, a: $p<0.05$ compared to inner of Blank, Scaffold, native group; and outer of Scaffold+exercise group, b: $p<0.05$ compared to middle of Blank, Scaffold, native group; and outer of Scaffold+exercise group, c: $p<0.05$ compared to outer of Blank, Scaffold group, d: $p<0.05$ compared to inner of Blank, Scaffold group; and outer of native group, e: $p<0.05$ compared to middle of Blank, Scaffold group; and outer of native group. ROIs represents region of interests.

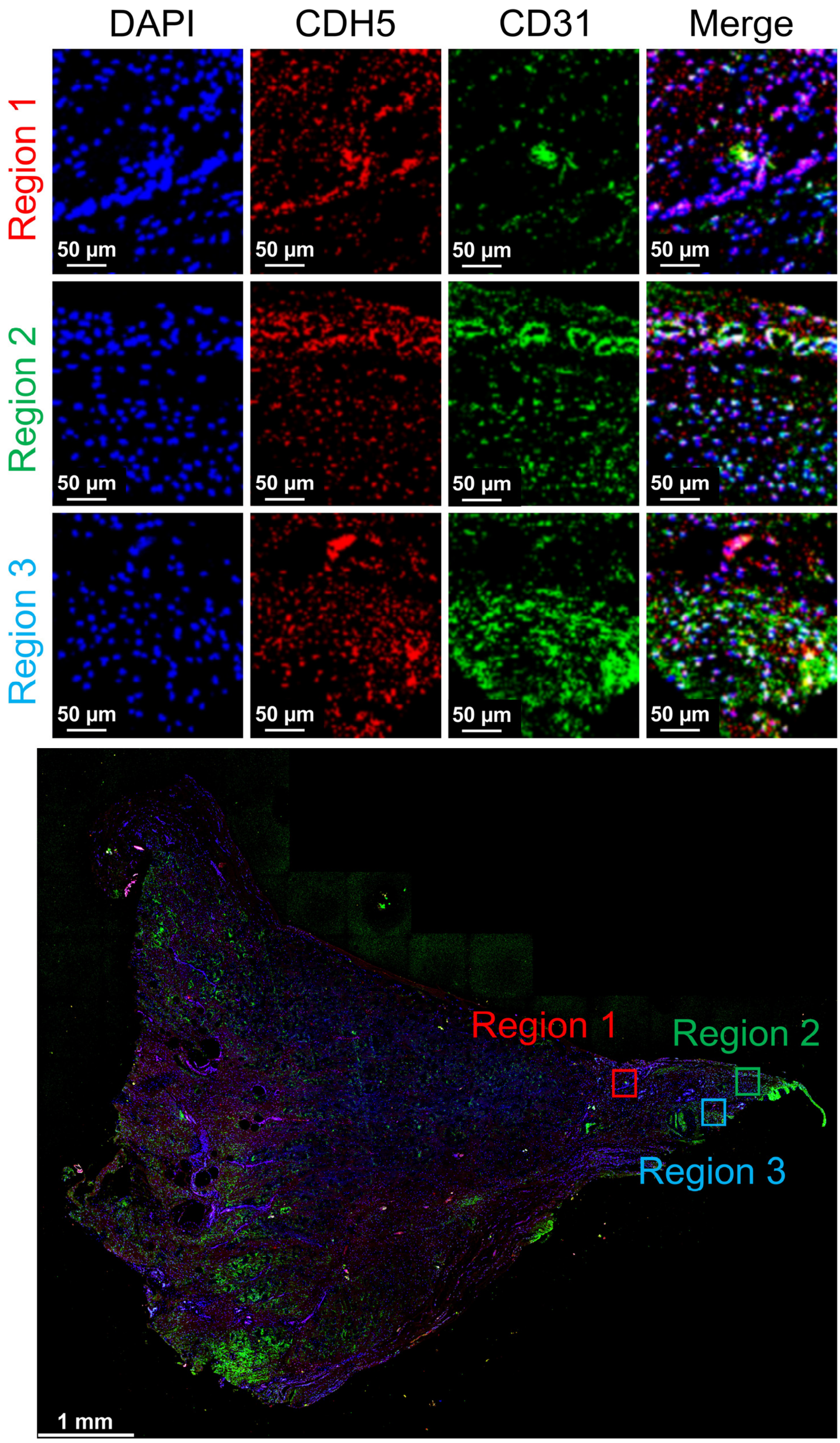

Supplemental Figure 20. The comprehensive analysis of vascularization using co-staining of CDH5 and CD31 within the regenerated tissue of Blank group in ovine.

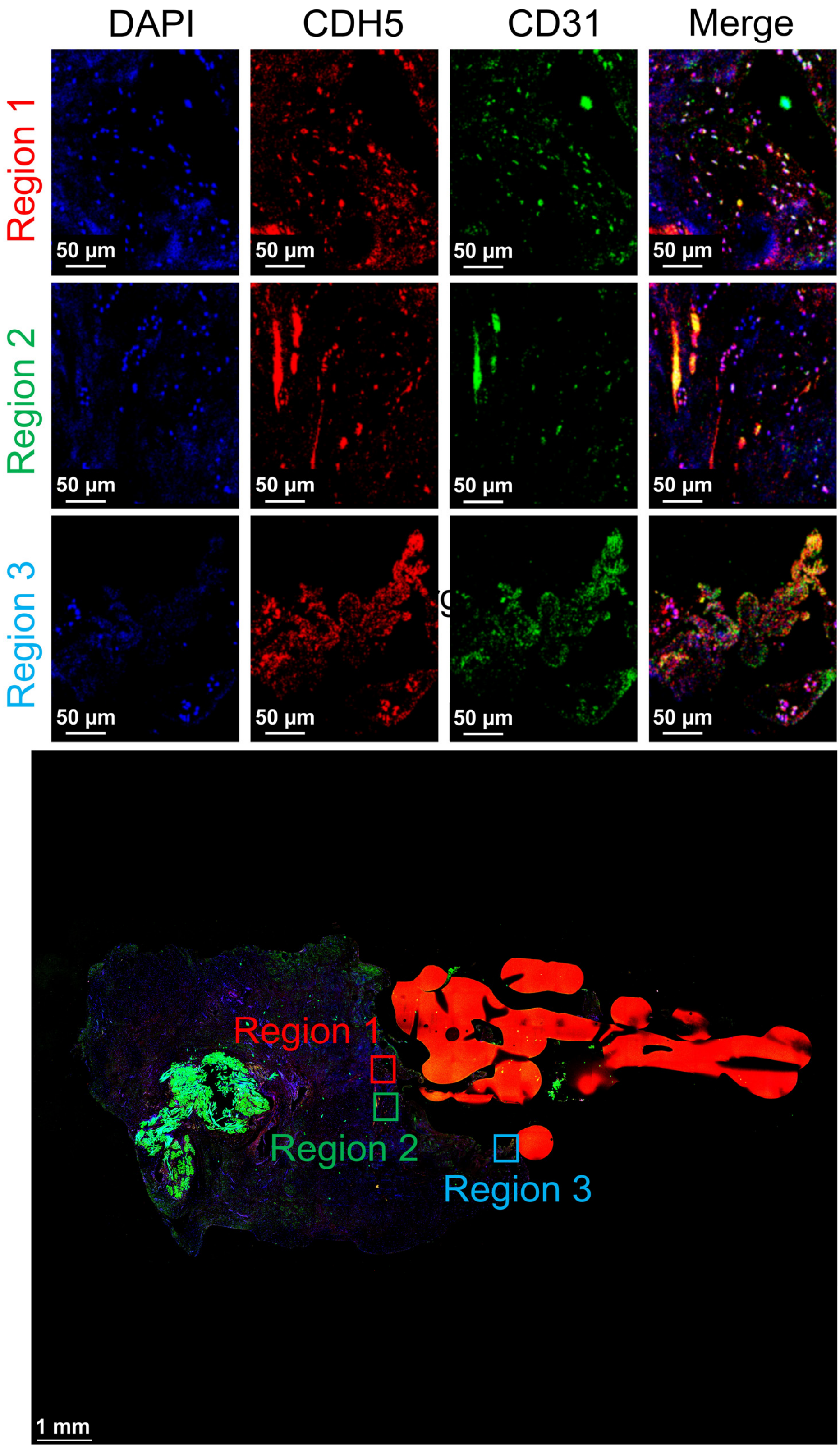

Supplemental Figure 21. The comprehensive analysis of vascularization using co-staining of CDH5 and CD31 within the regenerated tissue of Scaffold group in ovine.

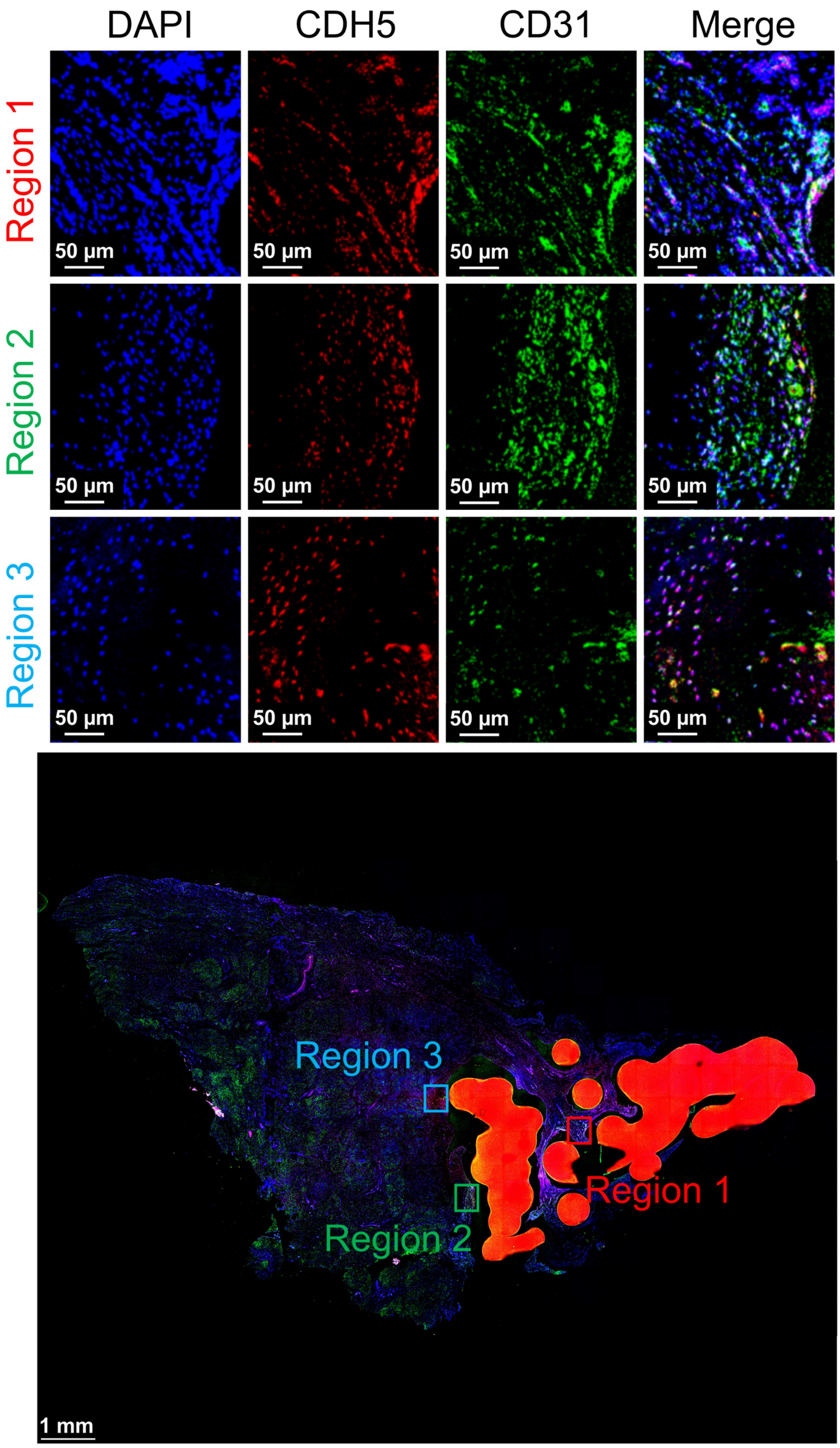

Supplemental Figure 22. The comprehensive analysis of vascularization using co-staining of CDH5 and CD31 within the regenerated tissue of Scaffold+exercise group in ovine.

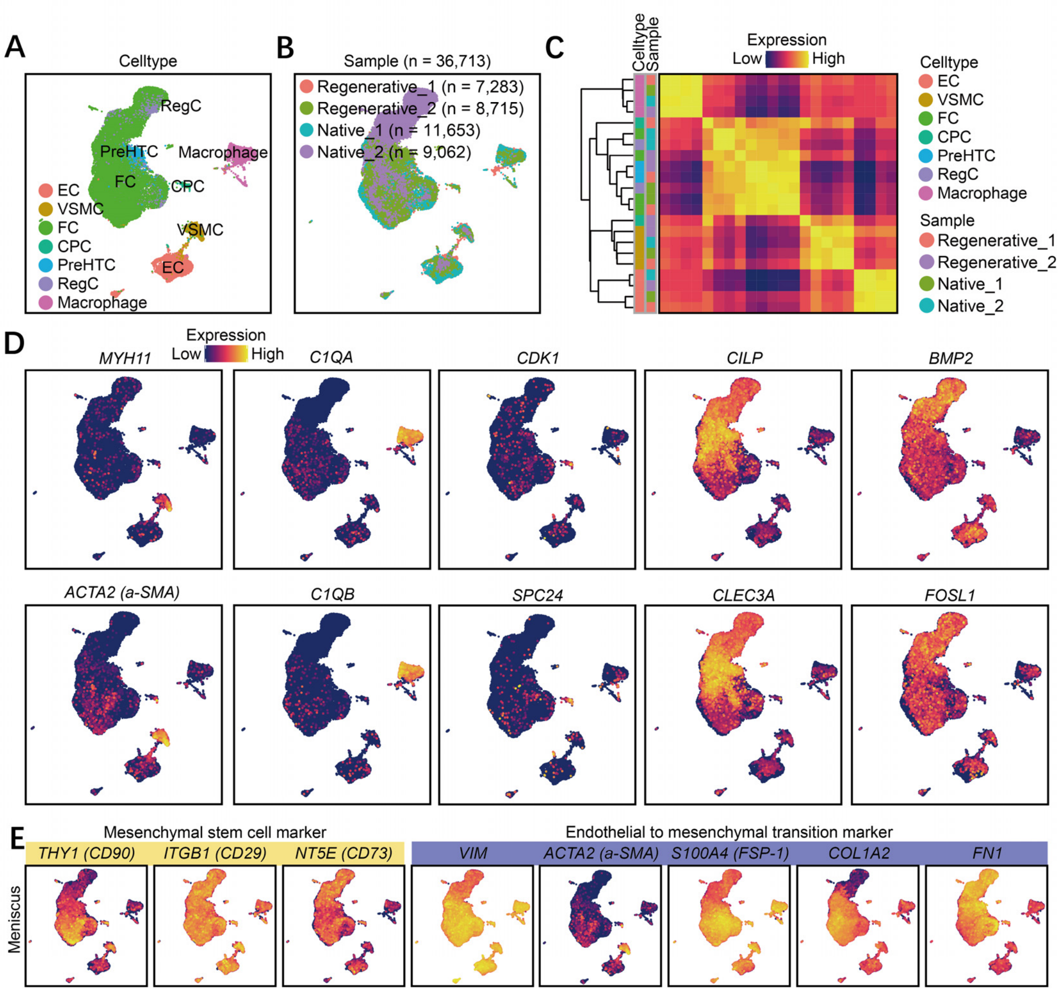

Supplemental Figure 23. The scRNA-seq analysis reveals mesenchymal signatures in the regenerated menisci. (A-B) all meniscal cell from four samples colored by cell types (A) and samples (B) in UMAP, the single-cell data contain ovine samples sequenced in this research, Regenerative_1 (n = 7,283) and Native_1 (n = 11,653), and beagle canine samples in our previous study, Regenerative_2 (n = 8,715) and Native_2 (n = 9,062). (C) the heatmap of correlation between all meniscal cell grouped by both samples and celltypes, the annotation on the left of the heatmap showing the celltype (left) and the sample (right) information. (D) the expression of marker genes of VSMC (*MYH11* and *ACTA2*), macrophage (*C1QA* and *C1QB*), CPC (*CDK1* and *SPC24*), PreHTC (*CILP* and *CLEC3A*), and RegC (*BMP2* and *FOSL1*) presented in UMAP. (E) the marker gene

expression of mesenchymal stem cell (*CD90*, *CD29*, and *CD73*) and endothelial to mesenchymal transition (*VIM*, *ACTA2*, *S100A4*, *COL1A2*, and *FN1*) in UMAP of meniscal cells.

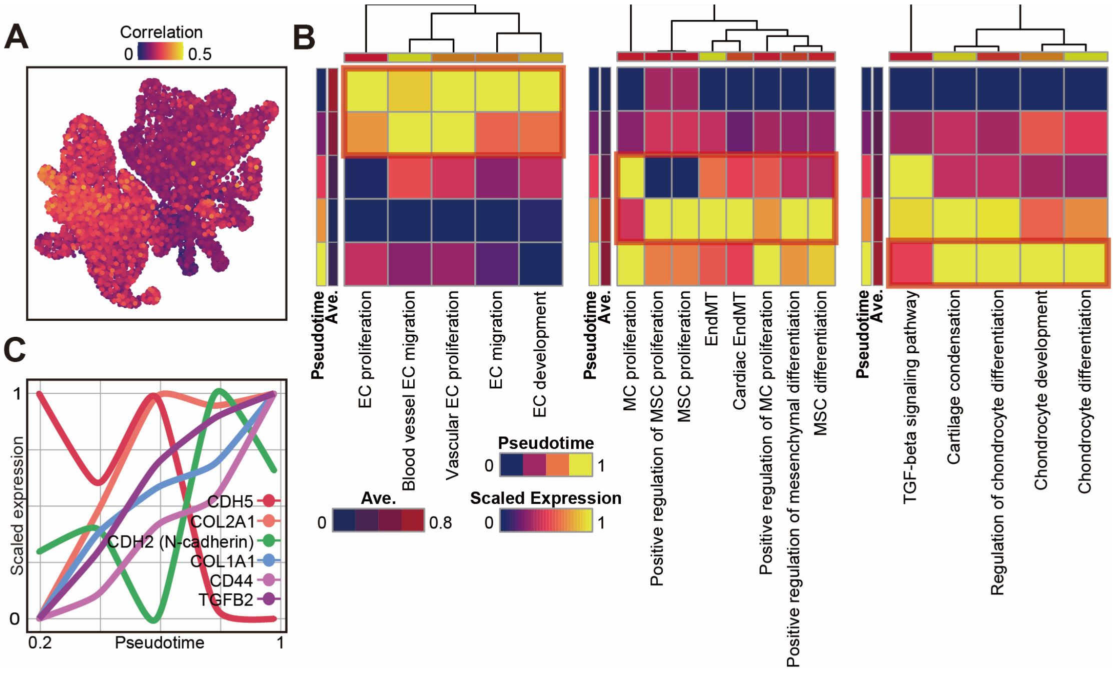

Supplemental Figure 24. The GO analysis and key gene expression profiles during endothelial to mesenchymal transition in the menisci. (A) the maximum of correlation scores between mesenchymal stem cells and meniscal cells presented in the force-directed graph of meniscal EC and FC. (B) the enrichment of GO terms, including EndMT, cardiac EndMT, MSC proliferation, mesenchymal differentiation, and mesenchymal cell proliferation), and Kyoto encyclopedia of genes and genomes (KEGG) term, TGF-beta signaling pathway, along the pseudotime. (C) the expression of marker genes along the pseudotime.

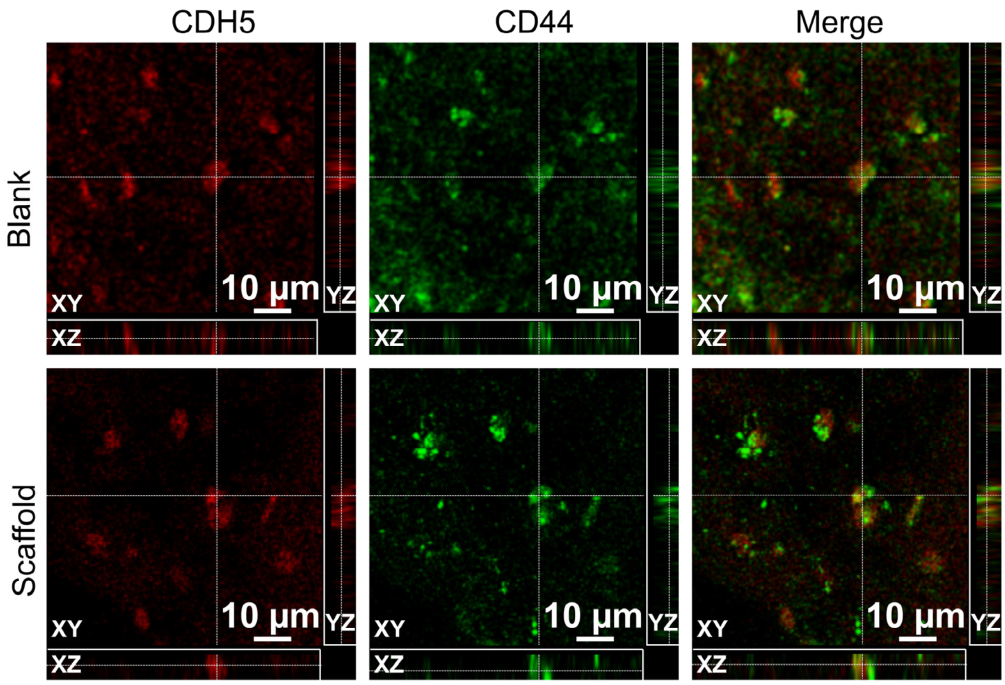

Supplemental Figure 25. The orthogonal projection of CDH5 and CD44 within the regenerated tissue of ovine Blank and Scaffold group.

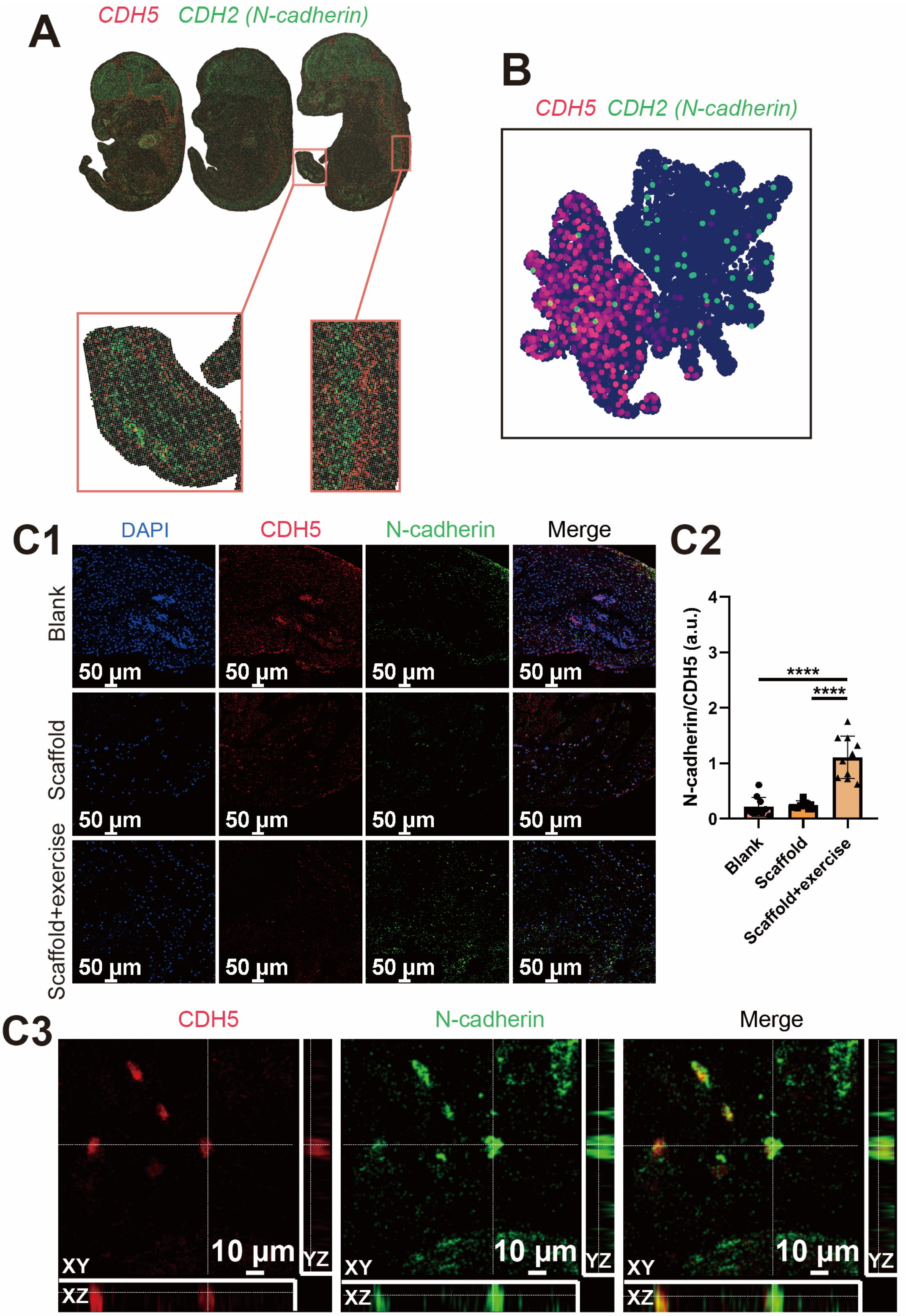

Supplemental Figure 26. The colocalization of CDH5 and N-cadherin within mouse organogenesis and ovine regenerated tissue. (A) the spatiotemporal transcriptomic atlas during mouse organogenesis showing the expression of *CDH5* (red) and *N-cadherin*

(green). (B) the co-expression of *CDH5* (red) and *N-cadherin* (green) presented in the single-cell UMAP of meniscal EC and FC. (C) the immunofluorescent colocalization of CDH5 and N-cadherin within newly regenerated tissue of ovine (C1, the immunofluorescent staining; C2, the semiquantitative analysis of N-cadherin fluorescence intensity divided by CDH5 fluorescence intensity in each group, a total of ten slices per group were evaluated, one-way ANOVA; C3, the orthogonal projection of CDH5 and N-cadherin within the regenerated tissue of Scaffold+exercise group, which confirmed the colocalization of CDH5 and N-cadherin in 3D view). a.u. represents arbitrary unit, **** represents p<0.001.

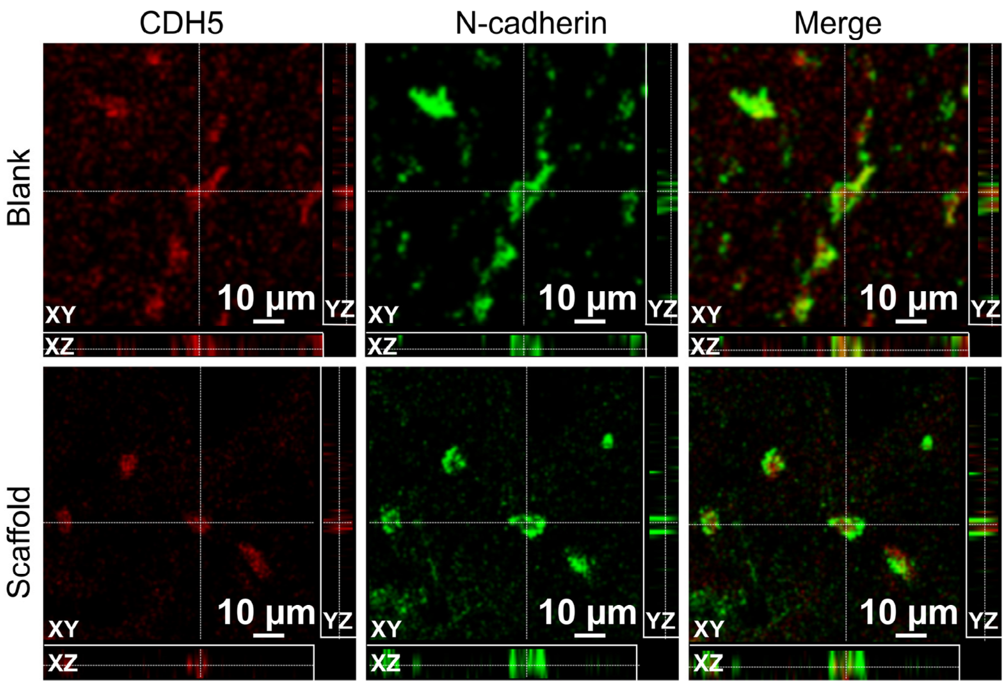

Supplemental Figure 27. The orthogonal projection of CDH5 and N-cadherin within the regenerated tissue of ovine Blank and Scaffold group.

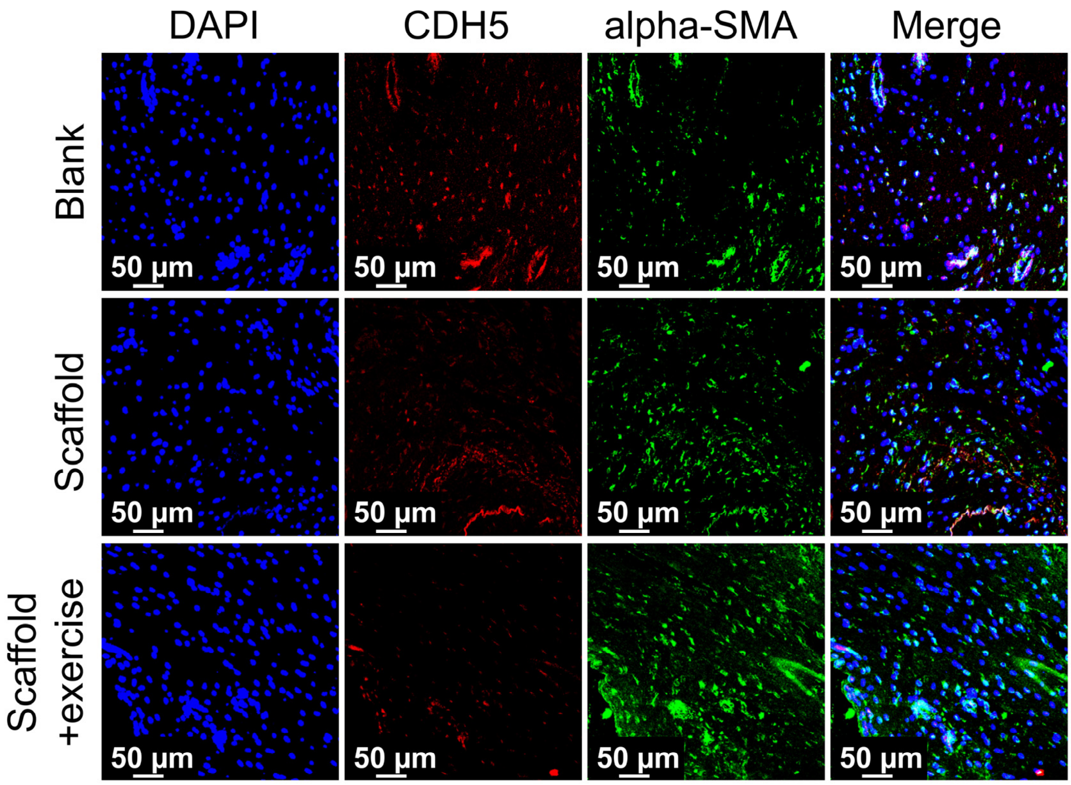

Supplemental Figure 28. The immunofluorescent colocalization of CDH5 and alpha-SMA within the regenerated tissue of ovine Blank, Scaffold and Scaffold+exercise group.

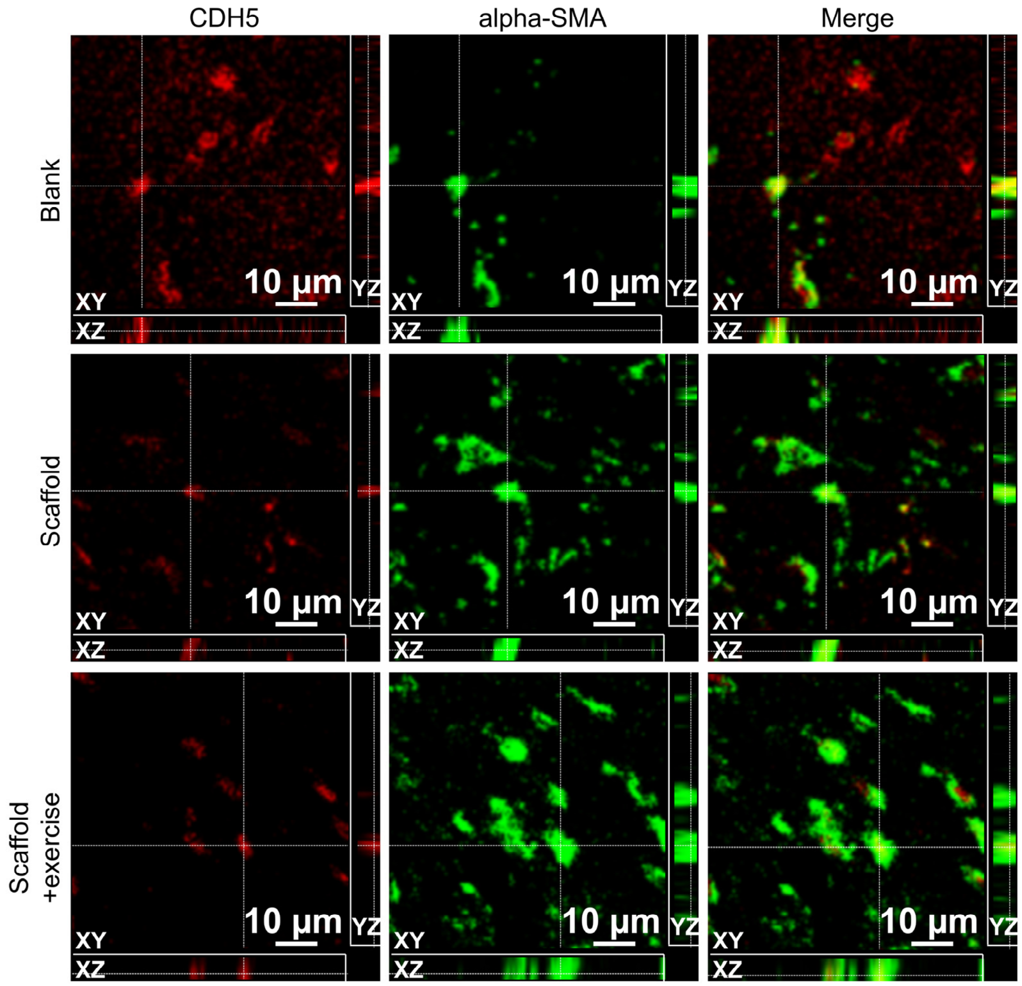

Supplemental Figure 29. The orthogonal projection of CDH5, alpha-SMA within the regenerated tissue of ovine Blank, Scaffold and Scaffold+exercise group.

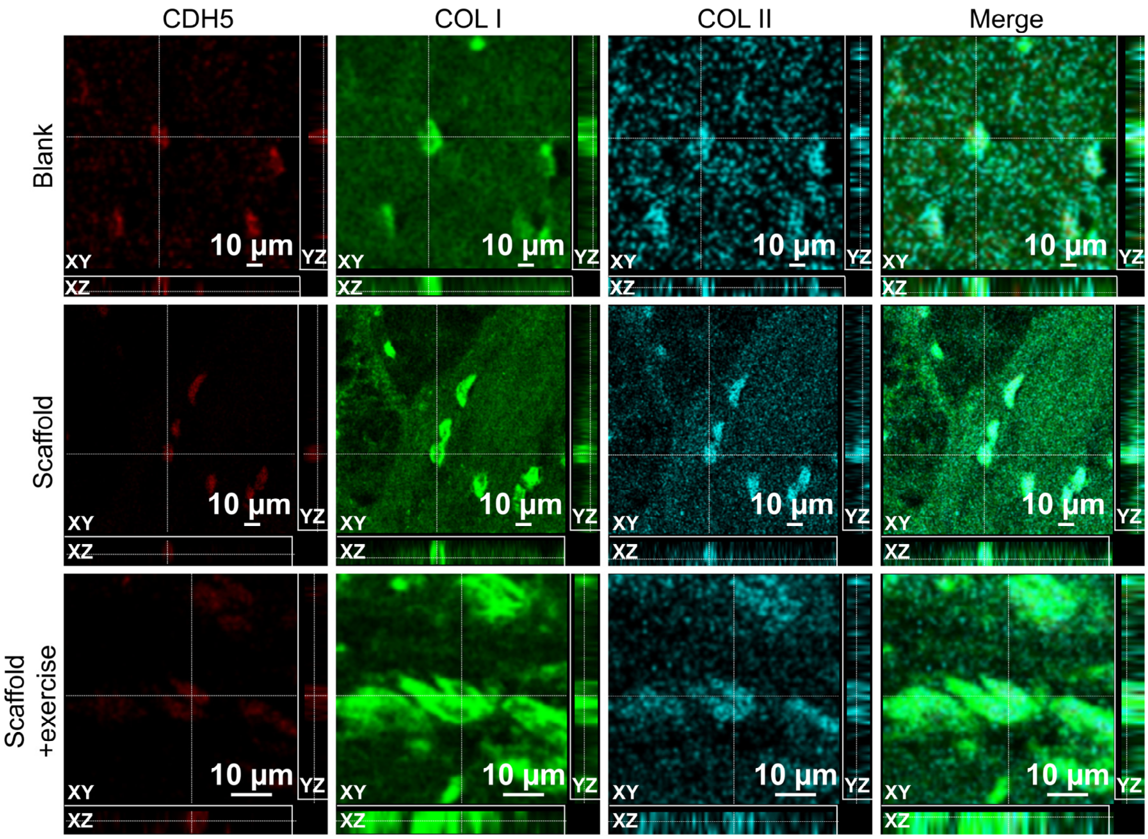

Supplemental Figure 30. The orthogonal projection of CDH5, COL I and COL II within the regenerated tissue of ovine Blank, Scaffold and Scaffold+exercise group.

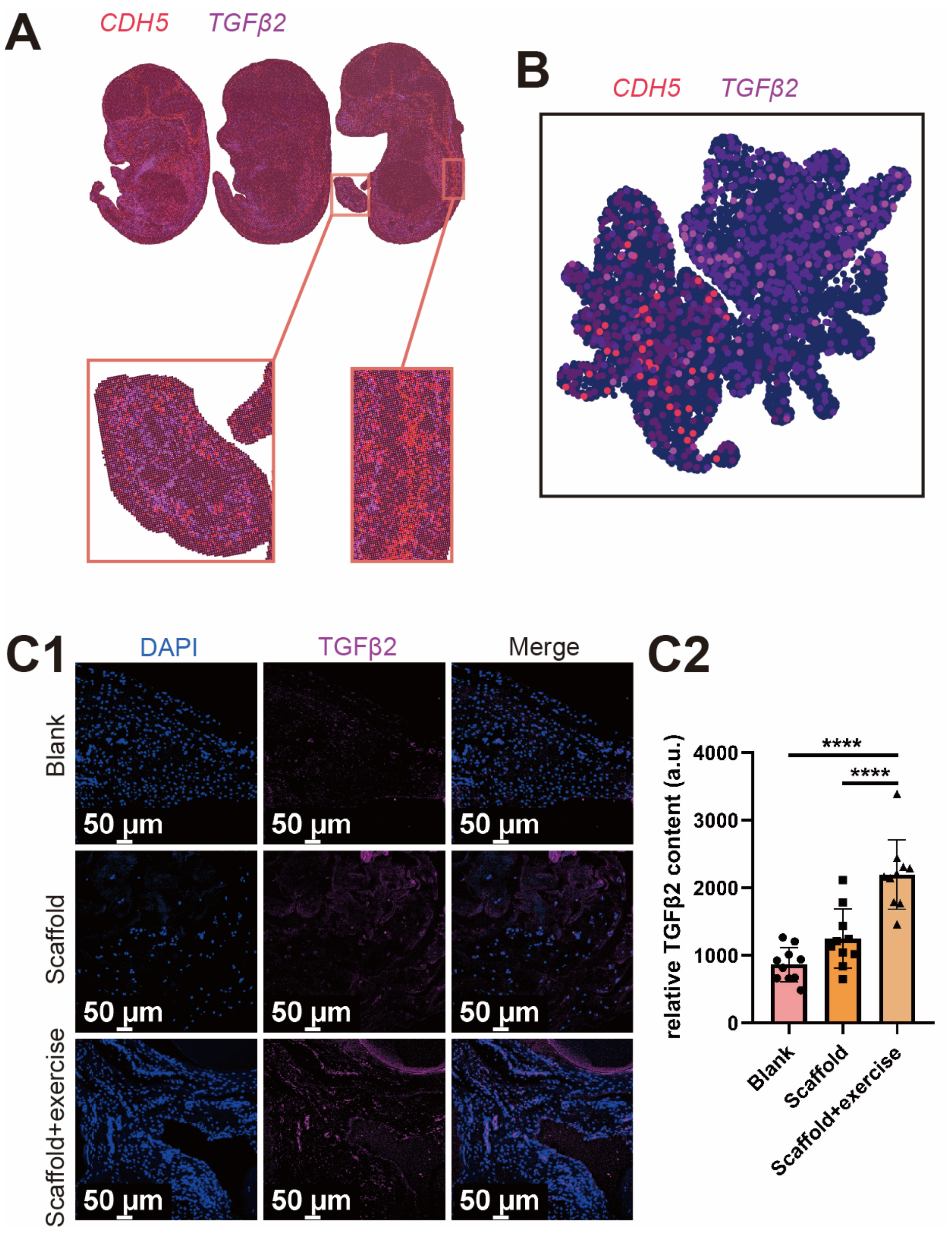

Supplemental Figure 31. The expression of TGFbeta2 within mouse organogenesis and ovine regenerated tissue. (A) the spatiotemporal transcriptomic atlas during mouse organogenesis showing the expression of *CDH5* (red) and *TGFbeta2* (purple). (B) the co-expression of *CDH5* (red) and *TGFbeta2* (purple) presented in the single-cell UMAP of meniscal EC and FC. (C) the immunofluorescence of TGFbeta2 within newly

regenerated tissue of ovine (C1, the immunofluorescent staining; C2, the semiquantitative analysis of TGFbeta2 expression in each group, a total of ten slices per group were evaluated, one-way ANOVA). a.u. represents arbitrary unit, **** represents $p<0.001$.

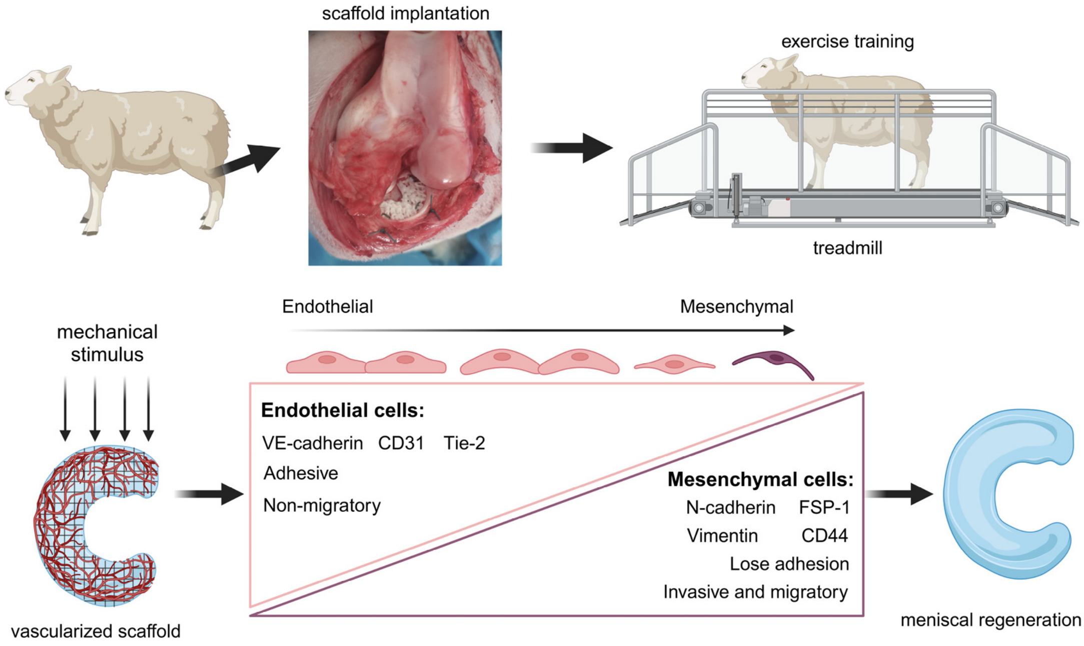

Supplemental Figure 32. The schematic diagram of mechanical stimulus on facilitating EndMT and consequent meniscal regeneration in an ovine subtotal meniscectomy model.

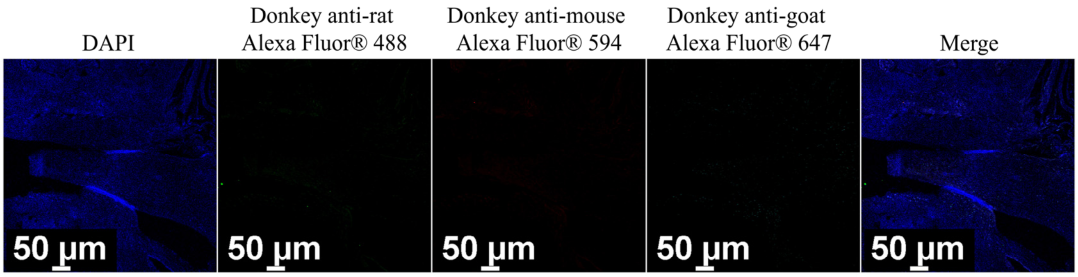

Supplemental Figure 33. The immunofluorescence negative control of *CDH5-CreERT2; Rosa26-LSL-Tdtomato* endothelial lineage tracing transgenic mice knee incubated with only secondary antibodies (Donkey anti-rat IgG H&L-Alexa Fluor®488, Donkey anti-mouse IgG H&L-Alexa Fluor®594, Donkey anti-goat IgG H&L-Alexa Fluor®647).
